# Epitope-Based Vaccine Target Screening against Highly Pathogenic MERS-CoV: An *In Silico* Approach Applied to Emerging Infectious Diseases

**DOI:** 10.1371/journal.pone.0144475

**Published:** 2015-12-07

**Authors:** Jiandong Shi, Jing Zhang, Sijin Li, Jing Sun, Yumei Teng, Meini Wu, Jianfan Li, Yanhan Li, Ningzhu Hu, Haixuan Wang, Yunzhang Hu

**Affiliations:** 1 Institute of Medical Biology, Chinese Academy of Medical Sciences and Peking Union Medical College, Kunming, China; 2 Yunnan Provincial Key Laboratory of Arbo Infectious Disease Control Research (Preparing), Institute of Medical Biology, Chinese Academy of Medical Sciences, Kunming, China; 3 Prenatal Diagnosis Center, Shijiazhuang Obstetrics and Gynecology Hospital, Shijiazhuang, Hebei, China; Saint Louis University, UNITED STATES

## Abstract

Middle East respiratory syndrome coronavirus (MERS-CoV) with pandemic potential is a major worldwide threat to public health. However, vaccine development for this pathogen lags behind as immunity associated with protection is currently largely unknown. In this study, an immunoinformatics-driven genome-wide screening strategy of vaccine targets was performed to thoroughly screen the vital and effective dominant immunogens against MERS-CoV. Conservancy and population coverage analysis of the epitopes were done by the Immune Epitope Database. The results showed that the nucleocapsid (N) protein of MERS-CoV might be a better protective immunogen with high conservancy and potential eliciting both neutralizing antibodies and T-cell responses compared with spike (S) protein. Further, the B-cell, helper T-cell and cytotoxic T lymphocyte (CTL) epitopes were screened and mapped to the N protein. A total of 15 linear and 10 conformal B-cell epitopes that may induce protective neutralizing antibodies were obtained. Additionally, a total of 71 peptides with 9-mer core sequence were identified as helper T-cell epitopes, and 34 peptides were identified as CTL epitopes. Based on the maximum HLA binding alleles, top 10 helper T-cell epitopes and CTL epitopes that may elicit protective cellular immune responses against MERS-CoV were selected as MERS vaccine candidates. Population coverage analysis showed that the putative helper T-cell epitopes and CTL epitopes could cover the vast majority of the population in 15 geographic regions considered where vaccine would be employed. The B- and T-cell stimulation potentials of the screened epitopes is to be further validated for their efficient use as vaccines against MERS-CoV. Collectively, this study provides novel vaccine target candidates and may prompt further development of vaccines against MERS-CoV and other emerging infectious diseases.

## Introduction

Middle East respiratory syndrome (MERS) is a newly emerging acute respiratory system infectious disease, which is characterised by acute pneumonia, respiratory failure, and renal failure and caused by MERS-coronavirus (MERS-CoV) [1,2]. MERS-CoV was first isolated and identified in Saudi Arabia in 2012 as a new member in the lineage C of the genus *Betacoronavirus*, and is a zoonotic virus that is transmitted from bats to camels, and then to humans [3,4,5]. The genome of MERS-CoV is a non-segmented positive-sense, single-stranded RNA (ssRNA) of approximately 30 kb encoding 11 proteins. They include two replicase polyproteins (open reading frames 1ab and 1a); four structural proteins including spike (S), envelope (E), membrane (M), and nucleocapsid (N) proteins; and five nonstructural proteins (open reading frames 3, 4a, 4b, 5, and 8b) [6]. MERS-CoV can be transmitted from person-to-person and has as high as 40% mortality [7]. As of June 26 2015, a total of 1356 cases including 484 related deaths have been reported to the World Health Organization [8]. No licensed vaccine or specific treatment is currently available.

As one of the most effective strategies to prevent virus infection, vaccination is considered to be indispensable especially in the absence of effective treatment drugs. The continuous increase of patients and a high mortality rate of MERS-CoV infection highlight the urgent need for safe and effective vaccines development. The majority of recent progresses focused on the viral S glycoprotein and receptor-binding domain (RBD) of S protein [9–19], and showed that S protein-based or RBD-based subunit or vector vaccine that contain multiple neutralizing epitopes with high potentials to induce strong neutralizing antibodies against MERS-CoV infection; thus, it is considered to be a promising target for effective MERS vaccine design [9–19]. Additionally, Almazan *et al*. constructed a full-length infectious cDNA clone of the MERS-CoV genome that lacked of E gene by reverse genetics approach to develop attenuated viruses as vaccine candidates [20]. Theoretically, nearly all viral proteins are potential immunogens and vaccine targets. Thus, in addition to the S protein, other viral proteins of the MERS-CoV might be effective immunogen targets of MERS vaccine. However, few studies have analyzed or tested the possibility of other viral proteins of MERS-CoV as vaccine targets. More importantly, T-cell-based cellular immunity is essential for cleaning MERS-CoV infection, yet the vaccine against the S protein mainly elicits neutralizing antibody response. Further, the high mutation rate of the S protein may result in the escape of neutralizing antibodies against MERS-CoV. Therefore, a highly conserved target that elicit both neutralizing antibody and cellular immunity against MERS-CoV is essential for an effective vaccine development.

Epitopes are also known as the antigenic determinant, which represents the minimal immunogenic region of a protein antigen and precisely elicit a specific immune responses [21]. Numerous studies show that epitope-based vaccines could effectively elicit protective immune responses against diverse pathogens, such as human immunodeficiency virus, influenza virus, hepatitis B virus, and hepatitis C virus [22–25]. Nevertheless, for emerging highly pathogenic pathogens, like MERS-CoV, H5N1, and H7N9, although their genome sequences are available in GenBank database, their immunity associated with protection is currently largely unknown. These gaps in understanding immune protection make developing vaccines against these highly pathogenic viruses more difficult and challenging [26]. Therefore, an immunoinformatics-driven approach to thoroughly screen the dominant immunogens based on available genome sequences data of pathogens is essential and promising for effective vaccine design of emerging infectious diseases [26].

In this study, based on newly available genome sequence data of MERS-CoV, numerous epitope vaccine candidates that could elicit protective humoral and cellular immune responses were obtained by immunoinformatics-driven vaccine target screening strategy. These results provided new vaccine epitope candidates for MERS vaccine development, and indicated that immunoinformatic-driven immunogen screening is a promising strategy to accelerate vaccine development of the emerging highly pathogenic pathogens.

## Materials and Methods

### Protein sequence retrieval

The entire viral proteome sequences of all MERS-CoV isolates that include structural proteins spike protein (S), envelope protein (E), membrane protein (M), nucleocapsid protein (N), and non-structural proteins ORF3, ORF4a, ORF4b, ORF5 and ORF8b, and two replicase polyproteins ORF1ab and ORF1a, were retrieved from the National Center for Biotechnology Information database (http://www.ncbi.nlm.nih.gov/) and used as an input for various bioinformatics tools for antigenicity assessment, secondary structure and 3D model prediction, and epitope prediction. These sequences come from different geographic regions such as Saudi Arabia, England, Qatar, Spain, Germany, Jordan, and Korea with time ranges from 2012 to 2015.

### Antigenic protein screening and structure analysis

Antigenicity refers to the ability of antigen that can be recognized by the immune system. Hence, to find the best potential candidate antigen, the VaxiJen v2.0 server [27] (http://www.ddg-pharmfac.net/vaxijen/VaxiJen/VaxiJen.html) was used for analyzing whole protein antigenicity and determined the most potent antigenic protein. The VaxiJen is a web server, that was developed by Dr. Doytchinova IA and Dr. Flower DR, from Faculty of Pharmacy, Medical University of Sofia, for prediction of protective antigens, tumor antigens and subunit vaccines, with prediction accuracy of 70% to 89% for the discrimination between antigens and non-antigens [27]. In this study, virus was chosen as the target organism with default parameter. A single antigenic protein with the highest antigenicity score was selected as vaccine candidate for further analysis. Antigenicity is closely related to the secondary and tertiary structure of the protein; hence, to determine the antigenicity and function of the target protein, the secondary structure of target protein that included solvent accessibility, transmembrane helices, globular regions, and coiled coil regions were predicted using the ExPASy’s secondary structure prediction server ProtParam [28] (http://web.expasy.org/protparam/) and a self-optimized prediction method for alignment (SOPMA) [29] (https://npsa-prabi.ibcp.fr/cgi-bin/npsa_automat.pl?page=/NPSA/npsa_sopma.html) with default parameter. ProtParam is a tool which allows the computation of various parameters including the molecular weight, theoretical pI, amino acid composition, atomic composition, extinction coefficient, estimated half-life, instability index, aliphatic index and grand average of hydropathicity (GRAVY) for a given protein. The SOPMA method correctly predicts 69.5% of the secondary structure α-helix, β-sheet and coil [29]. The RaptorX web server [30] (http://raptorx.uchicago.edu/) was used to predict the three-dimensional (3D) structure and binding residues of the chosen protein.

### B-cell epitope prediction

The method of Kolaskar and Tongaonkar [31] at the Immune Epitope Database (IEDB) (http://tools.immuneepitope.org/bcell/) was applied to predict linear B-cell epitopes. The Kolaskar and Tongaonkar method has been applied to a large number of proteins to predict B-cell epitopes by the developers. Their results showed that the method can predict epitopes with about 75% accuracy; hence, it is better than most of the known methods [31]. Further, the properties of the B-cell epitopes also include the flexibility, surface accessibility and hydrophilicity; hence, they were predicted by the Karplus and Schulz flexibility prediction [32] and Emini surface accessibility prediction [33] with a threshold of 1.0 respectively, of the IEDB. Conformational B-cell epitopes were predicted by ElliPro (http://tools.immuneepitope.org/ellipro/) [34] with the minimum score value set at 0.7 while the maximum distance selected as 6 Å. The ElliPro allows the prediction and visualization of B-cell epitopes in a given protein sequence or structure. The test for ElliPro on a benchmark dataset of discontinuous epitopes inferred from 3D structures of antibody-antigen complexes has shown the best performance compared with six other structure-based epitope prediction methods [34].

### Helper T-cell epitope prediction

The NetMHCIIpan 3.0 server [35] (http://www.cbs.dtu.dk/services/NetMHCIIpan/) was used to predict helper T-cell epitopes. Threshold for strong binding peptides (IC_50_) was set at 50 nM to determine the binding and interaction potentials of helper T-cell epitope peptide and major histocompatibility complex (MHC) class II allele. The NetMHCIIpan-3.0 is the first pan-specific helper T-cell epitope prediction method that covers all human leucocyte antigen (HLA) class II molecules including HLA-DR, HLA-DP, and HLA-DQ. It can predict peptide binding to any HLA class II molecule in a specified protein sequence [34]. The method was evaluated by the developers and shown a significant improvement over molecule-specific methods; hence, it is considered the most accurate MHC class II predictor [36]. Here, the top 10 epitopes with the maximum of binding HLA-DR alleles were selected as putative epitope candidates.

### CTL epitopes prediction

The cytotoxic T lymphocyte (CTL) epitope prediction was made using the IEDB analysis resource NetMHCpan (version 2.8) tool [37,38] and the Consensus tool [39] which combines predictions from ANN aka NetMHC (3.4) [40][41], SMM [42], and Comblib [43]. The NetMHCpan is a high-throughput computational method for genome- and HLA-wide prediction of peptide binding to HLA class I molecules, because it contains all HLA class I alleles. Hence, it can offer a truly global analysis for binding of the antigenetic peptide and HLA molecule and promote rational vaccine design [37]. The NetMHC is a prediction method with an average of 75–80% accuracy for peptides binding to HLA class I molecules. It has been employed widely to predict HLA-binding peptides in proteomes of various pathogens including SARS, Influenza and HIV [41]. The percentile rank threshold was set at 0.5. In this study, the top 10 epitopes with the maximum of binding HLA-A alleles were selected as putative epitope vaccine candidates. Further, except for a strong binding affinity, the peptides with strong immunogenicity are more likely to be CTL epitopes than those with weak immunogenicity. Hence, the IEDB immunogenicity prediction tool (http://tools.immuneepitope.org/immunogenicity/) was used to predict the immunogenicity of the candidate epitopes [44]. This tool predicts immunogenicity of a peptide-HLA (pHLA) complex based on the amino acid properties and their positions in the defined peptide.

### Conservancy analysis

The epitope conservancy analysis tool (http://tools.immuneepitope.org/tools/conservancy/iedb_input) at the IEDB was applied for the epitope conservancy analysis [45]. The conservancy levels were obtained by searching for identities in the given protein sequence. This tool calculates the degree of conservancy of an epitope within a given protein sequence set at different degrees of sequence identity. The degree of conservancy is defined as the fraction of protein sequences containing the epitope at a given identity level.

### Allergenicity assessment

The allergenicity of the epitopes was analyzed by the AllerHunter server [46] (http://tiger.dbs.nus.edu.sg/AllerHunter). AllerHunter is a SVM-pairwise system for assessment of allergenicity and allergic cross-reactivity in proteins. It aims to predict allergens and non-allergens with high sensitivity and specificity, without compromising efficiency at classification of proteins with similar sequences to known allergens [46].

### Population coverage prediction

Due to the MHC restriction of T cell response, the peptides with more different HLA binding specificities mean more population coverage in defined geographical regions where the peptide-based vaccine might be employed. The population coverage rate of individual epitope was calculated using the IEDB population coverage tool (http://tools.immuneepitope.org/tools/population/iedb_input) [47].

Every epitope and its binding HLA alleles were added, and different geographic areas were also selected.

## Results

### Antigenic protein identification and structure analysis

**Fig 1:**
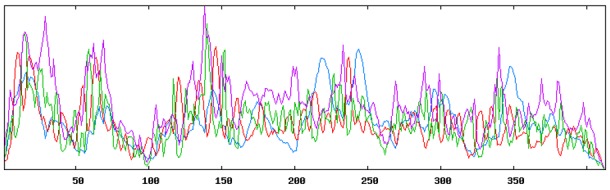
Secondary structure plot of the highest antigenic nucleocapsid (N) protein of MERS-CoV. Here, helix is indicated by blue, while extended strands, beta turns and random coils are indicated by red, green and yellow, respectively.

**Fig 2:**
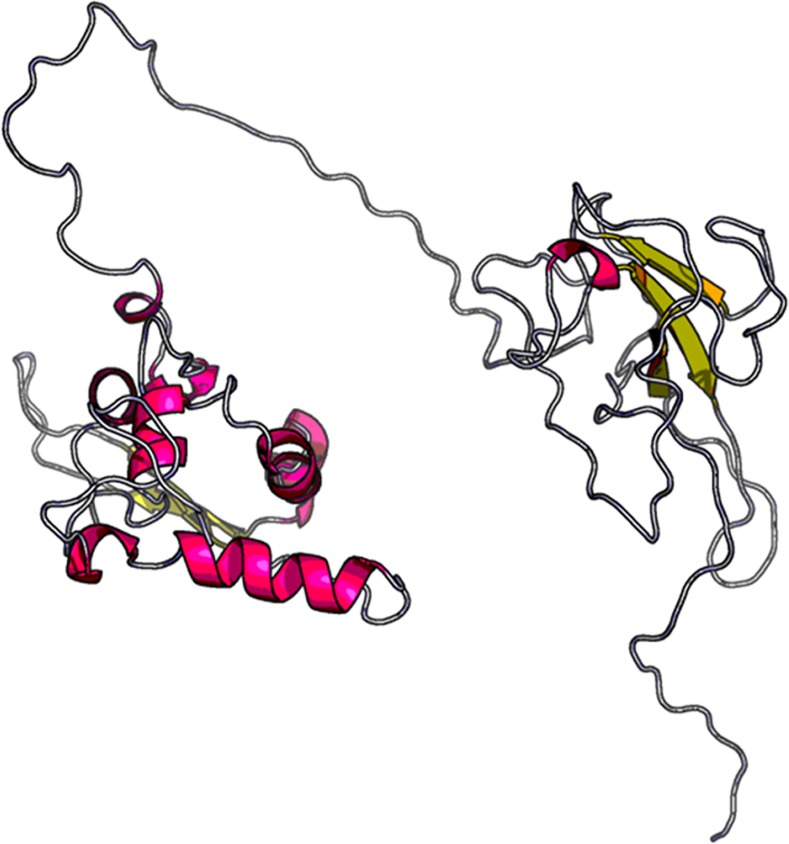
A 3D model of the highest antigenic nucleocapsid (N) protein of MERS-CoV that were modeled by using RaptorX web server.

The selection of immunogen is the first step for vaccine design; hence, to obtain the most probable antigenic protein, the whole viral proteomes of all MERS-CoV isolates were retrieved and screened. A total of 99 MERS-CoV variants from different geographical regions with their entire proteomes were obtained (S1 Table). The antigenicity of each protein is indicated by the overall score produced by the specific protein sequence using the VaxiJen server. On the whole, the scores of the N protein and ORF8b protein of MERS-CoV were higher than the S, E, M, ORF3, ORF4a, ORF4b, ORF5, ORF1ab and ORF1a proteins. Moreover, the ORF8b protein with its Genbank ID: AIL23997.1 and the N protein with its Genbank ID: AGV08499.1 possessed the significantly highest antigenic scores of 0.8218 and 0.7749, respectively, among all the query proteins (S1 Table). However, the ORF8b protein would be rapidly degraded by proteasomes in the absence of the ORF8a protein, an accessory protein of ORF8b [48]. Thus, it was not an ideal antigen candidate although its high antigenic score. In this study, the N protein was chosen as a candidate immunogen and model protein to carry out epitope-based vaccine design. Further, the most probable antigenic protein was analyzed for its secondary structural characteristics, and the properties that included total length of 413 aa, molecular weight of 45048.2 Da, theoretical pI of 10.05, formula of C_1965_H_3102_N_594_O_611_S_7_, 70 alpha helixes, 66 extended strands, 32 beta turns, and 245 random coils are obtained and shown in Table 1 and Fig 1. The grand average of the hydrophobicity rule (GRAVY) of the N protein linear sequence was predicted to be negative (-0.865). This indicated the property of the protein as hydrophilic in nature and most of the residues to be present on the surface. This means that more amino acids tend to be binding residues when interacted with other proteins. The 3D structure showed a maximum of 64% identity of the N protein of MERS-CoV with the best template protein PDB: 2ofzA (RNA Binding Domain of Sars Nucleocapsid Protein), and it is composed of two separate domains or pockets (Fig 2). Protein binding site prediction showed that a total of 12 binding residues including T40, V41, S42, Y44, T45, G46, R97, Y99, Y101, R138, A145 and S173 were mapped on the domain-1. And only 2 binding residues including G267 and L268 were mapped on the domain-2. Obviously, the domain-1 possessed the greater ability to interact with other proteins than the domain-2. This might be associated with distribution of the conformational epitopes on the N protein of MERS-CoV.

**Table 1:** Secondary structural characteristics of the highest antigenic N protein (Genbank ID: AGV08499.1) of MERS-CoV.

Criteria	Assessment
Number of Amino Acids	413
Molecular Weight	45048.2 Da
Theoretical pI	10.05
Total number of negatively charged residues (Asp + Glu)	33
Total number of positively charged residues (Arg + Lys)	55
Formula	C_1965_H_3102_N_594_O_611_S_7_
Extinction coefficients	47900
Estimated half-life	30 hours (mammalian reticulocytes, in vitro)
Instability index	48.62
Aliphatic index	56.76
Grand average of hydropathicity (GRAVY)	-0.865

### Identification of B-cell epitopes

**Fig 3:**
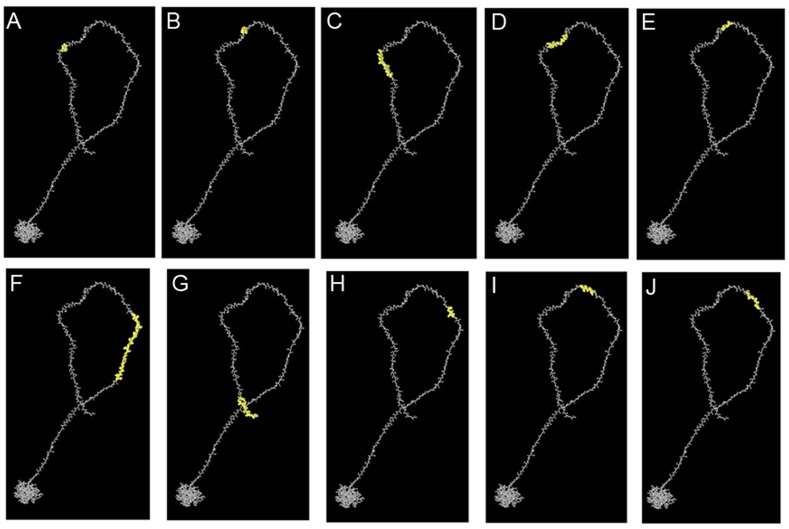
3D representation of conformational epitopes (A to J) of the highest antigenic nucleocapsid (N) protein of MERS-CoV. The epitopes are represented by yellow surface, and the bulk of the N protein is represented in grey sticks.

One of the key steps in epitope-driven vaccine design is the prediction and identification of the B-cell epitopes in target antigens. Thus, to obtain B-cell epitope candidates in the N protein of MERS-CoV, *in silico* identification of B-cell epitopes based on the IEDB database was performed. Based on the Kolaskar and Tongaonkar’s method of the IEDB, a total of 15 linear B-cell epitopes of the N protein of MERS-CoV were identified. The length of the epitopes ranged from 6 to 20 amino acids. These epitopes had 78–91% conservancy level among the specified N protein sequences. Notably, the epitopes ^4^PAAPRAVSF^12^ and ^324^NPVYFLRYSGAIKL^337^ were allergic to human; hence, they could not be vaccine candidates. Epitopes’ length, sequences, location, conservancy, and allergenicity are shown in Table 2. Further, the results showed that the average antigenic prospensity value of the predicted epitopes was 0.993 with a minimum of 0.863 and a maximum of 1.182 (S1 Fig). Since surface accessibility and fragment flexibility are also key features for predicting B-cell epitopes. Thus, the surface accessibility and flexibility were analyzed based on methods of the IEDB. Results of the analysis of the surface accessibility of the predicted peptides showed that the maximum surface probability value was 6.971 at amino acid position from 363 to 368. The sequence of the hexapeptide is ^363^KKEKKQ^368^, where 365E is the surface residue. The minimum value of surface probability is 0.074 for peptides ^205^GIGAVG^210^, where 207G is the surface residue (S2 Fig). Likewise, results of the analysis of the flexibility of the predicted peptides showed that the maximum flexibility value was 1.160 at amino acid position from 170 to 176, and its sequence is ^167^GNSQSSS^173^, where 170Q is the flexible residue. The minimum value of flexibility probability is 0.903 for peptides ^97^RWYFYYT^103^, where 100F is the flexibility residue (S3 Fig). In addition, a total of 10 conformational B-cell epitopes having a Protrusion Index (PI) score value above 0.7 were obtained by the ElliPro. The highest probability of a conformational epitope was calculated at 97.9% (PI score: 0.979) and shown in Fig 3A. Residues involved in conformational epitopes, their location, number of residues, and scores are shown in Table 3, whereas, their positions on 3D structures are shown in Fig 3A to 3J.

**Table 2:** Predicted linear B-cell epitopes of the N protein of MERS-CoV with their conservancy and allergenicity.

Epitope No.	Start	End	Sequence	Length	Conservancy (%)	Allergenicity
1	4	12	PAAPRAVSF	9	87	Yes
2	42	47	SWYTGL	6	91	No
3	50	58	HGKVPLTFP	9	88	No
4	60	66	GQGVPLN	7	91	No
5	92	102	KQLAPRWYFYY	11	90	No
6	108	126	EAALPFRAVKDGIVWVHED	19	78	No
7	144	150	SAIVTQF	7	88	No
8	172	180	SSRASSVSR	9	87	No
9	206	225	IGAVGGDLLYLDLLNRLQAL	20	89	No
10	230	240	VKQSQPKVITK	11	89	No
11	261	268	NMVQAFGL	8	90	No
12	296	304	IAELAPTAS	9	89	No
13	324	337	NPVYFLRYSGAIKL	14	90	Yes
14	347	353	WLELLEQ	7	90	No
15	399	405	RPSVQPG	7	89	No

**Table 3:** Predicted conformational B-cell epitopes of the N protein of MERS-CoV.

Epitope No.	Residues and their positions	No. of Residues	Score	3D Structure
1	K346, W347, L348, E349	4	0.979	Fig 3A
2	Y327, F328, L329, R330	16	0.893	Fig 3B
3	L350, L351, E352, Q353, N354, I355, D356, A357, Y358, K359, T360, F361, P362, K363, K364, E365	15	0.887	Fig 3C
4	Y331, S332, G333, A334, I335, K336, L337, D338, P339, K340, N341, P342, N343, Y344, N345	7	0.881	Fig 3D
5	D320, D321, H322, G323,N324, P325, V326	41	0.867	Fig 3E
6	H252, K253, R254, T255, S256, T257, K258, S259, F260, N261, M262, V263, Q264, A265, F266, G267, L268, R269, G270, P271, G272, D273, L274, Q275,G276, N277, F278, G279, D280, L281, Q282, L283, N284, K285, L286, G287, T288, E289, D290, P291, R292	15	0.861	Fig 3F
7	R399, P400, S401, V402, Q403, P404, G405, P406, M407, I408, D409, V410, N411, T412, D413	6	0.794	Fig 3G
8	W293, P294, Q295, I296, A297, E298	8	0.778	Fig 3H
9	F312, K313, L314, T315, H316, Q317, N318, N319	13	0.772	Fig 3I
10	L299, A300, P301, T302, A303, S304, A305, F306, M307, G308, M309, S310, Q311	16	0.709	Fig 3J

### Identification of helper T-cell epitopes

Since MHC-II restricted CD4^+^ T-cells activation is important for inducing and maintaining an efficient antibody response or CTL response, hence, the helper T-cell epitopes in the N protein of MERS-CoV were *in silico* identified. As a result, a total of 71 antigenic peptides with 9-mer core sequences in the N protein of MERS-CoV were identified to be helper T-cell epitopes using the NetMHCIIpan 3.0 server (S2 Table). They can bind a different large number of HLA-DR alleles with an IC_50_ value less than 50nM, which indicated a very high binding affinity to HLA-DR molecules. Based on the fact that a good T-cell epitope should interact with as many HLA alleles as possible, the top 10 epitopes with the highest number of binding HLA-DR alleles were selected as putative helper T-cell epitope candidates (Table 4). All of the 10 putative helper T-cell epitopes with numerous binding HLA-DR alleles tend to be good epitope candidates. Among them, the epitope ^329^LRYSGAIKL^337^ interacting with 357 HLA-DR alleles was the epitope possessing the maximum number of binding HLA-DR alleles. On the contrary, ^230^VKQSQPKVI^238^ interacting with 94 HLA-DR alleles is the epitope possessing the minimum number of binding HLA-DR alleles. Further, all selected putative epitopes were highly conserved with 89–91% conversancy level, and no epitope was found allergic to humans. Details of predicted putative helper T-cell epitopes along with their respective binding HLA-DR alleles are shown in S2 Table.

**Table 4:** Helper T-cell epitopes of the N protein of MERS-CoV selected on the basis of maximum HLA binding alleles.

Epitope core sequence (9 -mer)	Position	No. of binding HLA-DR alleles	Conservancy (%)	Allergenicity
FNMVQAFGL	260–268	173	90	No
LRYSGAIKL	329–337	356	91	No
LQALESGKV	222–230	139	89	No
LNRLQALES	219–227	144	90	No
FMGMSQFKL	306–314	100	91	No
LYLDLLNRL	214–222	160	90	No
IKQLAPRWY	91–99	154	89	No
FLRYSGAIK	328–336	142	91	No
LLYLDLLNR	213–221	110	90	No
VKQSQPKVI	230–238	94	89	No

### Identification of CTL epitopes

As we all know, MHC-I restricted CD8^+^ cytotoxic T lymphocytes (CTLs) plays a crucial role in controlling virus infection. Hence, identification of CTL epitopes is vital for understanding the principles of T cell activation and epitope-driven vaccine design. Herein, a total of 34 immunogenic peptides with 9-mer sequences in the N protein of MERS-CoV were identified to be CTL epitopes using the MHC-I binding predictions of the IEDB with recommended methods (S3 Table). They can bind a different large number of HLA-A alleles with a very high binding affinity. Top 10 epitopes binding the most HLA-A alleles were selected as putative CTL epitope candidates based on their more broad HLA coverage (Table 5). Among them, the epitope ^92^KQLAPRWYF^100^ had the highest number of binding HLA-A alleles (138 alleles), followed by ^343^NYNKWLELL^351^ (128 alleles). Yet, the epitopes ^72^AQNAGYWRR^80^ (37 alleles) and ^387^RVQGSITQR^395^ (31 alleles) had the minimum number of binding HLA-A alleles. Most CTL epitope candidates with a positive score of immunogenicity showed a high potential to elicit strong CTL response. Further, these CTL epitope candidates were highly conserved with 85–91% conservancy level among available N protein sequences of MERS-CoV isolates, and no epitope was found allergic to humans. Details of predicted putative CTL epitopes along with their respective binding HLA-A alleles are shown in S3 Table.

**Table 5:** CTL epitopes of the N protein of MERS-CoV selected on the basis of maximum HLA binding alleles.

Epitope sequence (9 -mer)	Position	No. of binding HLA-A alleles	Immunogenicity	Conservancy (%)	Allergenicity
AQNAGYWRR	72–80	37	0.28397	91	No
LAPRWYFYY	94–102	34	0.35734	90	No
NYNKWLELL	343–351	128	0.06418	90	No
KQLAPRWYF	92–100	138	0.25847	90	No
ELAPTASAF	298–306	53	-0.04059	89	No
NTVSWYTGL	39–47	45	0.11171	90	No
STPAQNAGY	69–77	60	-0.0303	91	No
QLAPRWYFY	93–101	49	0.32573	90	No
HGNPVYFLR	322–330	76	0.11578	89	No
RVQGSITQR	387–395	31	-0.07424	85	No

### Population coverage of the epitopes

HLA distribution varies among different ethnic groups and geographic regions around the world. Thus, population coverage must be taken into account when designing an effective vaccine to cover as much as possible populations. In this study, all indicated alleles in supplementary data were identified as optimum binders with the predicted epitopes and were used to determine the population coverage for these epitopes. The high population coverage was found for all putative helper T-cell epitopes and CTL epitopes in 15 specified geographic regions of the world (Table 6). For helper T-cell epitopes, an excellent percentage of cumulative population coverage of the 10 epitopes was obtained in South Asia (98.68%), closely followed by Central Africa (98.51%), Southeast Asia (98.17%), West Africa (98.10%), East Africa (97.75%), Southwest Asia (97.45%), Oceania (96.78%), West Indies (96.57%), Northeast Asia (96.20%), Europe (95.77%), North Africa (95.05%), North America (94.81%), East Asia (93.89%), and South America (87.18%). And the lowest was in South Africa (62.18%). These results indicated most geographic regions of the world, where more than 90% of their population can be covered by all putative helper T-cell epitopes. For CTL epitopes, a moderate and acceptable percentage of cumulative population coverage of the 10 epitopes was obtained in Central Africa (59.49%), followed by East Asia (50.14%), Oceania (48.85%), Southwest Asia (48.21%), South Asia (47.21%), East Africa (42.51%), Southeast Asia (42.01%), West Africa (41.86%), South Africa (38.95%), North America (35.03%), North Africa (33.02%), South America (32.71%), Northeast Asia (32.47%), and West Indies (30.08%). And the lowest was in Europe (20.62%). These results indicated only partial regions of the world, such as Central Africa and East Asia, where approximately half of the population can be covered by all putative CTL epitopes. Overall, these results suggested that putative helper T-cell epitopes and CTL epitopes can specifically bind with the prevalent HLA molecules in the target population where the vaccine will be employed.

**Table 6:** Population coverage rate (%) for all putative helper T-cell epitopes and CTL epitopes of the N protein of MERS-CoV.

Population/Area	Population coverage (%)	
	All putative helper T-cell epitopes	All putative CTL epitopes
East Asia	93.89	50.14
Northeast Asia	96.20	32.47
Sourth Asia	98.68	47.21
Southeast Asia	98.17	42.01
Southwest Asia	97.45	48.21
Europe	95.77	20.62
East Africa	97.75	42.51
West Africa	98.10	41.86
Central Africa	98.51	59.49
North Africa	95.05	33.02
South Africa	62.18	38.95
West Indies	96.57	30.08
North America	94.81	35.03
South America	87.18	32.71
Oceania	96.78	48.85

## Discussion

Emerging infectious pathogens such as MERS-CoV, H5N1, and H7N9 are highly pathogenic for humans. For these pathogens, immunity associated with protection remain largely unknown. Gaps in understanding the protective immunity against these pathogens make developing vaccines for newly emerging infectious diseases more difficult and challenging [26]. Thus, the genome-wide screening of vaccine targets based on newly available genome sequences data of pathogens is essential and urgent for developing efficiently vaccines against these newly emerging highly pathogenic pathogens.

This study aim to screen and investigate the most antigenic protein of the MERS-CoV and to find out the B- and T-cell epitopes that were mapped on the most antigenic protein by using a strategy namely genome-wide screening of vaccine epitopes. Based on advances in bioinformatics, the immunoinformatics approach could be an effective and essential strategy for vaccine development of emerging highly pathogenic pathogens. In this study, an immunoinformatics-driven approach was used to screen vital dominant immunogens against MERS-CoV. The results showed that the N protein was a better antigenic protein with the highest antigenic scores compared with other viral proteins in entire viral proteome. However, nearly all current studies of MERS vaccine focused on the S protein of MERS-CoV [9–19], because the S protein mediated recognition of host cell DPP4 receptor for MRES-CoV and induced significant immune response [49]. In fact, not just the S protein, other MERS-CoV viral proteins might be effective MERS vaccine candidates. However, few studies have emphasized this possibility. Further, T-cell-based cellular immunity is essential for cleaning MERS-CoV infection, yet the vaccine against the S protein mainly elicit neutralizing antibody response. More importantly, high mutation rate of the S protein of MERS-CoV may cause escape of neutralizing antibodies against S protein. Therefore, an ideal target should be highly conserved that elicit both neutralizing antibody and cellular immunity against MERS-CoV, which is more essential for an effective MERS vaccine development. The N protein of human CoV is abundantly produced during infection and exhibits strong immunogenicity and conservancy, which can act as an ideal immunogen to elicit both cellular and humoral immune responses [50]. It is noteworthy that a large number of previous studies have shown the feasibility of the N protein as an immune target antigen or vaccine product [51–60]. Ma *et al*. demonstrated that a SARS-CoV vaccine based on the N gene that was expressed by DNA plasmid and adenovirus vector could induce detectable antibody and IFN-γ [51]. Similar studies of the DNA vaccine based on the SARS-CoV N gene have shown potential inducing specific humoral and cellular immunity in BALB/c mice [52,53]. Moreover, multiple immunodominant B-cell epitopes, helper T-cell epitopes and CTL epitopes were mapped on the N protein of SARS-CoV [54–58]. Additionally, the antigenicity of the N protein from other viruses has been demonstrated [59,60]. And the antibodies against the N proteins of diverse human CoVs have been revealed by Gao *et al*. [61]. Based on these findings, it concludes that the N protein of MERS-CoV might be a putative and valuable immunogen for vaccine development. Further, the screened epitopes from the N protein in this study also may be some valuable epitope-based vaccine candidates for further *in vitro* and *in vivo* tests for their antigenic and immunogenetic potentials.

The purpose of vaccination is to induce immunity against specific pathogens by selectively stimulating antigen-specific B-cells or CTLs, and helper T-cells. Theoretically, a vaccine should contain two classes of antigenic epitopes: a helper T-cell epitope and a B-cell epitope or a CTL epitope. Based on a combination of these epitopes, the vaccine is able to either induce specific humoral or cellular immune against specific pathogens [21]. Therefore, the B-cell, CTL, and helper T-cell epitopes were screened systemically in the N protein of MERS-CoV to obtain putative epitope vaccine candidates. In this study, one B-cell epitope, ^108^EAALPFRAVKDGIVWVHED^126^, showed a lower conservancy of 78% among different MERS-CoV isolates compared with other 14 epitopes, suggesting that it might be not an idea epitope candidate based on the fact that an epitope should be as conservative as possible to provide broader protection among different virus strains. In addition, various continuous and discontinuous B-cell epitopes were mapped on the N protein (Table 2 and Fig 3). All discontinuous B-cell epitopes were located on the surface of the domain-2 of the N protein, showing the accessibility for the entered virus. Moreover, the diverse T-cell epitopes including helper T-cell epitopes and CTL epitopes were delineated by dissecting the N proteins (Tables 4 and 5). Notably, neutralizing antibodies are not far enough to completely clean infectious pathogens. CTLs are needed because they play a central role in the generation of a protective immune response against diverse pathogen infection [62]. In this study, although the CTL epitope ^93^QLAPRWYFY^101^ showed the highest immunogenic score of 0.32573, it possessed the minimal HLA binding alleles among all predicted CTL epitopes. On the contrary, the epitope ^92^KQLAPRWYF^100^ had the maximum HLA binding alleles, but less immunogenic score of 0.25847. This inconsistency of immunological characteristics among different epitopes suggested that various parameters needed to be considered when screened epitopes. Helper T-cell epitopes are critical to the generation of vigorous humoral and CTL responses. However, the response to T-cell epitopes is restricted by HLA proteins. Moreover, HLA is highly polymorphic in diverse ethnic populations. Therefore, to induce broad immune responses in diverse ethnic genetically diverse human populations, the HLA specificity of T-cell epitopes must be considered first as a major criteria for screening of the epitopes [63,64]. Based on the above analysis, to get more population coverage, the epitope candidates should bind more HLA alleles. In this study, the 10 ten helper T-cell epitopes and CTL epitopes that bind the maximum number of HLA alleles were selected as putative vaccine candidates. Further analysis revealed that all putative helper T-cell and CTL epitopes have an ideal population coverage and may provide broad immune protection for different geographic regions around the world. Additionally, as an ideal epitope, it should be highly conserved among different MERS-CoV isolates. Thus, conservancy level should be determined for all putative epitope candidates. The epitopes from this study were highly conserved in designated MERS-CoV isolates, suggesting that they could be ideal epitope vaccine candidates to elicit protective neutralizing antibodies and cellular immune responses against MERS-CoV.

## Conclusions

In conclusion, this study indicated that immunoinformatics-driven genome-wide screening of vaccine targets of emerging highly pathogenic pathogens is a promising strategy to accelerate their vaccine development. Based on this strategy, the B-cell epitopes, helper T-cell epitopes and CTL epitopes in the N protein of MERS-CoV were mapped and selected as putative MERS vaccine candidates. However, the B-and T-cell stimulation potentials of the screened epitopes are needed to be tested by *in vitro* and *in vivo* experiments along with this *in silico* study for their efficient use as vaccines against MERS-CoV. The present study provides new and valuable epitope candidates and prompts the future vaccine development of MERS and other emerging infectious diseases.

## Supporting Information

S1 Fig Kolaskar and Tongaonkar’s antigenicity prediction of the N protein of MERS-CoV. X-axis represents the amino acids, whereas Y-axis represents the antigenic propensity. Average antigenic score was 0.993. Area displayed by the threshold line is considered as potential B-cell antigenic regions.
(PDF)

S2 Fig Emini surface accessibility prediction of the N protein of MERS-CoV. X-axis represents the amino acids, whereas Y-axis represents accessible propensity. Average antigenic score was 1.000. Area displayed by the threshold line is considered as potential B-cell accessible regions.
(PDF)

S3 Fig Karplus and schulz flexibility prediction of the N protein of MERS-CoV. X-axis represents the amino acids, whereas Y-axis represents flexible propensity. Average antigenic score was 1.028. Area displayed by the threshold line is considered as potential B-cell flexible regions.
(PDF)

S1 Table Antigenic scores of the entire proteomes of all MERS-CoV isolates. (XLS)

S2 Table 9-mer peptide core sequences in the N protein of MERS-CoV are predicted to be helper T-cell epitopes using the NetMHCIIpan 3.0 web server. (DOC)

S3 Table 9-mer peptide sequences in the N protein of MERS-CoV are predicted to be CTL epitopes using the IEDB MHC-I binding prediction web server. (DOC)
