# Supplementary material for: Epitope-Based Vaccine Target Screening against Highly Pathogenic MERS-CoV: An In Silico Approach Applied to Emerging Infectious Diseases
Source: PLoS One. 2015 Dec 7;10(12):e0144475. doi: 10.1371/journal.pone.0144475 (PMC4671582; doi:10.1371/journal.pone.0144475)
Supplement: S1 Fig — X-axis represents the amino acids, whereas Y-axis represents the antigenic propensity. Average antigenic score was 0.993. Area displayed by the threshold line is considered as potential B-cell antigenic regions. (PDF) [file pone.0144475.s001.pdf]

IEDB Analysis Resource

Home | Help | Example | Reference | Download | Contact

Kolaskar & Tongaonkar Antigenicity Prediction

Input Sequences

1 MASPAAPRAV SFADNNDITN TNLSRGRGRN PKPRAAPNNT VSWYTGLTQH GKVPLTFPPG
61 QGVPLNANST PAQNAGYWRR QDRKINTGNG IKQLAPRWYF YYTGTGPEAA LPFRAVKDGI
121 VWVHEDGATD APSTFGTRNP NNDSAIVTQF APGTKLPKNF HIEGTGGNSQ SSSRASSVSR
181 NSSRSSSQGS RSGNSTRGTS PGPSGIGAVG GDLLYLDLLN RLQALESGKV KQSQPKVITK
241 KDAAAAKNKM RHKRTSTKSF NMVQAFGLRG PGDLQGNFGD LQLNKLGTED PRWPQIAELA
301 PTASAFMGMS QFKLTHQNND DHGNPVYFLR YSGAIKLDPK NPNYNKWLEL LEQNIDAYKT
361 FPKKEKKQKA PKEESTDQMS EPPKEQRVQG SITQRTRTRP SVQPGPMIDV NTD

Center position: 4 **Window size:** 7 **Threshold:** 1.0 Recalculate

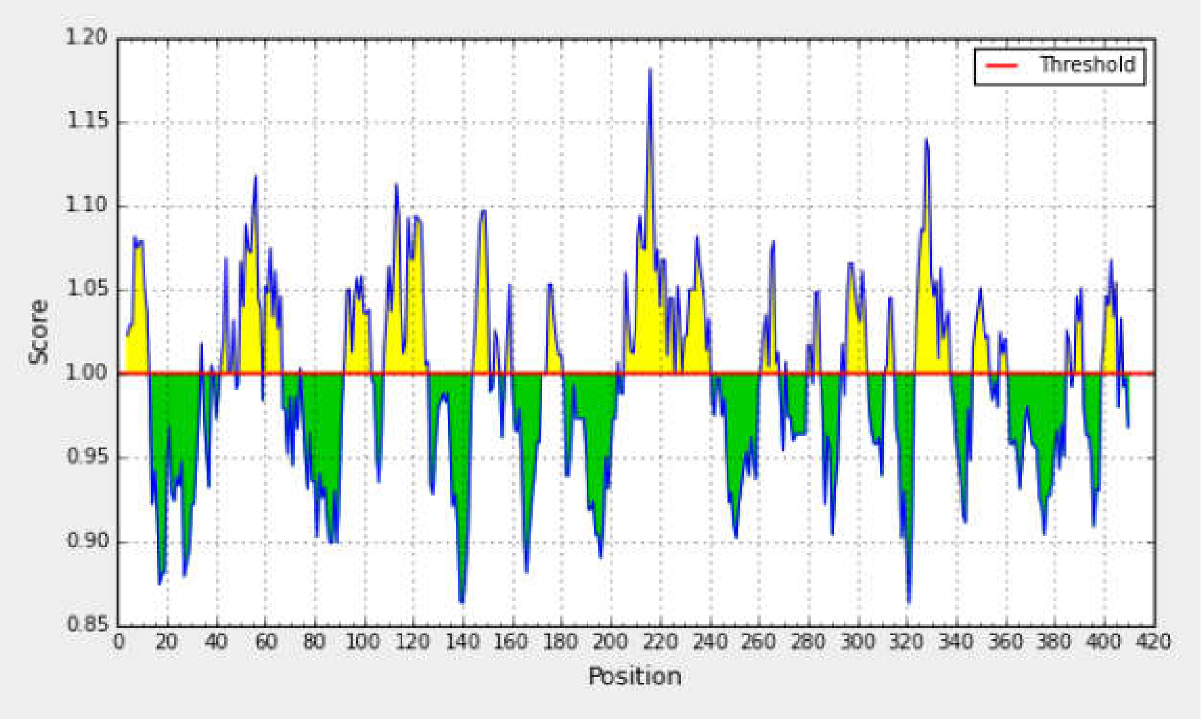

Average: 0.993 **Minimum:** 0.863 **Maximum:** 1.182

Predicted peptides:

No.	Start	End	Peptide	Length
1	4	12	PAAPRAVSF	9
2	42	47	SWYTGL	6
3	50	58	HGKVPLTFP	9
4	60	66	GQGVPLN	7
5	92	102	KQLAPRWYFYY	11
6	108	126	EAALPFRAVKDGIVWVHED	19
7	144	150	SAIVTQF	7
8	172	180	SSRASSVSR	9

No.	Start	End	Peptide	Length
9	206	225	IGAVGGDLLYLDLLNRLQAL	20
10	230	240	VKQSQPKVITK	11
11	261	268	NMVQAFGL	8
12	296	304	IAELAPTAS	9
13	324	337	NPVYFLRYSGAIKL	14
14	347	353	WLELLEQ	7
15	399	405	RPSVQPG	7

Predicted residue scores:

Position	Residue	Start	End	Peptide	Score
4	P	1	7	MASPAAP	1.023
5	A	2	8	ASPAAPR	1.029
6	A	3	9	SPAAPRA	1.029
7	P	4	10	PAAPRAV	1.082
8	R	5	11	AAPRAVS	1.075
9	A	6	12	APRAVSF	1.079
10	V	7	13	PRAVSFA	1.079
11	S	8	14	RAVSFAD	1.05
12	F	9	15	AVSFADN	1.037
13	A	10	16	VSFADNN	0.995
14	D	11	17	SFADNND	0.922
15	N	12	18	FADNNDI	0.942
16	N	13	19	ADNNDIT	0.916
17	D	14	20	DNNDITN	0.874
18	I	15	21	NNDITNT	0.881
19	T	16	22	NDITNTN	0.881
20	N	17	23	DITNTNL	0.948
21	T	18	24	ITNTNLS	0.969
22	N	19	25	TNTNLSR	0.929
23	L	20	26	NTNLSRG	0.924
24	S	21	27	TNLSRGR	0.938
25	R	22	28	NLSRGRG	0.933
26	G	23	29	LSRGRGR	0.947
27	R	24	30	SRGRGRN	0.879
28	G	25	31	RGRGRNP	0.887
29	R	26	32	GRGRNPK	0.895
30	N	27	33	RGRNPKP	0.922
31	P	28	34	GRNPKPR	0.922
32	K	29	35	RNPKPRA	0.949
33	P	30	36	NPKPRAA	0.976
34	R	31	37	PKPRAAP	1.018

Position	Residue	Start	End	Peptide	Score
35	A	32	38	KPRAAPN	0.976
36	A	33	39	PRAAPNN	0.954
37	P	34	40	RAAPNNT	0.932
38	N	35	41	AAPNNTV	1.005
39	N	36	42	APNNTVS	0.998
40	T	37	43	PNNTVSW	0.973
41	V	38	44	NNTVSWY	0.987
42	S	39	45	NTVSWYT	1.006
43	W	40	46	TVSWYTG	1.02
44	Y	41	47	VSWYTGL	1.069
45	T	42	48	SWYTGLT	1.001
46	G	43	49	WYTGLTQ	1.002
47	L	44	50	YTGLTQH	1.032
48	T	45	51	TGLTQHG	0.991
49	Q	46	52	GLTQHGK	0.994
50	H	47	53	LTQHGKV	1.067
51	G	48	54	TQHGKVP	1.04
52	K	49	55	QHGKVPL	1.089
53	V	50	56	HGKVPLT	1.074
54	P	51	57	GKVPLTF	1.072
55	L	52	58	KVPLTFP	1.099
56	T	53	59	VPLTFPP	1.118
57	F	54	60	PLTFPPG	1.045
58	P	55	61	LTFPPGQ	1.038
59	P	56	62	TFPPGQG	0.984
60	G	57	63	FPPGQGV	1.052
61	Q	58	64	PPGQGVP	1.048
62	G	59	65	PGQGVPL	1.075
63	V	60	66	GQGVPLN	1.034
64	P	61	67	QGVPLNA	1.061
65	L	62	68	GVPLNAN	1.027
66	N	63	69	VPLNANS	1.046
67	A	64	70	PLNANST	0.979
68	N	65	71	LNANSTP	0.979
69	S	66	72	NANSTPA	0.952
70	T	67	73	ANSTPAQ	0.986
71	P	68	74	NSTPAQN	0.945
72	A	69	75	STPAQNA	0.986
73	Q	70	76	TPAQNAG	0.967
74	N	71	77	PAQNAGY	1.003
75	A	72	78	AQNAGYW	0.978
76	G	73	79	QNAGYWR	0.951

Position	Residue	Start	End	Peptide	Score
77	Y	74	80	NAGYWRR	0.931
78	W	75	81	AGYWRRQ	0.965
79	R	76	82	GYWRRQD	0.936
80	R	77	83	YWRRQDR	0.936
81	Q	78	84	WRRQDRK	0.903
82	D	79	85	RRQDRKI	0.94
83	R	80	86	RQDRKIN	0.926
84	K	81	87	QDRKINT	0.932
85	I	82	88	DRKINTG	0.911
86	N	83	89	RKINTGN	0.899
87	T	84	90	KINTGNG	0.899
88	G	85	91	INTGNGI	0.93
89	N	86	92	NTGNGIK	0.899
90	G	87	93	TGNGIKQ	0.933
91	I	88	94	GNGIKQL	0.982
92	K	89	95	NGIKQLA	1.009
93	Q	90	96	GIKQLAP	1.05
94	L	91	97	IKQLAPR	1.05
95	A	92	98	KQLAPRW	1.013
96	P	93	99	QLAPRWY	1.046
97	R	94	100	LAPRWYF	1.057
98	W	95	101	APRWYFY	1.044
99	Y	96	102	PRWYFYY	1.058
100	F	97	103	RWYFYYT	1.036
101	Y	98	104	WYFYYTG	1.036
102	Y	99	105	YFYYTGT	1.038
103	T	100	106	FYYTGTG	0.997
104	G	101	107	YYTGTGP	0.993
105	T	102	108	YTGTGPE	0.949
106	G	103	109	TGTGPEA	0.935
107	P	104	110	GTGPEAA	0.957
108	E	105	111	TGPEAAL	1.011
109	A	106	112	GPEAALP	1.033
110	A	107	113	PEAALPF	1.064
111	L	108	114	EAALPFR	1.037
112	P	109	115	AALPFRA	1.067
113	F	110	116	ALPFRAV	1.113
114	R	111	117	LPFRAVK	1.094
115	A	112	118	PFRAVKD	1.039
116	V	113	119	FRAVKDG	1.012
117	K	114	120	RAVKDGI	1.02

Position	Residue	Start	End	Peptide	Score
118	D	115	121	AVKDGIV	1.093
119	G	116	122	VKDGIVW	1.069
120	I	117	123	KDGIVWV	1.069
121	V	118	124	DGIVWVH	1.094
122	W	119	125	GIVWVHE	1.092
123	V	120	126	IVWVHED	1.09
124	H	121	127	VWVHEDG	1.051
125	E	122	128	WVHEDGA	1.005
126	D	123	129	VHEDGAT	1.007
127	G	124	130	HEDGATD	0.934
128	A	125	131	EDGATDA	0.928
129	T	126	132	DGATDAP	0.958
130	D	127	133	GATDAPS	0.979
131	A	128	134	ATDAPST	0.984
132	P	129	135	TDAPSTF	0.988
133	S	130	136	DAPSTFG	0.983
134	T	131	137	APSTFGT	0.989
135	F	132	138	PSTFGTR	0.962
136	G	133	139	STFGTRN	0.921
137	T	134	140	TFGTRNP	0.928
138	R	135	141	FGTRNPN	0.909
139	N	136	142	GTRNPNN	0.864
140	P	137	143	TRNPNND	0.863
141	N	138	144	RNPNNDS	0.878
142	N	139	145	NPNNDSA	0.905
143	D	140	146	PNNDSAI	0.959
144	S	141	147	NNDSAIV	1.004
145	A	142	148	NDSAIVT	1.023
146	I	143	149	DSAIVTQ	1.057
147	V	144	150	SAIVTQF	1.089
148	T	145	151	AIVTQFA	1.097
149	Q	146	152	IVTQFAP	1.097
150	F	147	153	VTQFAPG	1.057
151	A	148	154	TQFAPGT	0.989
152	P	149	155	QFAPGTK	0.992
153	G	150	156	FAPGTKL	1.026
154	T	151	157	APGTKLP	1.022
155	K	152	158	PGTKLPK	1.003
156	L	153	159	GTKLPKN	0.962
157	P	154	160	TKLPKNF	0.993
158	K	155	161	KLPKNFH	1.021
159	N	156	162	LPKNFHI	1.053

Position	Residue	Start	End	Peptide	Score
160	F	157	163	PKNFHIE	0.996
161	H	158	164	KNFHIEG	0.968
162	I	159	165	NFHIEGT	0.965
163	E	160	166	FHIEGTG	0.979
164	G	161	167	HIEGTGG	0.948
165	T	162	168	IEGTGGN	0.901
166	G	163	169	EGTGGNS	0.881
167	G	164	170	GTGGNSQ	0.905
168	N	165	171	TGGNSQS	0.925
169	S	166	172	GGNSQSS	0.939
170	Q	167	173	GNSQSSS	0.959
171	S	168	174	NSQSSSR	0.959
172	S	169	175	SQSSSRA	1.0
173	S	170	176	QSSSRAS	1.0
174	R	171	177	SSSRASS	1.0
175	A	172	178	SSRASSV	1.053
176	S	173	179	SRASSVS	1.053
177	S	174	180	RASSVSR	1.033
178	V	175	181	ASSVSRN	1.019
179	S	176	182	SSVSRNS	1.011
180	R	177	183	SVSRNSS	1.011
181	N	178	184	VSRNSSR	0.992
182	S	179	185	SRNSSRS	0.939
183	S	180	186	RNSSRSS	0.939
184	R	181	187	NSSRSSS	0.958
185	S	182	188	SSRSSSQ	0.993
186	S	183	189	SRSSSQG	0.973
187	S	184	190	RSSSQGS	0.973
188	Q	185	191	SSSQGSR	0.973
189	G	186	192	SSQGSRS	0.973
190	S	187	193	SQGSRSG	0.953
191	R	188	194	QGSRSGN	0.919
192	S	189	195	GSRSGNS	0.919
193	G	190	196	SRSGNST	0.924
194	N	191	197	RSGNSTR	0.904
195	S	192	198	SGNSTRG	0.904
196	T	193	199	GNSTRGT	0.89
197	R	194	200	NSTRGTS	0.909
198	G	195	201	STRGTSP	0.95
199	T	196	202	TRGTSPG	0.931
200	S	197	203	RGTSPGP	0.953

Position	Residue	Start	End	Peptide	Score
201	P	198	204	GTSPGPS	0.973
202	G	199	205	TSPGPSG	0.973
203	P	200	206	SPGPSGI	1.007
204	S	201	207	PGPSGIG	0.988
205	G	202	208	GPSGIGA	0.988
206	I	203	209	PSGIGAV	1.06
207	G	204	210	SGIGAVG	1.033
208	A	205	211	GIGAVGG	1.014
209	V	206	212	IGAVGGD	1.012
210	G	207	213	GAVGGDL	1.026
211	G	208	214	AVGGDLL	1.08
212	D	209	215	VGGDLLY	1.094
213	L	210	216	GGDLLYL	1.075
214	L	211	217	GDLLYLD	1.074
215	Y	212	218	DLLYLDL	1.128
216	L	213	219	LLYLDLL	1.182
217	D	214	220	LYLDLLN	1.115
218	L	215	221	YLDLLNR	1.061
219	L	216	222	LDLLNRL	1.074
220	N	217	223	DLLNRLQ	1.04
221	R	218	224	LLNRLQA	1.068
222	L	219	225	LNRLQAL	1.068
223	Q	220	226	NRLQALE	1.011
224	A	221	227	RLQALES	1.045
225	L	222	228	LQALESG	1.045
226	E	223	229	QALESGK	0.999
227	S	224	230	ALESGKV	1.052
228	G	225	231	LESGKVK	1.033
229	K	226	232	ESGKVKQ	0.999
230	V	227	233	SGKVKQS	1.022
231	K	228	234	GKVKQSQ	1.023
232	Q	229	235	KVKQSQP	1.05
233	S	230	236	VKQSQPK	1.05
234	Q	231	237	KQSQPKV	1.05
235	P	232	238	QSQPKVI	1.082
236	K	233	239	SQPKVIT	1.066
237	V	234	240	QPKVITK	1.055
238	I	235	241	PKVITKK	1.043
239	T	236	242	KVITKKD	1.014
240	K	237	243	VITKKDA	1.033
241	K	238	244	ITKKDAA	0.988
242	D	239	245	TKKDAAA	0.975

Position	Residue	Start	End	Peptide	Score
243	A	240	246	KKDAAAA	0.997
244	A	241	247	KDAAAAK	0.997
245	A	242	248	DAAAAKN	0.975
246	A	243	249	AAAAKNK	0.985
247	K	244	250	AAAKNKM	0.951
248	N	245	251	AAKNKMR	0.923
249	K	246	252	AKNKMRH	0.929
250	M	247	253	KNKMRHK	0.91
251	R	248	254	NKMRHKR	0.902
252	H	249	255	KMRHKRT	0.921
253	K	250	256	MRHKRTS	0.933
254	R	251	257	RHKRTST	0.944
255	T	252	258	HKRTSTK	0.953
256	S	253	259	KRTSTKS	0.939
257	T	254	260	RTSTKSF	0.962
258	K	255	261	TSTKSFN	0.948
259	S	256	262	STKSFNM	0.937
260	F	257	263	TKSFNMV	0.99
261	N	258	264	KSFNMVQ	1.005
262	M	259	265	SFNMVQA	1.024
263	V	260	266	FNMVQAF	1.035
264	Q	261	267	NMVQAFG	1.004
265	A	262	268	MVQAFGL	1.072
266	F	263	269	VQAFGLR	1.079
267	G	264	270	QAFGLRG	1.006
268	L	265	271	AFGLRGP	1.013
269	R	266	272	FGLRGPG	0.986
270	G	267	273	GLRGPGD	0.954
271	P	268	274	LRGPGDL	1.007
272	G	269	275	RGPGDLQ	0.974
273	D	270	276	GPGDLQG	0.974
274	L	271	277	PGDLQGN	0.96
275	Q	272	278	GDLQGNF	0.964
276	G	273	279	DLQGNFG	0.964
277	N	274	280	LQGNFGD	0.964
278	F	275	281	QGNFGDL	0.964
279	G	276	282	GNFGDLQ	0.964
280	D	277	283	NFGDLQL	1.017
281	L	278	284	FGDLQLN	1.017
282	Q	279	285	GDLQLNK	0.994
283	L	280	286	DLQLNKL	1.048

Position	Residue	Start	End	Peptide	Score
284	N	281	287	LQLNKLG	1.049
285	K	282	288	QLNKLGT	1.001
286	L	283	289	LNKLGTE	0.977
287	G	284	290	NKLGTED	0.922
288	T	285	291	KLGTEDP	0.963
289	E	286	292	LGTEDPR	0.955
290	D	287	293	GTEDPRW	0.904
291	P	288	294	TEDPRWP	0.931
292	R	289	295	EDPRWPQ	0.947
293	W	290	296	DPRWPQI	0.99
294	P	291	297	PRWPQIA	1.018
295	Q	292	298	RWPQIAE	0.987
296	I	293	299	WPQIAEL	1.041
297	A	294	300	PQIAELA	1.066
298	E	295	301	QIAELAP	1.066
299	L	296	302	IAELAPT	1.051
300	A	297	303	AELAPTA	1.038
301	P	298	304	ELAPTAS	1.031
302	T	299	305	LAPTASA	1.061
303	A	300	306	APTASAF	1.038
304	S	301	307	PTASAFM	1.004
305	A	302	308	TASAFMG	0.977
306	F	303	309	ASAFMGM	0.965
307	M	304	310	SAFMGMS	0.958
308	G	305	311	AFMGMSQ	0.958
309	M	306	312	FMGMSQF	0.962
310	S	307	313	MGMSQFK	0.939
311	Q	308	314	GMSQFKL	1.0
312	F	309	315	MSQFKLT	1.005
313	K	310	316	SQFKLTH	1.045
314	L	311	317	QFKLTHQ	1.045
315	T	312	318	FKLTHQN	1.011
316	H	313	319	KLTHQNN	0.966
317	Q	314	320	LTHQNND	0.957
318	N	315	321	THQNNDD	0.902
319	N	316	322	HQNNDDH	0.93
320	D	317	323	QNNDDHG	0.897
321	D	318	324	NNDDHGN	0.863
322	H	319	325	NDDHGNP	0.904
323	G	320	326	DDHGNPV	0.991
324	N	321	327	DHGNPVY	1.033
325	P	322	328	HGNPVYF	1.065

Position	Residue	Start	End	Peptide	Score
326	V	323	329	GNPVYFL	1.086
327	Y	324	330	NPVYFLR	1.085
328	F	325	331	PVYFLRY	1.14
329	L	326	332	VYFLRYS	1.133
330	R	327	333	YFLRYSG	1.06
331	Y	328	334	FLRYSGA	1.046
332	S	329	335	LRYSGAI	1.055
333	G	330	336	RYSGAIK	1.009
334	A	331	337	YSGAIKL	1.063
335	I	332	338	SGAIKLD	1.021
336	K	333	339	GAIKLDP	1.029
337	L	334	340	AIKLDPK	1.037
338	D	335	341	IKLDPKN	0.995
339	P	336	342	KLDPKNP	0.983
340	K	337	343	LDPKNPN	0.961
341	N	338	344	DPKNPNY	0.948
342	P	339	345	PKNPNYN	0.935
343	N	340	346	KNPNYNK	0.916
344	Y	341	347	NPNYNKW	0.911
345	N	342	348	PNYNKWL	0.979
346	K	343	349	NYNKWLE	0.948
347	W	344	350	YNKWLEL	1.016
348	L	345	351	NKWLELL	1.029
349	E	346	352	KWLELLE	1.039
350	L	347	353	WLELLEQ	1.051
351	L	348	354	LELLEQN	1.035
352	E	349	355	ELLEQNI	1.021
353	Q	350	356	LLEQNID	1.023
354	N	351	357	LEQNIDA	0.996
355	I	352	358	EQNIDAY	0.984
356	D	353	359	QNIDAYK	0.995
357	A	354	360	NIDAYKT	0.98
358	Y	355	361	IDAYKTF	1.025
359	K	356	362	DAYKTFP	1.012
360	T	357	363	AYKTFPK	1.021
361	F	358	364	YKTFPKK	1.002
362	P	359	365	KTFPKKE	0.958
363	K	360	366	TFPKKEK	0.958
364	K	361	367	FPKKEKK	0.961
365	E	362	368	PKKEKKQ	0.95
366	K	363	369	KKEKKQK	0.931

Position	Residue	Start	End	Peptide	Score
367	K	364	370	KEKKQKA	0.95
368	Q	365	371	EKKQKAP	0.969
369	K	366	372	KKQKAPK	0.98
370	A	367	373	KQKAPKE	0.969
371	P	368	374	QKAPKEE	0.958
372	K	369	375	KAPKEES	0.957
373	E	370	376	APKEEST	0.954
374	E	371	377	PKEESTD	0.926
375	S	372	378	KEESTDQ	0.919
376	T	373	379	EESTDQM	0.904
377	D	374	380	ESTDQMS	0.927
378	Q	375	381	STDQMSE	0.927
379	M	376	382	TDQMSEP	0.935
380	S	377	383	DQMSEPP	0.957
381	E	378	384	QMSEPPK	0.966
382	P	379	385	MSEPPKE	0.943
383	P	380	386	SEPPKEQ	0.97
384	K	381	387	EPPKEQR	0.95
385	E	382	388	PPKEQRV	1.026
386	Q	383	389	PKEQRVQ	1.019
387	R	384	390	KEQRVQG	0.992
388	V	385	391	EQRVQGS	1.003
389	Q	386	392	QRVQGSI	1.046
390	G	387	393	RVQGSIT	1.031
391	S	388	394	VQGSITQ	1.051
392	I	389	395	QGSITQR	0.979
393	T	390	396	GSITQRT	0.963
394	Q	391	397	SITQRTR	0.963
395	R	392	398	ITQRTRT	0.949
396	T	393	399	TQRTRTR	0.909
397	R	394	400	QRTRTRP	0.931
398	T	395	401	RTRTRPS	0.93
399	R	396	402	TRTRPSV	1.003
400	P	397	403	RTRPSVQ	1.018
401	S	398	404	TRPSVQP	1.046
402	V	399	405	RPSVQPG	1.041
403	Q	400	406	PSVQPGP	1.068
404	P	401	407	SVQPGPM	1.034
405	G	402	408	VQPGPMI	1.054
406	P	403	409	QPGPMID	0.98
407	M	404	410	PGPMIDV	1.033
408	I	405	411	GPMIDVN	0.992

Position	Residue	Start	End	Peptide	Score
409	D	406	412	PMIDVNT	0.997
410	V	407	413	MIDVNTD	0.968

Download result

© 2005-2015 | IEDB Home
Supported by a contract from the National Institute of Allergy and Infectious Diseases, a component of the National Institutes of Health in the Department of Health and Human Services.
