# Supplementary material for: Epitope-Based Vaccine Target Screening against Highly Pathogenic MERS-CoV: An In Silico Approach Applied to Emerging Infectious Diseases
Source: PLoS One. 2015 Dec 7;10(12):e0144475. doi: 10.1371/journal.pone.0144475 (PMC4671582; doi:10.1371/journal.pone.0144475)
Supplement: S2 Fig — X-axis represents the amino acids, whereas Y-axis represents accessible propensity. Average antigenic score was 1.000. Area displayed by the threshold line is considered as potential B-cell accessible regions. (PDF) [file pone.0144475.s002.pdf]

# IEDB Analysis Resource

Home | Help | Example | Reference | Download | Contact

## Emini Surface Accessibility Prediction Prediction

### Input Sequences

```
  1 MASPAAPRAV SFADNNDITN TNLSRGRGRN PKPRAAPNNT VSWYTGLTQH GKVPLTFPPG
 61 QGVPLNANST PAQNAGYWRR QDRKINTGNG IKQLAPRWYF YYTGTGPEAA LPFRAVKDGI
121 VWVHEDGATD APSTFGTRNP NNDSAIVTQF APGTKLPKNF HIEGTGGNSQ SSSRASSVSR
181 NSSRSSSQGS RSGNSTRGTS PGPSGIGAVG GDLLYLDLLN RLQALESGKV KQSQPKVITK
241 KDAAAAKNKM RHKRTSTKSF NMVQAFGLRG PGDLQGNFGD LQLNKLGTED PRWPQIAELA
301 PTASAFMGMS QFKLTHQNND DHGNPVYFLR YSGAIKLDPK NPNYNKWLEL LEQNIDAYKT
361 FPKKEKKQKA PKEESTDQMS EPPKEQRVQG SITQRTRTRP SVQPGPMIDV NTD
```

**Center position:** 3 **Window size:** 6 **Threshold:** 1.000 Recalculate

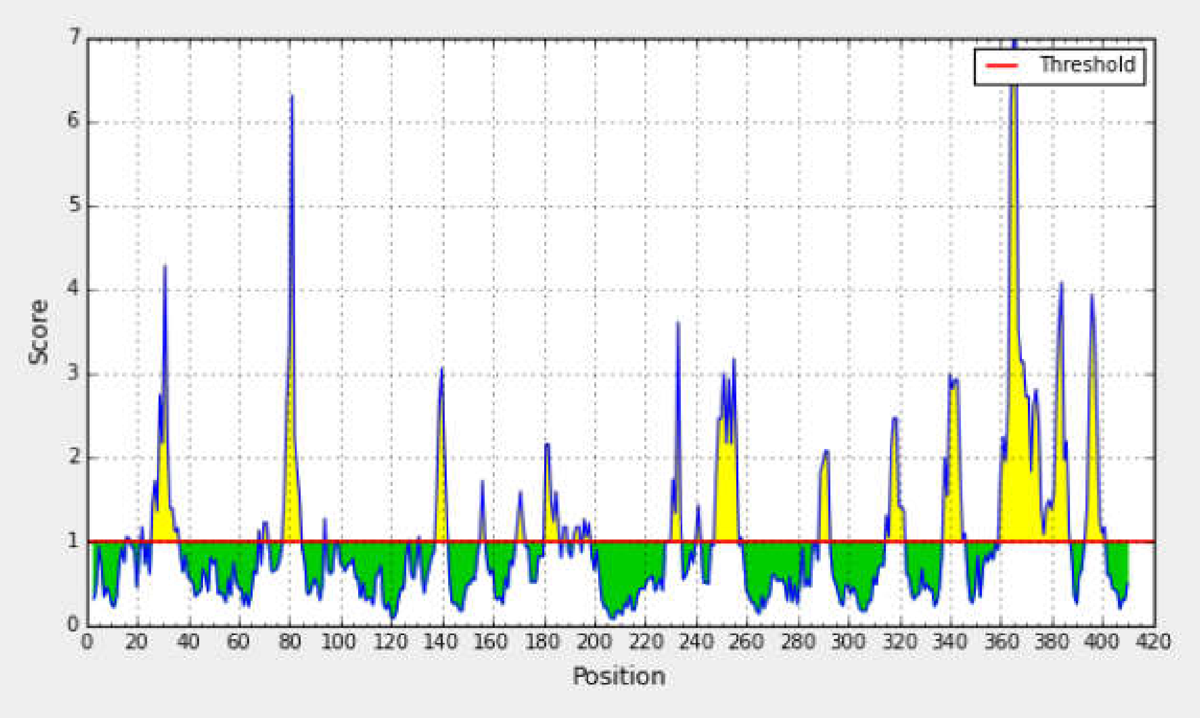


**Average:** 1.000 **Minimum:** 0.074 **Maximum:** 6.971

### Predicted residue scores:

| Position | Residue | Start | End | Peptide | Score |
|---|---|---|---|---|---|
| 3 | S | 1 | 6 | MASPAA | 0.307 |
| 4 | P | 2 | 7 | ASPAAP | 0.48 |
| 5 | A | 3 | 8 | SPAAPR | 0.931 |
| 6 | A | 4 | 9 | PAAPRA | 0.702 |
| 7 | P | 5 | 10 | AAPRAV | 0.337 |
| 8 | R | 6 | 11 | APRAVS | 0.447 |
| 9 | A | 7 | 12 | PRAVSF | 0.383 |
| 10 | V | 8 | 13 | RAVSFA | 0.25 |

| Position | Residue | Start | End | Peptide | Score |
|---|---|---|---|---|---|
| 11 | S | 9 | 14 | AVSFAD | 0.213 |
| 12 | F | 10 | 15 | VSFADN | 0.34 |
| 13 | A | 11 | 16 | SFADNN | 0.736 |
| 14 | D | 12 | 17 | FADNND | 0.917 |
| 15 | N | 13 | 18 | ADNNDI | 0.742 |
| 16 | N | 14 | 19 | DNNDIT | 1.06 |
| 17 | D | 15 | 20 | NNDITN | 1.021 |
| 18 | I | 16 | 21 | NDITNT | 0.916 |
| 19 | T | 17 | 22 | DITNTN | 0.916 |
| 20 | N | 18 | 23 | ITNTNL | 0.452 |
| 21 | T | 19 | 24 | TNTNLS | 0.865 |
| 22 | N | 20 | 25 | NTNLSR | 1.174 |
| 23 | L | 21 | 26 | TNLSRG | 0.722 |
| 24 | S | 22 | 27 | NLSRGR | 0.98 |
| 25 | R | 23 | 28 | LSRGRG | 0.603 |
| 26 | G | 24 | 29 | SRGRGR | 1.433 |
| 27 | R | 25 | 30 | RGRGRN | 1.72 |
| 28 | G | 26 | 31 | GRGRNP | 1.358 |
| 29 | R | 27 | 32 | RGRNPK | 2.743 |
| 30 | N | 28 | 33 | GRNPKP | 2.166 |
| 31 | P | 29 | 34 | RNPKPR | 4.286 |
| 32 | K | 30 | 35 | NPKPRA | 2.211 |
| 33 | P | 31 | 36 | PKPRAA | 1.389 |
| 34 | R | 32 | 37 | KPRAAP | 1.389 |
| 35 | A | 33 | 38 | PRAAPN | 1.117 |
| 36 | A | 34 | 39 | RAAPNN | 1.162 |
| 37 | P | 35 | 40 | AAPNNT | 0.856 |
| 38 | N | 36 | 41 | APNNTV | 0.629 |
| 39 | N | 37 | 42 | PNNTVS | 0.834 |
| 40 | T | 38 | 43 | NNTVSW | 0.567 |
| 41 | V | 39 | 44 | NTVSWY | 0.553 |
| 42 | S | 40 | 45 | TVSWYT | 0.496 |
| 43 | W | 41 | 46 | VSWYTG | 0.34 |
| 44 | Y | 42 | 47 | SWYTGL | 0.378 |
| 45 | T | 43 | 48 | WYTGLT | 0.407 |
| 46 | G | 44 | 49 | YTGLTQ | 0.67 |
| 47 | L | 45 | 50 | TGLTQH | 0.582 |
| 48 | T | 46 | 51 | GLTQHG | 0.399 |
| 49 | Q | 47 | 52 | LTQHGK | 0.807 |
| 50 | H | 48 | 53 | TQHGKV | 0.726 |
| 51 | G | 49 | 54 | QHGKVP | 0.778 |

| Position | Residue | Start | End | Peptide | Score |
|---|---|---|---|---|---|
| 52 | K | 50 | 55 | HGKVPL | 0.37 |
| 53 | V | 51 | 56 | GKVPLT | 0.393 |
| 54 | P | 52 | 57 | KVPLTF | 0.344 |
| 55 | L | 53 | 58 | VPLTFP | 0.266 |
| 56 | T | 54 | 59 | PLTFPP | 0.554 |
| 57 | F | 55 | 60 | LTFPPG | 0.354 |
| 58 | P | 56 | 61 | TFPPGQ | 0.744 |
| 59 | P | 57 | 62 | FPPGQG | 0.51 |
| 60 | G | 58 | 63 | PPGQGV | 0.437 |
| 61 | Q | 59 | 64 | PGQGVP | 0.437 |
| 62 | G | 60 | 65 | GQGVPL | 0.233 |
| 63 | V | 61 | 66 | QGVPLN | 0.379 |
| 64 | P | 62 | 67 | GVPLNA | 0.221 |
| 65 | L | 63 | 68 | VPLNAN | 0.359 |
| 66 | N | 64 | 69 | PLNANS | 0.649 |
| 67 | A | 65 | 70 | LNANST | 0.606 |
| 68 | N | 66 | 71 | NANSTP | 1.135 |
| 69 | S | 67 | 72 | ANSTPA | 0.713 |
| 70 | T | 68 | 73 | NSTPAQ | 1.223 |
| 71 | P | 69 | 74 | STPAQN | 1.223 |
| 72 | A | 70 | 75 | TPAQNA | 0.922 |
| 73 | Q | 71 | 76 | PAQNAG | 0.632 |
| 74 | N | 72 | 77 | AQNAGY | 0.64 |
| 75 | A | 73 | 78 | QNAGYW | 0.667 |
| 76 | G | 74 | 79 | NAGYWR | 0.754 |
| 77 | Y | 75 | 80 | AGYWRR | 0.918 |
| 78 | W | 76 | 81 | GYWRRQ | 1.574 |
| 79 | R | 77 | 82 | YWRRQD | 2.656 |
| 80 | R | 78 | 83 | WRRQDR | 3.32 |
| 81 | Q | 79 | 84 | RRQDRK | 6.315 |
| 82 | D | 80 | 85 | RQDRKI | 2.26 |
| 83 | R | 81 | 86 | QDRKIN | 1.856 |
| 84 | K | 82 | 87 | DRKINT | 1.546 |
| 85 | I | 83 | 88 | RKINTG | 0.916 |
| 86 | N | 84 | 89 | KINTGN | 0.752 |
| 87 | T | 85 | 90 | INTGNG | 0.372 |
| 88 | G | 86 | 91 | NTGNGI | 0.372 |
| 89 | N | 87 | 92 | TGNGIK | 0.463 |
| 90 | G | 88 | 93 | GNGIKQ | 0.556 |
| 91 | I | 89 | 94 | NGIKQL | 0.463 |
| 92 | K | 90 | 95 | GIKQLA | 0.291 |
| 93 | Q | 91 | 96 | IKQLAP | 0.454 |

| Position | Residue | Start | End | Peptide | Score |
|---|---|---|---|---|---|
| 94 | L | 92 | 97 | KQLAPR | 1.27 |
| 95 | A | 93 | 98 | QLAPRW | 0.668 |
| 96 | P | 94 | 99 | LAPRWY | 0.604 |
| 97 | R | 95 | 100 | APRWYF | 0.634 |
| 98 | W | 96 | 101 | PRWYFY | 0.984 |
| 99 | Y | 97 | 102 | RWYFYY | 0.997 |
| 100 | F | 98 | 103 | WYFYYT | 0.735 |
| 101 | Y | 99 | 104 | YFYYTG | 0.691 |
| 102 | Y | 100 | 105 | FYYTGT | 0.637 |
| 103 | T | 101 | 106 | YYTGTG | 0.728 |
| 104 | G | 102 | 107 | YTGTGP | 0.718 |
| 105 | T | 103 | 108 | TGTGPE | 0.794 |
| 106 | G | 104 | 109 | GTGPEA | 0.556 |
| 107 | P | 105 | 110 | TGPEAA | 0.567 |
| 108 | E | 106 | 111 | GPEAAL | 0.324 |
| 109 | A | 107 | 112 | PEAALP | 0.506 |
| 110 | A | 108 | 113 | EAALPF | 0.284 |
| 111 | L | 109 | 114 | AALPFR | 0.321 |
| 112 | P | 110 | 115 | ALPFRA | 0.321 |
| 113 | F | 111 | 116 | LPFRAV | 0.236 |
| 114 | R | 112 | 117 | PFRAVK | 0.571 |
| 115 | A | 113 | 118 | FRAVKD | 0.617 |
| 116 | V | 114 | 119 | RAVKDG | 0.705 |
| 117 | K | 115 | 120 | AVKDGI | 0.252 |
| 118 | D | 116 | 121 | VKDGIV | 0.185 |
| 119 | G | 117 | 122 | KDGIVW | 0.263 |
| 120 | I | 118 | 123 | DGIVWV | 0.098 |
| 121 | V | 119 | 124 | GIVWVH | 0.079 |
| 122 | W | 120 | 125 | IVWVHE | 0.139 |
| 123 | V | 121 | 126 | VWVHED | 0.331 |
| 124 | H | 122 | 127 | WVHEDG | 0.442 |
| 125 | E | 123 | 128 | VHEDGA | 0.424 |
| 126 | D | 124 | 129 | HEDGAT | 0.825 |
| 127 | G | 125 | 130 | EDGATD | 1.013 |
| 128 | A | 126 | 131 | DGATDA | 0.591 |
| 129 | T | 127 | 132 | GATDAP | 0.547 |
| 130 | D | 128 | 133 | ATDAPS | 0.741 |
| 131 | A | 129 | 134 | TDAPST | 1.058 |
| 132 | P | 130 | 135 | DAPSTF | 0.635 |
| 133 | S | 131 | 136 | APSTFG | 0.376 |
| 134 | T | 132 | 137 | PSTFGT | 0.537 |

| Position | Residue | Start | End | Peptide | Score |
|---|---|---|---|---|---|
| 135 | F | 133 | 138 | STFGTR | 0.681 |
| 136 | G | 134 | 139 | TFGTRN | 0.817 |
| 137 | T | 135 | 140 | FGTRNP | 0.875 |
| 138 | R | 136 | 141 | GTRNPN | 1.625 |
| 139 | N | 137 | 142 | TRNPNN | 2.641 |
| 140 | P | 138 | 143 | RNPNND | 3.056 |
| 141 | N | 139 | 144 | NPNNDS | 2.091 |
| 142 | N | 140 | 145 | PNNDSA | 1.314 |
| 143 | D | 141 | 146 | NNDSAI | 0.596 |
| 144 | S | 142 | 147 | NDSAIV | 0.275 |
| 145 | A | 143 | 148 | DSAIVT | 0.247 |
| 146 | I | 144 | 149 | SAIVTQ | 0.256 |
| 147 | V | 145 | 150 | AIVTQF | 0.165 |
| 148 | T | 146 | 151 | IVTQFA | 0.165 |
| 149 | Q | 147 | 152 | VTQFAP | 0.365 |
| 150 | F | 148 | 153 | TQFAPG | 0.486 |
| 151 | A | 149 | 154 | QFAPGT | 0.486 |
| 152 | P | 150 | 155 | FAPGTK | 0.561 |
| 153 | G | 151 | 156 | APGTKL | 0.535 |
| 154 | T | 152 | 157 | PGTKLP | 0.818 |
| 155 | K | 153 | 158 | GTKLPK | 1.058 |
| 156 | L | 154 | 159 | TKLPKN | 1.72 |
| 157 | P | 155 | 160 | KLPKNF | 1.032 |
| 158 | K | 156 | 161 | LPKNFH | 0.702 |
| 159 | N | 157 | 162 | PKNFHI | 0.597 |
| 160 | F | 158 | 163 | KNFHIE | 0.668 |
| 161 | H | 159 | 164 | NFHIEG | 0.331 |
| 162 | I | 160 | 165 | FHIEGT | 0.297 |
| 163 | E | 161 | 166 | HIEGTG | 0.339 |
| 164 | G | 162 | 167 | IEGTGG | 0.247 |
| 165 | T | 163 | 168 | EGTGGN | 0.566 |
| 166 | G | 164 | 169 | GTGGNS | 0.438 |
| 167 | G | 165 | 170 | TGGNSQ | 0.767 |
| 168 | N | 166 | 171 | GGNSQS | 0.712 |
| 169 | S | 167 | 172 | GNSQSS | 0.964 |
| 170 | Q | 168 | 173 | NSQSSS | 1.305 |
| 171 | S | 169 | 174 | SQSSSR | 1.59 |
| 172 | S | 170 | 175 | QSSSRA | 1.198 |
| 173 | S | 171 | 176 | SSSRAS | 0.927 |
| 174 | R | 172 | 177 | SSRASS | 0.927 |
| 175 | A | 173 | 178 | SRASSV | 0.514 |
| 176 | S | 174 | 179 | RASSVS | 0.514 |

| Position | Residue | Start | End | Peptide | Score |
|---|---|---|---|---|---|
| 177 | S | 175 | 180 | ASSVSR | 0.514 |
| 178 | V | 176 | 181 | SSVSRN | 0.818 |
| 179 | S | 177 | 182 | SVSRNS | 0.818 |
| 180 | R | 178 | 183 | VSRNSS | 0.818 |
| 181 | N | 179 | 184 | SRNSSR | 2.157 |
| 182 | S | 180 | 185 | RNSSRS | 2.157 |
| 183 | S | 181 | 186 | NSSRSS | 1.476 |
| 184 | R | 182 | 187 | SSRSSS | 1.23 |
| 185 | S | 183 | 188 | SRSSSQ | 1.59 |
| 186 | S | 184 | 189 | RSSSQG | 1.174 |
| 187 | S | 185 | 190 | SSSQGS | 0.803 |
| 188 | Q | 186 | 191 | SSQGSR | 1.174 |
| 189 | G | 187 | 192 | SQGSRS | 1.174 |
| 190 | S | 188 | 193 | QGSRSG | 0.867 |
| 191 | R | 189 | 194 | GSRSGN | 0.805 |
| 192 | S | 190 | 195 | SRSGNS | 1.09 |
| 193 | G | 191 | 196 | RSGNST | 1.174 |
| 194 | N | 192 | 197 | SGNSTR | 1.174 |
| 195 | S | 193 | 198 | GNSTRG | 0.867 |
| 196 | T | 194 | 199 | NSTRGT | 1.264 |
| 197 | R | 195 | 200 | STRGTS | 1.054 |
| 198 | G | 196 | 201 | TRGTSP | 1.216 |
| 199 | T | 197 | 202 | RGTSPG | 0.834 |
| 200 | S | 198 | 203 | GTSPGP | 0.658 |
| 201 | P | 199 | 204 | TSPGPS | 0.891 |
| 202 | G | 200 | 205 | SPGPSG | 0.611 |
| 203 | P | 201 | 206 | PGPSGI | 0.32 |
| 204 | S | 202 | 207 | GPSGIG | 0.205 |
| 205 | G | 203 | 208 | PSGIGA | 0.209 |
| 206 | I | 204 | 209 | SGIGAV | 0.1 |
| 207 | G | 205 | 210 | GIGAVG | 0.074 |
| 208 | A | 206 | 211 | IGAVGG | 0.074 |
| 209 | V | 207 | 212 | GAVGGD | 0.176 |
| 210 | G | 208 | 213 | AVGGDL | 0.147 |
| 211 | G | 209 | 214 | VGGDLL | 0.12 |
| 212 | D | 210 | 215 | GGDLLY | 0.253 |
| 213 | L | 211 | 216 | GDLLYL | 0.211 |
| 214 | L | 212 | 217 | DLLYLD | 0.356 |
| 215 | Y | 213 | 218 | LLYLDL | 0.176 |
| 216 | L | 214 | 219 | LYLDLL | 0.176 |
| 217 | D | 215 | 220 | YLDLLN | 0.343 |

| Position | Residue | Start | End | Peptide | Score |
|---|---|---|---|---|---|
| 218 | L | 216 | 221 | LDLLNR | 0.429 |
| 219 | L | 217 | 222 | DLLNRL | 0.429 |
| 220 | N | 218 | 223 | LLNRLQ | 0.445 |
| 221 | R | 219 | 224 | LNRLQA | 0.545 |
| 222 | L | 220 | 225 | NRLQAL | 0.545 |
| 223 | Q | 221 | 226 | RLQALE | 0.586 |
| 224 | A | 222 | 227 | LQALES | 0.401 |
| 225 | L | 223 | 228 | QALESG | 0.482 |
| 226 | E | 224 | 229 | ALESGK | 0.556 |
| 227 | S | 225 | 230 | LESGKV | 0.409 |
| 228 | G | 226 | 231 | ESGKVK | 0.991 |
| 229 | K | 227 | 232 | SGKVKQ | 0.991 |
| 230 | V | 228 | 233 | GKVKQS | 0.991 |
| 231 | K | 229 | 234 | KVKQSQ | 1.734 |
| 232 | Q | 230 | 235 | VKQSQP | 1.34 |
| 233 | S | 231 | 236 | KQSQPK | 3.612 |
| 234 | Q | 232 | 237 | QSQPKV | 1.34 |
| 235 | P | 233 | 238 | SQPKVI | 0.543 |
| 236 | K | 234 | 239 | QPKVIT | 0.584 |
| 237 | V | 235 | 240 | PKVITK | 0.675 |
| 238 | I | 236 | 241 | KVITKK | 0.873 |
| 239 | T | 237 | 242 | VITKKD | 0.729 |
| 240 | K | 238 | 243 | ITKKDA | 0.992 |
| 241 | K | 239 | 244 | TKKDAA | 1.429 |
| 242 | D | 240 | 245 | KKDAAA | 1.001 |
| 243 | A | 241 | 246 | KDAAAA | 0.505 |
| 244 | A | 242 | 247 | DAAAAK | 0.505 |
| 245 | A | 243 | 248 | AAAAKN | 0.487 |
| 246 | A | 244 | 249 | AAAKNK | 0.964 |
| 247 | K | 245 | 250 | AAKNKM | 0.944 |
| 248 | N | 246 | 251 | AKNKMR | 1.83 |
| 249 | K | 247 | 252 | KNKMRH | 2.465 |
| 250 | M | 248 | 253 | NKMRHK | 2.465 |
| 251 | R | 249 | 254 | KMRHKR | 3.002 |
| 252 | H | 250 | 255 | MRHKRT | 2.167 |
| 253 | K | 251 | 256 | RHKRTS | 2.934 |
| 254 | R | 252 | 257 | HKRTST | 2.162 |
| 255 | T | 253 | 258 | KRTSTK | 3.177 |
| 256 | S | 254 | 259 | RTSTKS | 2.129 |
| 257 | T | 255 | 260 | TSTKSF | 0.941 |
| 258 | K | 256 | 261 | STKSFN | 1.049 |
| 259 | S | 257 | 262 | TKSFNM | 0.775 |

| Position | Residue | Start | End | Peptide | Score |
|---|---|---|---|---|---|
| 260 | F | 258 | 263 | KSFNMV | 0.398 |
| 261 | N | 259 | 264 | SFNMVQ | 0.345 |
| 262 | M | 260 | 265 | FNMVQA | 0.26 |
| 263 | V | 261 | 266 | NMVQAF | 0.26 |
| 264 | Q | 262 | 267 | MVQAFG | 0.16 |
| 265 | A | 263 | 268 | VQAFGL | 0.133 |
| 266 | F | 264 | 269 | QAFGLR | 0.352 |
| 267 | G | 265 | 270 | AFGLRG | 0.201 |
| 268 | L | 266 | 271 | FGLRGP | 0.308 |
| 269 | R | 267 | 272 | GLRGPG | 0.352 |
| 270 | G | 268 | 273 | LRGPGD | 0.594 |
| 271 | P | 269 | 274 | RGPGDL | 0.594 |
| 272 | G | 270 | 275 | GPGDLQ | 0.525 |
| 273 | D | 271 | 276 | PGDLQG | 0.525 |
| 274 | L | 272 | 277 | GDLQGN | 0.546 |
| 275 | Q | 273 | 278 | DLQGNF | 0.478 |
| 276 | G | 274 | 279 | LQGNFG | 0.283 |
| 277 | N | 275 | 280 | QGNFGD | 0.573 |
| 278 | F | 276 | 281 | GNFGDL | 0.273 |
| 279 | G | 277 | 282 | NFGDLQ | 0.478 |
| 280 | D | 278 | 283 | FGDLQL | 0.245 |
| 281 | L | 279 | 284 | GDLQLN | 0.455 |
| 282 | Q | 280 | 285 | DLQLNK | 0.919 |
| 283 | L | 281 | 286 | LQLNKL | 0.454 |
| 284 | N | 282 | 287 | QLNKLG | 0.545 |
| 285 | K | 283 | 288 | LNKLGT | 0.454 |
| 286 | L | 284 | 289 | NKLGTE | 0.953 |
| 287 | G | 285 | 290 | KLGTED | 0.99 |
| 288 | T | 286 | 291 | LGTEDP | 0.765 |
| 289 | E | 287 | 292 | GTEDPR | 1.818 |
| 290 | D | 288 | 293 | TEDPRW | 1.931 |
| 291 | P | 289 | 294 | EDPRWP | 2.069 |
| 292 | R | 290 | 295 | DPRWPQ | 2.069 |
| 293 | W | 291 | 296 | PRWPQI | 0.869 |
| 294 | P | 292 | 297 | RWPQIA | 0.568 |
| 295 | Q | 293 | 298 | WPQIAE | 0.502 |
| 296 | I | 294 | 299 | PQIAEL | 0.394 |
| 297 | A | 295 | 300 | QIAELA | 0.257 |
| 298 | E | 296 | 301 | IAELAP | 0.23 |
| 299 | L | 297 | 302 | AELAPT | 0.473 |
| 300 | A | 298 | 303 | ELAPTA | 0.473 |

| Position | Residue | Start | End | Peptide | Score |
|---|---|---|---|---|---|
| 301 | P | 299 | 304 | LAPTAS | 0.366 |
| 302 | T | 300 | 305 | APTASA | 0.448 |
| 303 | A | 301 | 306 | PTASAF | 0.384 |
| 304 | S | 302 | 307 | TASAFM | 0.246 |
| 305 | A | 303 | 308 | ASAFMG | 0.169 |
| 306 | F | 304 | 309 | SAFMGM | 0.165 |
| 307 | M | 305 | 310 | AFMGMS | 0.165 |
| 308 | G | 306 | 311 | FMGMSQ | 0.283 |
| 309 | M | 307 | 312 | MGMSQF | 0.283 |
| 310 | S | 308 | 313 | GMSQFK | 0.572 |
| 311 | Q | 309 | 314 | MSQFKL | 0.477 |
| 312 | F | 310 | 315 | SQFKLT | 0.695 |
| 313 | K | 311 | 316 | QFKLTH | 0.706 |
| 314 | L | 312 | 317 | FKLTHQ | 0.706 |
| 315 | T | 313 | 318 | KLTHQN | 1.311 |
| 316 | H | 314 | 319 | LTHQNN | 1.054 |
| 317 | Q | 315 | 320 | THQNND | 2.134 |
| 318 | N | 316 | 321 | HQNNDD | 2.47 |
| 319 | N | 317 | 322 | QNNDDH | 2.47 |
| 320 | D | 318 | 323 | NNDDHG | 1.411 |
| 321 | D | 319 | 324 | NDDHGN | 1.411 |
| 322 | H | 320 | 325 | DDHGNP | 1.357 |
| 323 | G | 321 | 326 | DHGNPV | 0.603 |
| 324 | N | 322 | 327 | HGNPVY | 0.566 |
| 325 | P | 323 | 328 | GNPVYF | 0.36 |
| 326 | V | 324 | 329 | NPVYFL | 0.3 |
| 327 | Y | 325 | 330 | PVYFLR | 0.365 |
| 328 | F | 326 | 331 | VYFLRY | 0.37 |
| 329 | L | 327 | 332 | YFLRYS | 0.669 |
| 330 | R | 328 | 333 | FLRYSG | 0.422 |
| 331 | Y | 329 | 334 | LRYSGA | 0.493 |
| 332 | S | 330 | 335 | RYSGAI | 0.419 |
| 333 | G | 331 | 336 | YSGAIK | 0.428 |
| 334 | A | 332 | 337 | SGAIKL | 0.225 |
| 335 | I | 333 | 338 | GAIKLD | 0.28 |
| 336 | K | 334 | 339 | AIKLDP | 0.438 |
| 337 | L | 335 | 340 | IKLDPK | 0.868 |
| 338 | D | 336 | 341 | KLDPKN | 1.99 |
| 339 | P | 337 | 342 | LDPKNP | 1.539 |
| 340 | K | 338 | 343 | DPKNPN | 3.001 |
| 341 | N | 339 | 344 | PKNPNY | 2.815 |
| 342 | P | 340 | 345 | KNPNYN | 2.928 |

| Position | Residue | Start | End | Peptide | Score |
|---|---|---|---|---|---|
| 343 | N | 341 | 346 | NPNYNK | 2.928 |
| 344 | Y | 342 | 347 | PNYNKW | 1.915 |
| 345 | N | 343 | 348 | NYNKWL | 1.021 |
| 346 | K | 344 | 349 | YNKWLE | 1.1 |
| 347 | W | 345 | 350 | NKWLEL | 0.579 |
| 348 | L | 346 | 351 | KWLELL | 0.297 |
| 349 | E | 347 | 352 | WLELLE | 0.257 |
| 350 | L | 348 | 353 | LELLEQ | 0.423 |
| 351 | L | 349 | 354 | ELLEQN | 0.825 |
| 352 | E | 350 | 355 | LLEQNI | 0.334 |
| 353 | Q | 351 | 356 | LEQNID | 0.677 |
| 354 | N | 352 | 357 | EQNIDA | 0.829 |
| 355 | I | 353 | 358 | QNIDAY | 0.75 |
| 356 | D | 354 | 359 | NIDAYK | 0.866 |
| 357 | A | 355 | 360 | IDAYKT | 0.777 |
| 358 | Y | 356 | 361 | DAYKTF | 0.96 |
| 359 | K | 357 | 362 | AYKTFP | 0.889 |
| 360 | T | 358 | 363 | YKTFPK | 1.76 |
| 361 | F | 359 | 364 | KTFPKK | 2.246 |
| 362 | P | 360 | 365 | TFPKKE | 1.945 |
| 363 | K | 361 | 366 | FPKKEK | 2.695 |
| 364 | K | 362 | 367 | PKKEKK | 6.224 |
| 365 | E | 363 | 368 | KKEKKQ | 6.971 |
| 366 | K | 364 | 369 | KEKKQK | 6.971 |
| 367 | K | 365 | 370 | EKKQKA | 3.522 |
| 368 | Q | 366 | 371 | KKQKAP | 3.144 |
| 369 | K | 367 | 372 | KQKAPK | 3.144 |
| 370 | A | 368 | 373 | QKAPKE | 2.723 |
| 371 | P | 369 | 374 | KAPKEE | 2.723 |
| 372 | K | 370 | 375 | APKEES | 1.825 |
| 373 | E | 371 | 376 | PKEEST | 2.607 |
| 374 | E | 372 | 377 | KEESTD | 2.815 |
| 375 | S | 373 | 378 | EESTDQ | 2.438 |
| 376 | T | 374 | 379 | ESTDQM | 1.393 |
| 377 | D | 375 | 380 | STDQMS | 1.078 |
| 378 | Q | 376 | 381 | TDQMSE | 1.393 |
| 379 | M | 377 | 382 | DQMSEP | 1.493 |
| 380 | S | 378 | 383 | QMSEPP | 1.382 |
| 381 | E | 379 | 384 | MSEPPK | 1.596 |
| 382 | P | 380 | 385 | SEPPKE | 2.793 |
| 383 | P | 381 | 386 | EPPKEQ | 3.609 |

| Position | Residue | Start | End | Peptide | Score |
|---|---|---|---|---|---|
| 384 | K | 382 | 387 | PPKEQR | 4.082 |
| 385 | E | 383 | 388 | PKEQRV | 1.959 |
| 386 | Q | 384 | 389 | KEQRVQ | 2.194 |
| 387 | R | 385 | 390 | EQRVQG | 1.086 |
| 388 | V | 386 | 391 | QRVQGS | 0.84 |
| 389 | Q | 387 | 392 | RVQGSI | 0.34 |
| 390 | G | 388 | 393 | VQGSIT | 0.251 |
| 391 | S | 389 | 394 | QGSITQ | 0.585 |
| 392 | I | 390 | 395 | GSITQR | 0.661 |
| 393 | T | 391 | 396 | SITQRT | 0.964 |
| 394 | Q | 392 | 397 | ITQRTR | 1.409 |
| 395 | R | 393 | 398 | TQRTRT | 2.902 |
| 396 | T | 394 | 399 | QRTRTR | 3.938 |
| 397 | R | 395 | 400 | RTRTRP | 3.516 |
| 398 | T | 396 | 401 | TRTRPS | 2.406 |
| 399 | R | 397 | 402 | RTRPSV | 1.237 |
| 400 | P | 398 | 403 | TRPSVQ | 1.094 |
| 401 | S | 399 | 404 | RPSVQP | 1.172 |
| 402 | V | 400 | 405 | PSVQPG | 0.592 |
| 403 | Q | 401 | 406 | SVQPGP | 0.592 |
| 404 | P | 402 | 407 | VQPGPM | 0.437 |
| 405 | G | 403 | 408 | QPGPMI | 0.413 |
| 406 | P | 404 | 409 | PGPMID | 0.398 |
| 407 | M | 405 | 410 | GPMIDV | 0.191 |
| 408 | I | 406 | 411 | PMIDVN | 0.311 |
| 409 | D | 407 | 412 | MIDVNT | 0.29 |
| 410 | V | 408 | 413 | IDVNTD | 0.489 |

Download result

© 2005-2015 | IEDB Home
Supported by a contract from the National Institute of Allergy and Infectious Diseases, a component of the National Institutes of Health in the Department of Health and Human Services.
