# Supplementary material for: Epitope-Based Vaccine Target Screening against Highly Pathogenic MERS-CoV: An In Silico Approach Applied to Emerging Infectious Diseases
Source: PLoS One. 2015 Dec 7;10(12):e0144475. doi: 10.1371/journal.pone.0144475 (PMC4671582; doi:10.1371/journal.pone.0144475)
Supplement: S3 Fig — X-axis represents the amino acids, whereas Y-axis represents flexible propensity. Average antigenic score was 1.028. Area displayed by the threshold line is considered as potential B-cell flexible regions. (PDF) [file pone.0144475.s003.pdf]

IEDB Analysis Resource

Home | Help | Example | Reference | Download | Contact

Karplus & Schulz Flexibility Prediction Prediction

Input Sequences

```
  1 MASPAAPRAV SFADNNDITN TNLSRGRGRN PKPRAAPNNT VSWYTGLTQH GKVPLTFPPG
 61 QGVPLNANST PAQNAGYWRR QDRKINTGNG IKQLAPRWYF YYTGTGPEAA LPFRAVKDGI
121 VWVHEDGATD APSTFGTRNP NNDSAIVTQF APGTKLPKNF HIEGTGGNSQ SSSRASSVSR
181 NSSRSSSQGS RSGNSTRGTS PGPSGIGAVG GDLLYLDLLN RLQALESGKV KQSQPKVITK
241 KDAAAAKNKM RHKRTSTKSF NMVQAFGLRG PGDLQGNFGD LQLNKLGTED PRWPQIAELA
301 PTASAFMGMS QFKLTHQNND DHGNPVYFLR YSGAIKLDPK NPNYNKWLEL LEQNIDAYKT
361 FPKKEKKQKA PKEESTDQMS EPPKEQRVQG SITQRTRTRP SVQPGPMIDV NTD
```

Center position: 4 **Window size:** 7 **Threshold:** 1.0 Recalculate

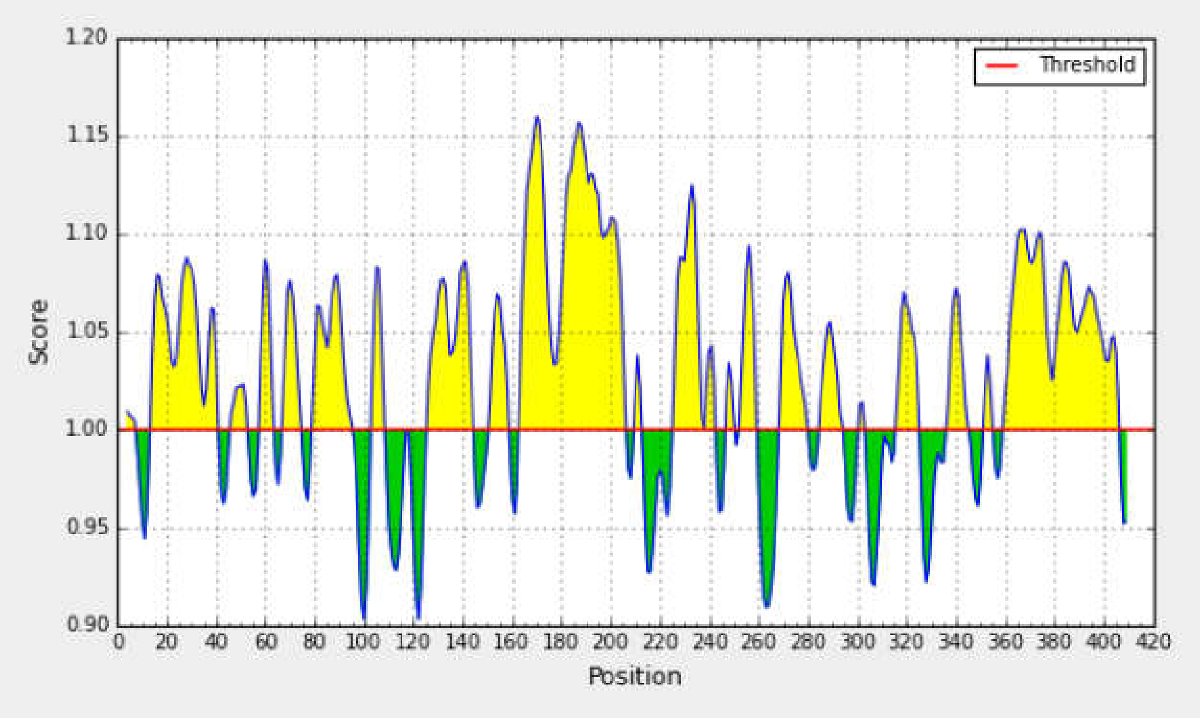

Average: 1.028 **Minimum:** 0.903 **Maximum:** 1.160

Predicted residue scores:

Position	Residue	Start	End	Peptide	Score
4	P	1	7	MASPAAP	1.009
5	A	2	8	ASPAAPR	1.007
6	A	3	9	SPAAPRA	1.006
7	P	4	10	PAAPRAV	1.004
8	R	5	11	AAPRAVS	0.989
9	A	6	12	APRAVSF	0.97
10	V	7	13	PRAVSFA	0.952
11	S	8	14	RAVSFAD	0.944

Position	Residue	Start	End	Peptide	Score
12	F	9	15	AVSFADN	0.959
13	A	10	16	VSFADNN	0.994
14	D	11	17	SFADNND	1.038
15	N	12	18	FADNNDI	1.067
16	N	13	19	ADNNDIT	1.079
17	D	14	20	DNNDITN	1.078
18	I	15	21	NNDITNT	1.068
19	T	16	22	NDITNTN	1.063
20	N	17	23	DITNTNL	1.057
21	T	18	24	ITNTNLS	1.047
22	N	19	25	TNTNLSR	1.035
23	L	20	26	NTNLSRG	1.032
24	S	21	27	TNLSRGR	1.038
25	R	22	28	NLSRGRG	1.055
26	G	23	29	LSRGRGR	1.074
27	R	24	30	SRGRGRN	1.083
28	G	25	31	RGRGRNP	1.088
29	R	26	32	GRGRNPK	1.084
30	N	27	33	RGRNPKP	1.082
31	P	28	34	GRNPKPR	1.075
32	K	29	35	RNPKPRA	1.061
33	P	30	36	NPKPRAA	1.04
34	R	31	37	PKPRAAP	1.021
35	A	32	38	KPRAAPN	1.012
36	A	33	39	PRAAPNN	1.021
37	P	34	40	RAAPNNT	1.046
38	N	35	41	AAPNNTV	1.062
39	N	36	42	APNNTVS	1.061
40	T	37	43	PNNTVSW	1.036
41	V	38	44	NNTVSWY	0.998
42	S	39	45	NTVSWYT	0.97
43	W	40	46	TVSWYTG	0.962
44	Y	41	47	VSWYTGL	0.969
45	T	42	48	SWYTGLT	0.991
46	G	43	49	WYTGLTQ	1.008
47	L	44	50	YTGLTQH	1.014
48	T	45	51	TGLTQHG	1.021
49	Q	46	52	GLTQHGK	1.022
50	H	47	53	LTQHGKV	1.022
51	G	48	54	TQHGKVP	1.023
52	K	49	55	QHGKVPL	1.014

Position	Residue	Start	End	Peptide	Score
53	V	50	56	HGKVPLT	0.994
54	P	51	57	GKVPLTF	0.974
55	L	52	58	KVPLTFP	0.966
56	T	53	59	VPLTFPP	0.969
57	F	54	60	PLTFPPG	0.993
58	P	55	61	LTFPPGQ	1.036
59	P	56	62	TFPPGQG	1.067
60	G	57	63	FPPGQGV	1.087
61	Q	58	64	PPGQGVP	1.081
62	G	59	65	PGQGVPL	1.049
63	V	60	66	GQGVPLN	1.012
64	P	61	67	QGVPLNA	0.983
65	L	62	68	GVPLNAN	0.972
66	N	63	69	VPLNANS	0.986
67	A	64	70	PLNANST	1.016
68	N	65	71	LNANSTP	1.044
69	S	66	72	NANSTPA	1.071
70	T	67	73	ANSTPAQ	1.076
71	P	68	74	NSTPAQN	1.068
72	A	69	75	STPAQNA	1.054
73	Q	70	76	TPAQNAG	1.031
74	N	71	77	PAQNAGY	1.011
75	A	72	78	AQNAGYW	0.989
76	G	73	79	QNAGYWR	0.969
77	Y	74	80	NAGYWRR	0.964
78	W	75	81	AGYWRRQ	0.979
79	R	76	82	GYWRRQD	1.008
80	R	77	83	YWRRQDR	1.037
81	Q	78	84	WRRQDRK	1.063
82	D	79	85	RRQDRKI	1.063
83	R	80	86	RQDRKIN	1.055
84	K	81	87	QDRKINT	1.048
85	I	82	88	DRKINTG	1.042
86	N	83	89	RKINTGN	1.053
87	T	84	90	KINTGNG	1.069
88	G	85	91	INTGNGI	1.077
89	N	86	92	NTGNGIK	1.079
90	G	87	93	TGNGIKQ	1.067
91	I	88	94	GNGIKQL	1.048
92	K	89	95	NGIKQLA	1.03
93	Q	90	96	GIKQLAP	1.016
94	L	91	97	IKQLAPR	1.008

Position	Residue	Start	End	Peptide	Score
95	A	92	98	KQLAPRW	1.002
96	P	93	99	QLAPRWY	0.993
97	R	94	100	LAPRWYF	0.972
98	W	95	101	APRWYFY	0.94
99	Y	96	102	PRWYFYY	0.912
100	F	97	103	RWYFYYT	0.903
101	Y	98	104	WYFYYTG	0.92
102	Y	99	105	YFYYTGT	0.963
103	T	100	106	FYYTGTG	1.018
104	G	101	107	YYTGTGP	1.061
105	T	102	108	YTGTGPE	1.083
106	G	103	109	TGTGPEA	1.082
107	P	104	110	GTGPEAA	1.052
108	E	105	111	TGPEAAL	1.016
109	A	106	112	GPEAALP	0.98
110	A	107	113	PEAALPF	0.951
111	L	108	114	EAALPFR	0.938
112	P	109	115	AALPFRA	0.93
113	F	110	116	ALPFRAV	0.928
114	R	111	117	LPFRAVK	0.937
115	A	112	118	PFRAVKD	0.954
116	V	113	119	FRAVKDG	0.977
117	K	114	120	RAVKDGI	0.999
118	D	115	121	AVKDGIV	1.0
119	G	116	122	VKDGIVW	0.979
120	I	117	123	KDGIVWV	0.944
121	V	118	124	DGIVWVH	0.915
122	W	119	125	GIVWVHE	0.903
123	V	120	126	IVWVHED	0.921
124	H	121	127	VWVHEDG	0.955
125	E	122	128	WVHEDGA	0.991
126	D	123	129	VHEDGAT	1.02
127	G	124	130	HEDGATD	1.038
128	A	125	131	EDGATDA	1.048
129	T	126	132	DGATDAP	1.055
130	D	127	133	GATDAPS	1.068
131	A	128	134	ATDAPST	1.076
132	P	129	135	TDAPSTF	1.077
133	S	130	136	DAPSTFG	1.072
134	T	131	137	APSTFGT	1.054
135	F	132	138	PSTFGTR	1.038

Position	Residue	Start	End	Peptide	Score
136	G	133	139	STFGTRN	1.04
137	T	134	140	TFGTRNP	1.046
138	R	135	141	FGTRNPN	1.06
139	N	136	142	GTRNPNN	1.08
140	P	137	143	TRNPNND	1.084
141	N	138	144	RNPNNDS	1.086
142	N	139	145	NPNNDSA	1.074
143	D	140	146	PNNDSAI	1.042
144	S	141	147	NNDSAIV	1.009
145	A	142	148	NDSAIVT	0.977
146	I	143	149	DSAIVTQ	0.96
147	V	144	150	SAIVTQF	0.962
148	T	145	151	AIVTQFA	0.971
149	Q	146	152	IVTQFAP	0.982
150	F	147	153	VTQFAPG	0.994
151	A	148	154	TQFAPGT	1.015
152	P	149	155	QFAPGTK	1.043
153	G	150	156	FAPGTKL	1.061
154	T	151	157	APGTKLP	1.069
155	K	152	158	PGTKLPK	1.067
156	L	153	159	GTKLPKN	1.053
157	P	154	160	TKLPKNF	1.043
158	K	155	161	KLPKNFH	1.024
159	N	156	162	LPKNFHI	0.991
160	F	157	163	PKNFHIE	0.965
161	H	158	164	KNFHIEG	0.957
162	I	159	165	NFHIEGT	0.972
163	E	160	166	FHIEGTG	1.015
164	G	161	167	HIEGTGG	1.062
165	T	162	168	IEGTGGN	1.094
166	G	163	169	EGTGGNS	1.121
167	G	164	170	GTGGNSQ	1.133
168	N	165	171	TGGNSQS	1.141
169	S	166	172	GGNSQSS	1.153
170	Q	167	173	GNSQSSS	1.16
171	S	168	174	NSQSSSR	1.156
172	S	169	175	SQSSSRA	1.143
173	S	170	176	QSSSRAS	1.119
174	R	171	177	SSSRASS	1.087
175	A	172	178	SSRASSV	1.061
176	S	173	179	SRASSVS	1.043
177	S	174	180	RASSVSR	1.033

Position	Residue	Start	End	Peptide	Score
178	V	175	181	ASSVSRN	1.034
179	S	176	182	SSVSRNS	1.05
180	R	177	183	SVSRNSS	1.073
181	N	178	184	VSRNSSR	1.096
182	S	179	185	SRNSSRS	1.119
183	S	180	186	RNSSRSS	1.13
184	R	181	187	NSSRSSS	1.133
185	S	182	188	SSRSSSQ	1.144
186	S	183	189	SRSSSQG	1.15
187	S	184	190	RSSSQGS	1.157
188	Q	185	191	SSSQGSR	1.155
189	G	186	192	SSQGSRS	1.145
190	S	187	193	SQGSRSG	1.137
191	R	188	194	QGSRSGN	1.126
192	S	189	195	GSRSGNS	1.131
193	G	190	196	SRSGNST	1.13
194	N	191	197	RSGNSTR	1.123
195	S	192	198	SGNSTRG	1.12
196	T	193	199	GNSTRGT	1.103
197	R	194	200	NSTRGTS	1.098
198	G	195	201	STRGTSP	1.101
199	T	196	202	TRGTSPG	1.102
200	S	197	203	RGTSPGP	1.108
201	P	198	204	GTSPGPS	1.108
202	G	199	205	TSPGPSG	1.106
203	P	200	206	SPGPSGI	1.095
204	S	201	207	PGPSGIG	1.077
205	G	202	208	GPSGIGA	1.045
206	I	203	209	PSGIGAV	1.007
207	G	204	210	SGIGAVG	0.98
208	A	205	211	GIGAVGG	0.975
209	V	206	212	IGAVGGD	0.993
210	G	207	213	GAVGGDL	1.021
211	G	208	214	AVGGDLL	1.038
212	D	209	215	VGGDLLY	1.022
213	L	210	216	GGDLLYL	0.99
214	L	211	217	GDLLYLD	0.953
215	Y	212	218	DLLYLDL	0.927
216	L	213	219	LLYLDLL	0.927
217	D	214	220	LYLDLLN	0.939
218	L	215	221	YLDLLNR	0.958

Position	Residue	Start	End	Peptide	Score
219	L	216	222	LDLLNRL	0.975
220	N	217	223	DLLNRLQ	0.979
221	R	218	224	LLNRLQA	0.977
222	L	219	225	LNRLQAL	0.965
223	Q	220	226	NRLQALE	0.956
224	A	221	227	RLQALES	0.97
225	L	222	228	LQALESG	1.0
226	E	223	229	QALESGK	1.042
227	S	224	230	ALESGKV	1.077
228	G	225	231	LESGKVK	1.088
229	K	226	232	ESGKVKQ	1.088
230	V	227	233	SGKVKQS	1.086
231	K	228	234	GKVKQSQ	1.099
232	Q	229	235	KVKQSQP	1.115
233	S	230	236	VKQSQPK	1.125
234	Q	231	237	KQSQPKV	1.115
235	P	232	238	QSQPKVI	1.076
236	K	233	239	SQPKVIT	1.038
237	V	234	240	QPKVITK	1.006
238	I	235	241	PKVITKK	1.0
239	T	236	242	KVITKKD	1.023
240	K	237	243	VITKKDA	1.04
241	K	238	244	ITKKDAA	1.043
242	D	239	245	TKKDAAA	1.024
243	A	240	246	KKDAAAA	0.983
244	A	241	247	KDAAAAK	0.958
245	A	242	248	DAAAAKN	0.959
246	A	243	249	AAAAKNK	0.985
247	K	244	250	AAAKNKM	1.019
248	N	245	251	AAKNKMR	1.034
249	K	246	252	AKNKMRH	1.026
250	M	247	253	KNKMRHK	1.006
251	R	248	254	NKMRHKR	0.992
252	H	249	255	KMRHKRT	1.0
253	K	250	256	MRHKRTS	1.028
254	R	251	257	RHKRTST	1.056
255	T	252	258	HKRTSTK	1.081
256	S	253	259	KRTSTKS	1.094
257	T	254	260	RTSTKSF	1.081
258	K	255	261	TSTKSFN	1.058
259	S	256	262	STKSFNM	1.019
260	F	257	263	TKSFNMV	0.973

Position	Residue	Start	End	Peptide	Score
261	N	258	264	KSFNMVQ	0.939
262	M	259	265	SFNMVQA	0.915
263	V	260	266	FNMVQAF	0.909
264	Q	261	267	NMVQAFG	0.91
265	A	262	268	MVQAFGL	0.919
266	F	263	269	VQAFGLR	0.932
267	G	264	270	QAFGLRG	0.956
268	L	265	271	AFGLRGP	0.989
269	R	266	272	FGLRGPG	1.028
270	G	267	273	GLRGPGD	1.065
271	P	268	274	LRGPGDL	1.077
272	G	269	275	RGPGDLQ	1.08
273	D	270	276	GPGDLQG	1.071
274	L	271	277	PGDLQGN	1.053
275	Q	272	278	GDLQGNF	1.044
276	G	273	279	DLQGNFG	1.037
277	N	274	280	LQGNFGD	1.027
278	F	275	281	QGNFGDL	1.02
279	G	276	282	GNFGDLQ	1.012
280	D	277	283	NFGDLQL	0.999
281	L	278	284	FGDLQLN	0.984
282	Q	279	285	GDLQLNK	0.979
283	L	280	286	DLQLNKL	0.981
284	N	281	287	LQLNKLG	0.993
285	K	282	288	QLNKLGT	1.014
286	L	283	289	LNKLGTE	1.029
287	G	284	290	NKLGTED	1.042
288	T	285	291	KLGTEDP	1.052
289	E	286	292	LGTEDPR	1.055
290	D	287	293	GTEDPRW	1.047
291	P	288	294	TEDPRWP	1.037
292	R	289	295	EDPRWPQ	1.026
293	W	290	296	DPRWPQI	1.008
294	P	291	297	PRWPQIA	1.002
295	Q	292	298	RWPQIAE	0.984
296	I	293	299	WPQIAEL	0.963
297	A	294	300	PQIAELA	0.954
298	E	295	301	QIAELAP	0.953
299	L	296	302	IAELAPT	0.968
300	A	297	303	AELAPTA	0.992
301	P	298	304	ELAPTAS	1.012

Position	Residue	Start	End	Peptide	Score
302	T	299	305	LAPTASA	1.014
303	A	300	306	APTASAF	1.0
304	S	301	307	PTASAFM	0.971
305	A	302	308	TASAFMG	0.942
306	F	303	309	ASAFMGM	0.922
307	M	304	310	SAFMGMS	0.92
308	G	305	311	AFMGMSQ	0.939
309	M	306	312	FMGMSQF	0.958
310	S	307	313	MGMSQFK	0.984
311	Q	308	314	GMSQFKL	0.997
312	F	309	315	MSQFKLT	0.993
313	K	310	316	SQFKLTH	0.992
314	L	311	317	QFKLTHQ	0.983
315	T	312	318	FKLTHQN	0.988
316	H	313	319	KLTHQNN	1.01
317	Q	314	320	LTHQNND	1.032
318	N	315	321	THQNNDD	1.06
319	N	316	322	HQNNDDH	1.07
320	D	317	323	QNNDDHG	1.064
321	D	318	324	NNDDHGN	1.06
322	H	319	325	NDDHGNP	1.051
323	G	320	326	DDHGNPV	1.048
324	N	321	327	DHGNPVY	1.037
325	P	322	328	HGNPVYF	1.008
326	V	323	329	GNPVYFL	0.971
327	Y	324	330	NPVYFLR	0.936
328	F	325	331	PVYFLRY	0.922
329	L	326	332	VYFLRYS	0.929
330	R	327	333	YFLRYSG	0.948
331	Y	328	334	FLRYSGA	0.972
332	S	329	335	LRYSGAI	0.984
333	G	330	336	RYSGAIK	0.988
334	A	331	337	YSGAIKL	0.983
335	I	332	338	SGAIKLD	0.983
336	K	333	339	GAIKLDP	0.998
337	L	334	340	AIKLDPK	1.015
338	D	335	341	IKLDPKN	1.046
339	P	336	342	KLDPKNP	1.065
340	K	337	343	LDPKNPN	1.072
341	N	338	344	DPKNPNY	1.068
342	P	339	345	PKNPNYN	1.047
343	N	340	346	KNPNYNK	1.031

Position	Residue	Start	End	Peptide	Score
344	Y	341	347	NPNYNKW	1.012
345	N	342	348	PNYNKWL	1.001
346	K	343	349	NYNKWLE	0.993
347	W	344	350	YNKWLEL	0.975
348	L	345	351	NKWLELL	0.965
349	E	346	352	KWLELLE	0.961
350	L	347	353	WLELLEQ	0.975
351	L	348	354	LELLEQN	1.0
352	E	349	355	ELLEQNI	1.024
353	Q	350	356	LLEQNID	1.038
354	N	351	357	LEQNIDA	1.023
355	I	352	358	EQNIDAY	1.001
356	D	353	359	QNIDAYK	0.981
357	A	354	360	NIDAYKT	0.975
358	Y	355	361	IDAYKTF	0.981
359	K	356	362	DAYKTFP	1.002
360	T	357	363	AYKTFPK	1.019
361	F	358	364	YKTFPKK	1.031
362	P	359	365	KTFPKKE	1.055
363	K	360	366	TFPKKEK	1.069
364	K	361	367	FPKKEKK	1.082
365	E	362	368	PKKEKKQ	1.097
366	K	363	369	KKEKKQK	1.102
367	K	364	370	KEKKQKA	1.102
368	Q	365	371	EKKQKAP	1.102
369	K	366	372	KKQKAPK	1.093
370	A	367	373	KQKAPKE	1.086
371	P	368	374	QKAPKEE	1.085
372	K	369	375	KAPKEES	1.089
373	E	370	376	APKEEST	1.097
374	E	371	377	PKEESTD	1.101
375	S	372	378	KEESTDQ	1.097
376	T	373	379	EESTDQM	1.074
377	D	374	380	ESTDQMS	1.051
378	Q	375	381	STDQMSE	1.035
379	M	376	382	TDQMSEP	1.025
380	S	377	383	DQMSEPP	1.036
381	E	378	384	QMSEPPK	1.051
382	P	379	385	MSEPPKE	1.062
383	P	380	386	SEPPKEQ	1.078
384	K	381	387	EPPKEQR	1.086

Position	Residue	Start	End	Peptide	Score
385	E	382	388	PPKEQRV	1.085
386	Q	383	389	PKEQRVQ	1.079
387	R	384	390	KEQRVQG	1.065
388	V	385	391	EQRVQGS	1.053
389	Q	386	392	QRVQGSI	1.05
390	G	387	393	RVQGSIT	1.053
391	S	388	394	VQGSITQ	1.058
392	I	389	395	QGSITQR	1.062
393	T	390	396	GSITQRT	1.068
394	Q	391	397	SITQRTR	1.073
395	R	392	398	ITQRTRT	1.07
396	T	393	399	TQRTRTR	1.068
397	R	394	400	QRTRTRP	1.06
398	T	395	401	RTRTRPS	1.054
399	R	396	402	TRTRPSV	1.048
400	P	397	403	RTRPSVQ	1.041
401	S	398	404	TRPSVQP	1.035
402	V	399	405	RPSVQPG	1.035
403	Q	400	406	PSVQPGP	1.046
404	P	401	407	SVQPGPM	1.048
405	G	402	408	VQPGPMI	1.04
406	P	403	409	QPGPMID	1.013
407	M	404	410	PGPMIDV	0.973
408	I	405	411	GPMIDVN	0.952
409	D	406	412	PMIDVNT	0.953

Download result

© 2005-2015 | IEDB Home
Supported by a contract from the National Institute of Allergy and Infectious Diseases, a component of the National Institutes of Health in the Department of Health and Human Services.
