# Supplementary material for: Epitope-Based Vaccine Target Screening against Highly Pathogenic MERS-CoV: An In Silico Approach Applied to Emerging Infectious Diseases
Source: PLoS One. 2015 Dec 7;10(12):e0144475. doi: 10.1371/journal.pone.0144475 (PMC4671582; doi:10.1371/journal.pone.0144475)
Supplement: S2 Table — (DOC) [file pone.0144475.s005.doc]

**Supplementary data**

**Table S2. 9-mer peptide core sequences in Nucleocapsid (N) protein of MERS-CoV predicted to be helper T-cell epitope using NetMHCIIpan 3.0 web server.**

| **Epitope No.** | **Epitope core (9 -mer)** | **Amino Acid Position** | **No. of binding HLA-DR alleles** | **HLA-DR alleles predicted to bind to the epitope** |
| --- | --- | --- | --- | --- |
| **1** | FNMVQAFGL | 260-268 | 173 | HLA-DRB1*01:01,HLA-DRB1*04:10,HLA-DRB1*04:28,HLA-DRB1*01:02, HLA-DRB1*04:67, HLA-DRB1*04:86,HLA-DRB1*07:01,HLA-DRB1*07:03,HLA-DRB1*01:04, HLA-DRB1*07:05, HLA-DRB1*07:06,HLA-DRB1*07:07,HLA-DRB1*07:08,HLA-DRB1*07:09, HLA-DRB1*01:05, HLA-DRB1*07:11,HLA-DRB1*07:12,HLA-DRB1*07:13,HLA-DRB1*07:14, HLA-DRB1*07:15, HLA-DRB1*01:06,HLA-DRB1*07:16,HLA-DRB1*07:17,HLA-DRB1*07:19, HLA-DRB1*08:05, HLA-DRB1*08:06,HLA-DRB1*01:07,HLA-DRB1*08:10,HLA-DRB1*01:08, HLA-DRB1*01:09, HLA-DRB1*01:10,HLA-DRB1*01:11,HLA-DRB1*01:12,HLA-DRB1*01:13, HLA-DRB1*01:14, HLA-DRB1*01:15,HLA-DRB1*01:16,HLA-DRB1*01:17,HLA-DRB1*01:18, HLA-DRB1*01:19, HLA-DRB1*01:20,HLA-DRB1*01:21,HLA-DRB1*01:22,HLA-DRB1*01:23, HLA-DRB1*01:24, HLA-DRB1*01:25,HLA-DRB1*01:26,HLA-DRB1*01:27,HLA-DRB1*01:28, HLA-DRB1*01:29, HLA-DRB1*01:30,HLA-DRB1*01:31,HLA-DRB1*01:32,HLA-DRB1*08:22, HLA-DRB1*09:01, HLA-DRB1*09:03,HLA-DRB1*09:04,HLA-DRB1*09:05,HLA-DRB1*09:06, HLA-DRB1*09:07, HLA-DRB1*09:08,HLA-DRB1*09:09,HLA-DRB1*10:02,HLA-DRB1*11:04, HLA-DRB1*11:06, HLA-DRB1*11:08,HLA-DRB1*11:13,HLA-DRB1*11:18,HLA-DRB1*11:34, HLA-DRB1*11:35, HLA-DRB1*11:38,HLA-DRB1*11:42,HLA-DRB1*11:43,HLA-DRB1*11:44, HLA-DRB1*11:46, HLA-DRB1*11:47,HLA-DRB1*11:57,HLA-DRB1*11:58,HLA-DRB1*11:60, HLA-DRB1*11:77, HLA-DRB1*11:78,HLA-DRB1*11:92,HLA-DRB1*12:01,HLA-DRB1*12:02, HLA-DRB1*12:03, HLA-DRB1*12:04,HLA-DRB1*12:05,HLA-DRB1*12:06,HLA-DRB1*12:07, HLA-DRB1*12:09,  HLA-DRB1*12:08,HLA-DRB1*12:10,HLA-DRB1*12:11,HLA-DRB1*12:12, HLA-DRB1*12:13, HLA-DRB1*12:14,HLA-DRB1*12:15,HLA-DRB1*12:17,HLA-DRB1*12:18, HLA-DRB1*12:19, HLA-DRB1*12:20,HLA-DRB1*12:21,HLA-DRB1*12:23,HLA-DRB1*13:06, HLA-DRB1*13:100, HLA-DRB1*13:11,HLA-DRB1*13:12,HLA-DRB1*13:21,HLA-DRB1*13:30, HLA-DRB1*13:42, HLA-DRB1*13:43,HLA-DRB1*13:44,HLA-DRB1*13:46,HLA-DRB1*13:49, HLA-DRB1*13:56,  HLA-DRB1*13:58,HLA-DRB1*13:60,HLA-DRB1*13:75, HLA-DRB1*13:77, HLA-DRB1*13:81, HLA-DRB1*13:88,HLA-DRB1*13:89,HLA-DRB1*14:06, HLA-DRB1*14:13, HLA-DRB1*14:17, HLA-DRB1*14:20,HLA-DRB1*14:21,HLA-DRB1*14:30,HLA-DRB1*14:31, HLA-DRB1*14:32, HLA-DRB1*14:34,HLA-DRB1*14:46,HLA-DRB1*14:48,HLA-DRB1*14:49, HLA-DRB1*14:52, HLA-DRB1*14:53,HLA-DRB1*14:65,HLA-DRB1*14:69,HLA-DRB1*14:74, HLA-DRB1*14:78, HLA-DRB1*14:80,HLA-DRB1*14:83,HLA-DRB1*14:84,HLA-DRB1*15:01, HLA-DRB1*15:02, HLA-DRB1*15:03,HLA-DRB1*15:08,HLA-DRB1*15:09,HLA-DRB1*15:12, HLA-DRB1*15:13, HLA-DRB1*15:14,HLA-DRB1*15:16,HLA-DRB1*15:19,HLA-DRB1*15:20, HLA-DRB1*15:22, HLA-DRB1*15:23,HLA-DRB1*15:24,HLA-DRB1*15:26,HLA-DRB1*15:28, HLA-DRB1*15:29, HLA-DRB1*15:31,HLA-DRB1*15:32,HLA-DRB1*15:33,HLA-DRB1*15:36, HLA-DRB1*15:38, HLA-DRB1*15:39,HLA-DRB1*15:40,HLA-DRB1*15:41,HLA-DRB1*15:42, HLA-DRB1*15:43, HLA-DRB1*15:44,HLA-DRB1*15:45,HLA-DRB1*15:46,HLA-DRB1*15:47, HLA-DRB1*15:49, HLA-DRB1*16:02, HLA-DRB1*16:10, HLA-DRB1*16:12, HLA-DRB1*16:14 |
| 2 | LRYSGAIKL | 329-337 | 356 | HLA-DRB1*01:01,HLA-DRB1*04:10,HLA-DRB1*04:18, HLA-DRB1*01:02, HLA-DRB1*04:12, HLA-DRB1*04:13,HLA-DRB1*04:27,HLA-DRB1*01:03, HLA-DRB1*01:04, HLA-DRB1*04:11, HLA-DRB1*04:42,HLA-DRB1*04:55,HLA-DRB1*04:58,HLA-DRB1*01:05, HLA-DRB1*04:65, HLA-DRB1*04:67,HLA-DRB1*04:64,HLA-DRB1*01:06,HLA-DRB1*04:66, HLA-DRB1*04:86, HLA-DRB1*04:88,HLA-DRB1*04:91,HLA-DRB1*01:07,HLA-DRB1*07:01, HLA-DRB1*07:03, HLA-DRB1*07:04,HLA-DRB1*07:05,HLA-DRB1*07:06,HLA-DRB1*01:08, HLA-DRB1*07:07, HLA-DRB1*07:08,HLA-DRB1*07:09,HLA-DRB1*07:11,HLA-DRB1*07:12, HLA-DRB1*01:09, HLA-DRB1*07:13,HLA-DRB1*07:14,HLA-DRB1*07:15,HLA-DRB1*07:16, HLA-DRB1*07:17, HLA-DRB1*01:10,HLA-DRB1*07:19,HLA-DRB1*08:03,HLA-DRB1*08:05, HLA-DRB1*08:06, HLA-DRB1*08:10,HLA-DRB1*01:11,HLA-DRB1*08:12,HLA-DRB1*08:14, HLA-DRB1*08:18, HLA-DRB1*08:20,HLA-DRB1*08:22,HLA-DRB1*01:12,HLA-DRB1*08:23, HLA-DRB1*08:25, HLA-DRB1*08:27,HLA-DRB1*08:31,HLA-DRB1*08:32,HLA-DRB1*01:13, HLA-DRB1*08:33, HLA-DRB1*08:35,HLA-DRB1*08:36,HLA-DRB1*08:37,HLA-DRB1*08:38, HLA-DRB1*01:14, HLA-DRB1*08:40,HLA-DRB1*09:01,HLA-DRB1*09:02,HLA-DRB1*09:03, HLA-DRB1*09:04, HLA-DRB1*01:15,HLA-DRB1*09:05,HLA-DRB1*09:06,HLA-DRB1*09:07, HLA-DRB1*09:08, HLA-DRB1*09:09,HLA-DRB1*01:16,HLA-DRB1*10:01,HLA-DRB1*10:02, HLA-DRB1*10:03, HLA-DRB1*11:02,HLA-DRB1*11:03,HLA-DRB1*01:17,HLA-DRB1*11:04, HLA-DRB1*01:18, HLA-DRB1*01:19,HLA-DRB1*01:20,HLA-DRB1*01:21,HLA-DRB1*01:22, HLA-DRB1*01:23, HLA-DRB1*01:24,HLA-DRB1*01:25,HLA-DRB1*01:26,HLA-DRB1*01:27, HLA-DRB1*01:28, HLA-DRB1*01:29,HLA-DRB1*01:30,HLA-DRB1*01:31,HLA-DRB1*01:32, HLA-DRB1*03:03, HLA-DRB1*03:06,HLA-DRB1*03:07,HLA-DRB1*03:10,HLA-DRB1*03:11, HLA-DRB1*03:14, HLA-DRB1*03:15,HLA-DRB1*03:21,HLA-DRB1*03:24,HLA-DRB1*03:26, HLA-DRB1*03:31, HLA-DRB1*03:38,HLA-DRB1*03:49,HLA-DRB1*11:06,HLA-DRB1*11:07, HLA-DRB1*11:11, HLA-DRB1*11:13,HLA-DRB1*11:14,HLA-DRB1*11:16,HLA-DRB1*11:17, HLA-DRB1*11:18, HLA-DRB1*11:20,HLA-DRB1*11:21,HLA-DRB1*11:25,HLA-DRB1*11:30, HLA-DRB1*11:34, HLA-DRB1*11:35,HLA-DRB1*11:36,HLA-DRB1*11:38,HLA-DRB1*11:41, HLA-DRB1*11:42, HLA-DRB1*11:43,HLA-DRB1*11:44,HLA-DRB1*11:46,HLA-DRB1*11:47, HLA-DRB1*11:48, HLA-DRB1*11:50,HLA-DRB1*11:52,HLA-DRB1*11:55,HLA-DRB1*11:56, HLA-DRB1*11:57, HLA-DRB1*11:59,HLA-DRB1*11:60,HLA-DRB1*11:63,HLA-DRB1*11:65, HLA-DRB1*11:68, HLA-DRB1*11:70,HLA-DRB1*11:72,HLA-DRB1*11:73,HLA-DRB1*11:76, HLA-DRB1*11:77, HLA-DRB1*11:78,HLA-DRB1*11:79,HLA-DRB1*11:83,HLA-DRB1*11:84, HLA-DRB1*11:85, HLA-DRB1*11:86,HLA-DRB1*11:88,HLA-DRB1*11:92,HLA-DRB1*12:01, HLA-DRB1*12:02, HLA-DRB1*12:03,HLA-DRB1*12:04,HLA-DRB1*12:05,HLA-DRB1*12:06, HLA-DRB1*12:07, HLA-DRB1*12:08,HLA-DRB1*12:09,HLA-DRB1*12:10,HLA-DRB1*12:11, HLA-DRB1*12:12, HLA-DRB1*12:13,HLA-DRB1*12:14,HLA-DRB1*12:15,HLA-DRB1*12:17, HLA-DRB1*12:18, HLA-DRB1*12:19,HLA-DRB1*12:20,HLA-DRB1*12:21,HLA-DRB1*12:22, HLA-DRB1*12:23, HLA-DRB1*13:01,HLA-DRB1*13:02,HLA-DRB1*13:03,HLA-DRB1*13:04, HLA-DRB1*13:06, HLA-DRB1*13:08,HLA-DRB1*13:09,HLA-DRB1*13:10,HLA-DRB1*13:101, HLA-DRB1*13:11, HLA-DRB1*13:12,HLA-DRB1*13:13,HLA-DRB1*13:15,HLA-DRB1*13:16, HLA-DRB1*13:17, HLA-DRB1*13:18,HLA-DRB1*13:19,HLA-DRB1*13:20,HLA-DRB1*13:21, HLA-DRB1*13:22, HLA-DRB1*13:23,HLA-DRB1*13:24,HLA-DRB1*13:27,HLA-DRB1*13:29, HLA-DRB1*13:30, HLA-DRB1*13:32,HLA-DRB1*13:33,HLA-DRB1*13:34,HLA-DRB1*13:35, HLA-DRB1*13:36 HLA-DRB1*13:37,HLA-DRB1*13:38,HLA-DRB1*13:39, HLA-DRB1*13:41, HLA-DRB1*13:42, HLA-DRB1*13:43,HLA-DRB1*13:44,HLA-DRB1*13:48, HLA-DRB1*13:51, HLA-DRB1*13:52, HLA-DRB1*13:53,HLA-DRB1*13:54,HLA-DRB1*13:55,HLA-DRB1*13:57, HLA-DRB1*13:58, HLA-DRB1*13:65,HLA-DRB1*13:59,HLA-DRB1*13:63,HLA-DRB1*13:64, HLA-DRB1*13:66, HLA-DRB1*13:68,HLA-DRB1*13:69,HLA-DRB1*13:70,HLA-DRB1*13:71, HLA-DRB1*13:72, HLA-DRB1*13:73,HLA-DRB1*13:74,HLA-DRB1*13:75,HLA-DRB1*13:76, HLA-DRB1*13:77, HLA-DRB1*13:78,HLA-DRB1*13:79,HLA-DRB1*13:80,HLA-DRB1*13:81, HLA-DRB1*13:83, HLA-DRB1*13:84,HLA-DRB1*13:85,HLA-DRB1*13:86,HLA-DRB1*13:87, HLA-DRB1*13:88, HLA-DRB1*13:89,HLA-DRB1*13:90,HLA-DRB1*13:91,HLA-DRB1*13:92, HLA-DRB1*13:93, HLA-DRB1*13:94,HLA-DRB1*13:95,HLA-DRB1*13:96,HLA-DRB1*13:97, HLA-DRB1*13:98, HLA-DRB1*13:99,HLA-DRB1*14:01,HLA-DRB1*14:04,HLA-DRB1*14:05, HLA-DRB1*14:06, HLA-DRB1*14:08,HLA-DRB1*14:12,HLA-DRB1*14:13,HLA-DRB1*14:16, HLA-DRB1*14:17, HLA-DRB1*14:18,HLA-DRB1*14:19,HLA-DRB1*14:21,HLA-DRB1*14:20, HLA-DRB1*14:23, HLA-DRB1*14:24,HLA-DRB1*14:26,HLA-DRB1*14:29,HLA-DRB1*14:31, HLA-DRB1*14:32, HLA-DRB1*14:33,HLA-DRB1*14:34,HLA-DRB1*14:35, HLA-DRB1*14:37, HLA-DRB1*14:38, HLA-DRB1*14:39,HLA-DRB1*14:43,HLA-DRB1*14:45,HLA-DRB1*14:46, HLA-DRB1*14:47, HLA-DRB1*14:48,HLA-DRB1*14:49,HLA-DRB1*14:52,HLA-DRB1*14:53, HLA-DRB1*14:54, HLA-DRB1*14:56,HLA-DRB1*14:58,HLA-DRB1*14:59,HLA-DRB1*14:60, HLA-DRB1*14:61, HLA-DRB1*14:62,HLA-DRB1*14:63,HLA-DRB1*14:64,HLA-DRB1*14:65, HLA-DRB1*14:67, HLA-DRB1*14:69,HLA-DRB1*14:70,HLA-DRB1*14:72,HLA-DRB1*14:74, HLA-DRB1*14:75, HLA-DRB1*14:76,HLA-DRB1*14:78,HLA-DRB1*14:79,HLA-DRB1*14:80, HLA-DRB1*14:81, HLA-DRB1*14:82,HLA-DRB1*14:83,HLA-DRB1*14:84,HLA-DRB1*14:85, HLA-DRB1*14:86, HLA-DRB1*14:87,HLA-DRB1*14:88,HLA-DRB1*14:91,HLA-DRB1*14:95, HLA-DRB1*14:96, HLA-DRB1*14:97,HLA-DRB1*14:99,HLA-DRB1*15:01,HLA-DRB1*15:03, HLA-DRB1*15:07, HLA-DRB1*15:09,HLA-DRB1*15:10,HLA-DRB1*15:12,HLA-DRB1*15:13, HLA-DRB1*15:16, HLA-DRB1*15:18,HLA-DRB1*15:20,HLA-DRB1*15:21,HLA-DRB1*15:22, HLA-DRB1*15:23, HLA-DRB1*15:24,HLA-DRB1*15:28,HLA-DRB1*15:30,HLA-DRB1*15:32, HLA-DRB1*15:33, HLA-DRB1*15:35,HLA-DRB1*15:36,HLA-DRB1*15:37,HLA-DRB1*15:40, HLA-DRB1*15:41, HLA-DRB1*15:42,HLA-DRB1*15:43,HLA-DRB1*15:45,HLA-DRB1*15:46, HLA-DRB1*15:48, HLA-DRB1*15:49 |
| 3 | LQALESGKV | 222-230 | 139 | HLA-DRB1*01:01, HLA-DRB1*04:34, HLA-DRB1*04:10, HLA-DRB1*04:12, HLA-DRB1*04:64, HLA-DRB1*01:02, HLA-DRB1*04:66, HLA-DRB1*04:67, HLA-DRB1*01:04, HLA-DRB1*07:01, HLA-DRB1*07:03, HLA-DRB1*07:05, HLA-DRB1*07:06, HLA-DRB1*07:07, HLA-DRB1*01:05, HLA-DRB1*07:08, HLA-DRB1*07:09, HLA-DRB1*07:12, HLA-DRB1*07:13, HLA-DRB1*07:14, HLA-DRB1*01:06, HLA-DRB1*07:15, HLA-DRB1*07:16, HLA-DRB1*07:17, HLA-DRB1*07:19, HLA-DRB1*08:05, HLA-DRB1*01:07, HLA-DRB1*08:10, HLA-DRB1*08:12, HLA-DRB1*08:18, HLA-DRB1*08:40, HLA-DRB1*01:08, HLA-DRB1*09:03, HLA-DRB1*09:04, HLA-DRB1*09:05, HLA-DRB1*09:06, HLA-DRB1*09:08, HLA-DRB1*01:09, HLA-DRB1*11:04, HLA-DRB1*01:10, HLA-DRB1*01:11, HLA-DRB1*01:12, HLA-DRB1*01:13, HLA-DRB1*01:14, HLA-DRB1*01:15, HLA-DRB1*01:16, HLA-DRB1*01:17, HLA-DRB1*01:18, HLA-DRB1*01:19, HLA-DRB1*01:20, HLA-DRB1*01:21, HLA-DRB1*01:22, HLA-DRB1*01:23, HLA-DRB1*01:24, HLA-DRB1*01:25, HLA-DRB1*01:26, HLA-DRB1*01:27, HLA-DRB1*01:28, HLA-DRB1*01:29, HLA-DRB1*01:30, HLA-DRB1*01:31, HLA-DRB1*01:32, HLA-DRB1*04:10, HLA-DRB1*04:12, HLA-DRB1*04:13, HLA-DRB1*04:12,HLA-DRB1*11:08, HLA-DRB1*11:13, HLA-DRB1*11:18, HLA-DRB1*11:34, HLA-DRB1*11:35, HLA-DRB1*11:36, HLA-DRB1*11:38, HLA-DRB1*11:42, HLA-DRB1*11:43, HLA-DRB1*11:44, HLA-DRB1*11:46, HLA-DRB1*11:57, HLA-DRB1*11:58, HLA-DRB1*11:60, HLA-DRB1*11:72, HLA-DRB1*11:73, HLA-DRB1*11:77, HLA-DRB1*11:78, HLA-DRB1*11:79, HLA-DRB1*11:92, HLA-DRB1*12:11, HLA-DRB1*12:12, HLA-DRB1*12:21, HLA-DRB1*13:03, HLA-DRB1*13:04, HLA-DRB1*13:06, HLA-DRB1*13:09, HLA-DRB1*13:10, HLA-DRB1*13:101, HLA-DRB1*13:11, HLA-DRB1*13:12, HLA-DRB1*13:20, HLA-DRB1*13:21, HLA-DRB1*13:30, HLA-DRB1*13:42, HLA-DRB1*13:43, HLA-DRB1*13:44, HLA-DRB1*13:56, HLA-DRB1*13:58, HLA-DRB1*13:60, HLA-DRB1*13:66, HLA-DRB1*13:71, HLA-DRB1*13:75, HLA-DRB1*13:77, HLA-DRB1*13:78, HLA-DRB1*13:81, HLA-DRB1*13:88, HLA-DRB1*13:89, HLA-DRB1*13:90, HLA-DRB1*13:93, HLA-DRB1*13:94, HLA-DRB1*13:95, HLA-DRB1*14:06, HLA-DRB1*14:13, HLA-DRB1*14:17, HLA-DRB1*14:20, HLA-DRB1*14:21, HLA-DRB1*14:29, HLA-DRB1*14:31, HLA-DRB1*14:32, HLA-DRB1*14:34, HLA-DRB1*14:52, HLA-DRB1*14:55, HLA-DRB1*14:65, HLA-DRB1*14:69, HLA-DRB1*14:74, HLA-DRB1*14:78, HLA-DRB1*14:80, HLA-DRB1*14:81, HLA-DRB1*14:83, HLA-DRB1*14:90, HLA-DRB1*15:12, HLA-DRB1*15:49 |
| 4 | LNRLQALES | 219-227 | 144 | HLA-DRB1*01:01, HLA-DRB1*04:18, HLA-DRB1*04:34, HLA-DRB1*04:10,HLA-DRB1*04:55, HLA-DRB1*01:02, HLA-DRB1*04:58, HLA-DRB1*04:67, HLA-DRB1*04:64,HLA-DRB1*01:04, HLA-DRB1*04:86, HLA-DRB1*08:05, HLA-DRB1*08:06, HLA-DRB1*08:10,HLA-DRB1*08:12, HLA-DRB1*01:05, HLA-DRB1*08:18, HLA-DRB1*08:22, HLA-DRB1*08:28,HLA-DRB1*08:31, HLA-DRB1*08:32, HLA-DRB1*01:06, HLA-DRB1*08:37, HLA-DRB1*08:40,HLA-DRB1*09:03, HLA-DRB1*09:06, HLA-DRB1*10:01, HLA-DRB1*01:07, HLA-DRB1*10:02,HLA-DRB1*10:03, HLA-DRB1*11:04, HLA-DRB1*01:08, HLA-DRB1*01:09, HLA-DRB1*01:10, HLA-DRB1*01:11, HLA-DRB1*01:12, HLA-DRB1*01:13, HLA-DRB1*01:14, HLA-DRB1*01:15,HLA-DRB1*01:17, HLA-DRB1*01:18, HLA-DRB1*01:19, HLA-DRB1*01:20, HLA-DRB1*01:21,HLA-DRB1*01:22, HLA-DRB1*01:23, HLA-DRB1*01:24, HLA-DRB1*01:25, HLA-DRB1*01:26,HLA-DRB1*01:27, HLA-DRB1*01:28, HLA-DRB1*01:29, HLA-DRB1*01:30, HLA-DRB1*01:31,HLA-DRB1*01:32, HLA-DRB1*04:12, HLA-DRB1*04:13, HLA-DRB1*04:10, HLA-DRB1*04:12,HLA-DRB1*04:18, HLA-DRB1*11:06, HLA-DRB1*11:08, HLA-DRB1*11:13, HLA-DRB1*11:18, HLA-DRB1*11:34, HLA-DRB1*11:35, HLA-DRB1*11:38, HLA-DRB1*11:42, HLA-DRB1*11:43, HLA-DRB1*11:44, HLA-DRB1*11:46, HLA-DRB1*11:47, HLA-DRB1*11:50, HLA-DRB1*11:56, HLA-DRB1*11:57, HLA-DRB1*11:58, HLA-DRB1*11:60, HLA-DRB1*11:67, HLA-DRB1*11:77, HLA-DRB1*11:78, HLA-DRB1*11:88, HLA-DRB1*11:92, HLA-DRB1*12:01, HLA-DRB1*12:02, HLA-DRB1*12:03, HLA-DRB1*12:04, HLA-DRB1*12:05, HLA-DRB1*12:06, HLA-DRB1*12:07,HLA-DRB1*12:08, HLA-DRB1*12:09, HLA-DRB1*12:10, HLA-DRB1*12:11, HLA-DRB1*12:12,HLA-DRB1*12:13, HLA-DRB1*12:14, HLA-DRB1*12:15, HLA-DRB1*12:17, HLA-DRB1*12:18,HLA-DRB1*12:19, HLA-DRB1*12:20, HLA-DRB1*12:21, HLA-DRB1*12:23, HLA-DRB1*13:03,HLA-DRB1*13:04, HLA-DRB1*13:06, HLA-DRB1*13:11, HLA-DRB1*13:12, HLA-DRB1*13:21,HLA-DRB1*13:30, HLA-DRB1*13:32, HLA-DRB1*13:42, HLA-DRB1*13:43, HLA-DRB1*13:44,HLA-DRB1*13:48, HLA-DRB1*13:56, HLA-DRB1*13:58, HLA-DRB1*13:66, HLA-DRB1*13:75,HLA-DRB1*13:77, HLA-DRB1*13:81, HLA-DRB1*13:88, HLA-DRB1*13:89, HLA-DRB1*13:90,HLA-DRB1*13:93, HLA-DRB1*13:94, HLA-DRB1*13:95, HLA-DRB1*14:12, HLA-DRB1*14:13,HLA-DRB1*14:17, HLA-DRB1*14:21, HLA-DRB1*14:29, HLA-DRB1*14:31, HLA-DRB1*14:32,HLA-DRB1*14:52, HLA-DRB1*14:55, HLA-DRB1*14:65, HLA-DRB1*14:69, HLA-DRB1*14:74,HLA-DRB1*14:78, HLA-DRB1*14:80, HLA-DRB1*14:81, HLA-DRB1*14:84, HLA-DRB1*14:85 |
| 5 | YLDLLNRLQ | 215-223 | 65 | HLA-DRB1*01:01, HLA-DRB1*04:67, HLA-DRB1*04:87, HLA-DRB1*08:05,HLA-DRB1*10:02, HLA-DRB1*01:02, HLA-DRB1*01:04, HLA-DRB1*01:05, HLA-DRB1*01:06,HLA-DRB1*01:07, HLA-DRB1*01:08, HLA-DRB1*01:09, HLA-DRB1*01:10, HLA-DRB1*01:11,HLA-DRB1*01:12, HLA-DRB1*01:13, HLA-DRB1*01:14, HLA-DRB1*01:16, HLA-DRB1*01:17,HLA-DRB1*01:18, HLA-DRB1*01:19, HLA-DRB1*01:22, HLA-DRB1*01:23, HLA-DRB1*01:25,HLA-DRB1*01:26, HLA-DRB1*01:27, HLA-DRB1*01:28, HLA-DRB1*01:29, HLA-DRB1*01:30,HLA-DRB1*01:31, HLA-DRB1*01:32, HLA-DRB1*04:28, HLA-DRB1*04:10, HLA-DRB1*04:34,HLA-DRB1*11:08, HLA-DRB1*13:03, HLA-DRB1*13:12, HLA-DRB1*13:49, HLA-DRB1*13:66,HLA-DRB1*13:90, HLA-DRB1*13:95, HLA-DRB1*14:13, HLA-DRB1*14:30, HLA-DRB1*14:34,HLA-DRB1*14:41, HLA-DRB1*14:48, HLA-DRB1*14:49, HLA-DRB1*15:23, HLA-DRB1*01:21,HLA-DRB1*01:24, HLA-DRB1*08:18, HLA-DRB1*08:40, HLA-DRB1*11:13, HLA-DRB1*11:47, HLA-DRB1*11:72, HLA-DRB1*11:79, HLA-DRB1*13:10,HLA-DRB1*13:101,HLA-DRB1*13:56,HLA-DRB1*13:60, HLA-DRB1*13:86, HLA-DRB1*14:02, HLA-DRB1*14:09, HLA-DRB1*14:69, HLA-DRB1*14:94 |
| 6 | FMGMSQFKL | 306-314 | 100 | HLA-DRB1*01:01,HLA-DRB1*08:10, HLA-DRB1*08:12, HLA-DRB1*08:18, HLA-DRB1*08:22, HLA-DRB1*08:40,HLA-DRB1*01:02, HLA-DRB1*11:04, HLA-DRB1*01:05, HLA-DRB1*01:06, HLA-DRB1*01:07, HLA-DRB1*01:08, HLA-DRB1*01:10, HLA-DRB1*01:11,HLA-DRB1*01:12, HLA-DRB1*01:14, HLA-DRB1*01:18, HLA-DRB1*01:19, HLA-DRB1*01:20,HLA-DRB1*01:21, HLA-DRB1*01:22, HLA-DRB1*01:23, HLA-DRB1*01:25, HLA-DRB1*01:26,HLA-DRB1*01:28, HLA-DRB1*01:30, HLA-DRB1*01:31, HLA-DRB1*01:32, HLA-DRB1*04:28,HLA-DRB1*08:05, HLA-DRB1*11:06, HLA-DRB1*11:08, HLA-DRB1*11:13,HLA-DRB1*11:18, HLA-DRB1*11:34, HLA-DRB1*11:35, HLA-DRB1*11:38, HLA-DRB1*11:42, HLA-DRB1*11:43, HLA-DRB1*11:44, HLA-DRB1*11:46, HLA-DRB1*11:47, HLA-DRB1*11:57, HLA-DRB1*11:58, HLA-DRB1*11:60, HLA-DRB1*11:77, HLA-DRB1*11:78, HLA-DRB1*11:92, HLA-DRB1*12:01, HLA-DRB1*12:02, HLA-DRB1*12:03, HLA-DRB1*12:05, HLA-DRB1*12:06, HLA-DRB1*12:07,HLA-DRB1*12:08, HLA-DRB1*12:10, HLA-DRB1*12:11, HLA-DRB1*12:12, HLA-DRB1*12:13,HLA-DRB1*12:14, HLA-DRB1*12:15, HLA-DRB1*12:17, HLA-DRB1*12:18, HLA-DRB1*12:19,HLA-DRB1*12:20, HLA-DRB1*12:21, HLA-DRB1*12:23, HLA-DRB1*13:06, HLA-DRB1*13:11,HLA-DRB1*13:12, HLA-DRB1*13:21, HLA-DRB1*13:30, HLA-DRB1*13:42, HLA-DRB1*13:44,HLA-DRB1*13:49, HLA-DRB1*13:56, HLA-DRB1*13:58, HLA-DRB1*13:60, HLA-DRB1*13:66,HLA-DRB1*13:75, HLA-DRB1*13:81, HLA-DRB1*13:86, HLA-DRB1*13:88, HLA-DRB1*13:89,HLA-DRB1*13:94, HLA-DRB1*14:06, HLA-DRB1*14:13, HLA-DRB1*14:17, HLA-DRB1*14:20,HLA-DRB1*14:29,  HLA-DRB1*14:52, HLA-DRB1*14:65, HLA-DRB1*14:69, HLA-DRB1*14:74,HLA-DRB1*14:78, HLA-DRB1*14:80, HLA-DRB1*14:83, HLA-DRB1*14:85, HLA-DRB1*15:23, HLA-DRB1*15:49 |
| 7 | FRAVKDGIV | 113-121 | 42 | HLA-DRB1*01:01, HLA-DRB1*09:04, HLA-DRB1*09:06, HLA-DRB1*01:02,HLA-DRB1*01:04, HLA-DRB1*01:05,HLA-DRB1*01:06, HLA-DRB1*01:07, HLA-DRB1*01:08, HLA-DRB1*01:09, HLA-DRB1*01:10, HLA-DRB1*01:11, HLA-DRB1*01:12, HLA-DRB1*01:13,HLA-DRB1*01:14, HLA-DRB1*01:15, HLA-DRB1*01:16, HLA-DRB1*01:17,HLA-DRB1*01:18, HLA-DRB1*01:19, HLA-DRB1*01:20, HLA-DRB1*01:21, HLA-DRB1*01:22, HLA-DRB1*01:23,HLA-DRB1*01:24, HLA-DRB1*01:25, HLA-DRB1*01:26, HLA-DRB1*01:27, HLA-DRB1*01:28,HLA-DRB1*01:30, HLA-DRB1*01:31, HLA-DRB1*01:32, HLA-DRB1*09:03, HLA-DRB1*13:10,HLA-DRB1*14:13, HLA-DRB1*13:101,HLA-DRB1*13:66, HLA-DRB1*13:81,HLA-DRB1*13:88,HLA-DRB1*13:89, HLA-DRB1*13:94,HLA-DRB1*14:80 |
| 8 | IAELAPTAS | 296-304 | 30 | HLA-DRB1*01:01, HLA-DRB1*08:10, HLA-DRB1*01:02, HLA-DRB1*01:04,HLA-DRB1*01:05, HLA-DRB1*01:06,HLA-DRB1*01:07, HLA-DRB1*01:08, HLA-DRB1*01:09, HLA-DRB1*01:10, HLA-DRB1*01:11, HLA-DRB1*01:12,HLA-DRB1*01:15, HLA-DRB1*01:19, HLA-DRB1*01:20, HLA-DRB1*01:21, HLA-DRB1*01:22, HLA-DRB1*01:23,HLA-DRB1*01:24, HLA-DRB1*01:25, HLA-DRB1*01:26, HLA-DRB1*01:27, HLA-DRB1*01:28, HLA-DRB1*01:30,HLA-DRB1*01:31, HLA-DRB1*01:32, HLA-DRB1*08:06, HLA-DRB1*13:58, HLA-DRB1*13:81, HLA-DRB1*13:89 |
| 9 | IVTQFAPGT | 146-154 | 42 | HLA-DRB1*01:01, HLA-DRB1*08:10, HLA-DRB1*08:12, HLA-DRB1*08:22,HLA-DRB1*08:40, HLA-DRB1*10:02,HLA-DRB1*01:02, HLA-DRB1*01:04, HLA-DRB1*01:05, HLA-DRB1*01:06, HLA-DRB1*01:07, HLA-DRB1*01:08,HLA-DRB1*01:09, HLA-DRB1*01:10, HLA-DRB1*01:12, HLA-DRB1*01:18, HLA-DRB1*01:19, HLA-DRB1*01:20,HLA-DRB1*01:21, HLA-DRB1*01:22, HLA-DRB1*01:23, HLA-DRB1*01:25, HLA-DRB1*01:26, HLA-DRB1*01:27,HLA-DRB1*01:28, HLA-DRB1*01:30, HLA-DRB1*01:31, HLA-DRB1*01:32, HLA-DRB1*08:06,HLA-DRB1*11:42,  HLA-DRB1*11:57, HLA-DRB1*12:12, HLA-DRB1*13:75, HLA-DRB1*13:88,HLA-DRB1*14:06, HLA-DRB1*14:20,HLA-DRB1*14:29, HLA-DRB1*14:31, HLA-DRB1*14:65, HLA-DRB1*14:74, HLA-DRB1*14:78, HLA-DRB1*14:83 |
| 10 | YTGLTQHGK | 44-52 | 14 | HLA-DRB1*01:01, HLA-DRB1*01:05, HLA-DRB1*01:07, HLA-DRB1*01:08,HLA-DRB1*01:12, HLA-DRB1*01:22,HLA-DRB1*01:25, HLA-DRB1*01:27, HLA-DRB1*01:28, HLA-DRB1*01:30, HLA-DRB1*01:31, HLA-DRB1*01:32,HLA-DRB1*14:13, HLA-DRB1*14:52, |
| 11 | WYTGLTQHG | 43-51 | 17 | HLA-DRB1*01:01, HLA-DRB1*01:05, HLA-DRB1*01:07, HLA-DRB1*01:08,HLA-DRB1*01:10, HLA-DRB1*01:12,HLA-DRB1*01:19, HLA-DRB1*01:22, HLA-DRB1*01:23, HLA-DRB1*01:25, HLA-DRB1*01:27, HLA-DRB1*01:28, HLA-DRB1*01:30, HLA-DRB1*01:31,HLA-DRB1*01:32, HLA-DRB1*13:88 |
| 12 | YFLRYSGAI | 327-335 | 35 | HLA-DRB1*01:01, HLA-DRB1*08:40, HLA-DRB1*01:05, HLA-DRB1*01:07,HLA-DRB1*01:08, HLA-DRB1*01:09, HLA-DRB1*01:10, HLA-DRB1*01:12, HLA-DRB1*01:13,HLA-DRB1*01:19, HLA-DRB1*01:22, HLA-DRB1*01:25, HLA-DRB1*01:27, HLA-DRB1*01:28,HLA-DRB1*01:30, HLA-DRB1*01:31,HLA-DRB1*01:32, HLA-DRB1*08:25,HLA-DRB1*11:93,HLA-DRB1*13:100, HLA-DRB1*13:46, HLA-DRB1*13:88, HLA-DRB1*13:89, HLA-DRB1*14:69,HLA-DRB1*15:02, HLA-DRB1*15:08, HLA-DRB1*15:11, HLA-DRB1*15:14, HLA-DRB1*15:19,HLA-DRB1*15:26, HLA-DRB1*15:31, HLA-DRB1*15:38, HLA-DRB1*15:39, HLA-DRB1*15:44, HLA-DRB1*15:47 |
| 13 | YYTGTGPEA | 101-109 | 19 | HLA-DRB1*01:01, HLA-DRB1*01:02, HLA-DRB1*01:05, HLA-DRB1*01:06,HLA-DRB1*01:07, HLA-DRB1*01:08,HLA-DRB1*01:12, HLA-DRB1*01:19, HLA-DRB1*01:20, HLA-DRB1*01:21, HLA-DRB1*01:22, HLA-DRB1*01:23,HLA-DRB1*01:25, HLA-DRB1*01:26, HLA-DRB1*01:27, HLA-DRB1*01:28, HLA-DRB1*01:30, HLA-DRB1*01:31,HLA-DRB1*01:32 |
| 14 | YFYYTGTGP | 99-107 | 12 | HLA-DRB1*01:01, HLA-DRB1*01:05, HLA-DRB1*01:07, HLA-DRB1*01:08,HLA-DRB1*01:12, HLA-DRB1*01:19,HLA-DRB1*01:22, HLA-DRB1*01:25, HLA-DRB1*01:27, HLA-DRB1*01:30, HLA-DRB1*01:31, HLA-DRB1*01:32 |
| 15 | LYLDLLNRL | 214-222 | 160 | HLA-DRB1*01:02,HLA-DRB1*04:12,HLA-DRB1*03:54, HLA-DRB1*03:55, HLA-DRB1*04:55, HLA-DRB1*01:04,HLA-DRB1*04:12,HLA-DRB1*03:52, HLA-DRB1*03:54, HLA-DRB1*03:55, HLA-DRB1*01:06,HLA-DRB1*04:67,HLA-DRB1*08:06, HLA-DRB1*08:10, HLA-DRB1*08:12, HLA-DRB1*01:16,HLA-DRB1*08:22,HLA-DRB1*08:37, HLA-DRB1*11:03, HLA-DRB1*01:17, HLA-DRB1*01:20,HLA-DRB1*01:26,HLA-DRB1*01:28, HLA-DRB1*03:01, HLA-DRB1*03:04, HLA-DRB1*03:07,HLA-DRB1*03:08,HLA-DRB1*03:10, HLA-DRB1*03:11, HLA-DRB1*03:13, HLA-DRB1*03:15,HLA-DRB1*03:18,HLA-DRB1*03:21, HLA-DRB1*03:22, HLA-DRB1*03:23, HLA-DRB1*03:24,HLA-DRB1*03:25,HLA-DRB1*03:28, HLA-DRB1*03:30, HLA-DRB1*03:32, HLA-DRB1*03:33,HLA-DRB1*03:34,HLA-DRB1*03:36, HLA-DRB1*03:37, HLA-DRB1*03:39, HLA-DRB1*03:43,HLA-DRB1*03:44,HLA-DRB1*03:45,HLA-DRB1*03:46, HLA-DRB1*03:47, HLA-DRB1*03:48,HLA-DRB1*03:50,HLA-DRB1*03:51,HLA-DRB1*03:52, HLA-DRB1*03:54, HLA-DRB1*03:55,HLA-DRB1*11:07,HLA-DRB1*11:13,HLA-DRB1*11:18, HLA-DRB1*11:21, HLA-DRB1*11:34,HLA-DRB1*11:36,HLA-DRB1*11:42,HLA-DRB1*11:59, HLA-DRB1*11:63, HLA-DRB1*11:70,HLA-DRB1*11:76,HLA-DRB1*11:83,HLA-DRB1*12:04, HLA-DRB1*11:84, HLA-DRB1*11:85,HLA-DRB1*11:92,HLA-DRB1*12:01,HLA-DRB1*12:03, HLA-DRB1*12:05, HLA-DRB1*12:06,HLA-DRB1*12:07,HLA-DRB1*12:08,HLA-DRB1*12:09, HLA-DRB1*12:10, HLA-DRB1*12:11,HLA-DRB1*12:14,HLA-DRB1*12:17,HLA-DRB1*12:20, HLA-DRB1*12:22, HLA-DRB1*13:03,HLA-DRB1*13:04,HLA-DRB1*13:06,HLA-DRB1*13:09, HLA-DRB1*13:10, HLA-DRB1*13:100,HLA-DRB1*13:101,HLA-DRB1*13:15,HLA-DRB1*13:20,HLA-DRB1*13:21,HLA-DRB1*13:24,HLA-DRB1*13:27,HLA-DRB1*13:30,HLA-DRB1*13:32, HLA-DRB1*13:33, HLA-DRB1*13:43,HLA-DRB1*13:44,HLA-DRB1*13:46,HLA-DRB1*13:48, HLA-DRB1*13:49, HLA-DRB1*13:54,HLA-DRB1*13:57,HLA-DRB1*13:58,HLA-DRB1*13:61, HLA-DRB1*13:66, HLA-DRB1*13:75,HLA-DRB1*13:77,HLA-DRB1*13:78,HLA-DRB1*13:81, HLA-DRB1*13:88, HLA-DRB1*13:89,HLA-DRB1*13:90,HLA-DRB1*13:93,HLA-DRB1*13:94, HLA-DRB1*13:95, HLA-DRB1*14:01,HLA-DRB1*14:06,HLA-DRB1*14:13,HLA-DRB1*14:17, HLA-DRB1*14:20, HLA-DRB1*14:21,HLA-DRB1*14:26,HLA-DRB1*14:29,HLA-DRB1*14:31, HLA-DRB1*14:32, HLA-DRB1*14:33,HLA-DRB1*14:35,DHLA-RB1*14:41,HLA-DRB1*14:48, HLA-DRB1*14:52, HLA-DRB1*14:53,HLA-DRB1*14:54,HLA-DRB1*14:55,HLA-DRB1*14:58, HLA-DRB1*14:60, HLA-DRB1*14:62,HLA-DRB1*14:63,HLA-DRB1*14:65,HLA-DRB1*14:74 HLA-DRB1*14:75, HLA-DRB1*14:76,HLA-DRB1*14:78,HLA-DRB1*14:79,HLA-DRB1*14:80, HLA-DRB1*14:82, HLA-DRB1*14:83,HLA-DRB1*14:85,HLA-DRB1*14:86,HLA-DRB1*14:87, HLA-DRB1*14:88, HLA-DRB1*14:90,HLA-DRB1*14:97,HLA-DRB1*15:12,HLA-DRB1*15:23, HLA-DRB1*15:49 |
| 16 | IKQLAPRWY | 91-99 | 154 | HLA-DRB1*01:02,HLA-DRB1*08:31,HLA-DRB1*08:32,HLA-DRB1*08:37, HLA-DRB1*08:40, HLA-DRB1*09:06,HLA-DRB1*01:04,HLA-DRB1*10:02,HLA-DRB1*11:04, HLA-DRB1*01:06, HLA-DRB1*01:10,HLA-DRB1*01:18,HLA-DRB1*01:20,HLA-DRB1*01:23, HLA-DRB1*01:26, HLA-DRB1*08:05,HLA-DRB1*08:06,HLA-DRB1*08:10,HLA-DRB1*08:12, HLA-DRB1*08:18, HLA-DRB1*08:22,HLA-DRB1*08:25,HLA-DRB1*11:06,HLA-DRB1*11:08, HLA-DRB1*11:13, HLA-DRB1*11:18,HLA-DRB1*11:34,HLA-DRB1*11:35,HLA-DRB1*11:36, HLA-DRB1*11:38,  HLA-DRB1*11:42,HLA-DRB1*11:43,HLA-DRB1*11:44,HLA-DRB1*11:46, HLA-DRB1*11:47, HLA-DRB1*11:50,HLA-DRB1*11:56,HLA-DRB1*11:57,HLA-DRB1*11:58, HLA-DRB1*11:60, HLA-DRB1*11:72,HLA-DRB1*11:77,HLA-DRB1*11:78,HLA-DRB1*11:79, HLA-DRB1*11:83, HLA-DRB1*11:84,HLA-DRB1*11:88,HLA-DRB1*11:92,HLA-DRB1*12:01, HLA-DRB1*12:02, HLA-DRB1*12:03,HLA-DRB1*12:04,HLA-DRB1*12:05,HLA-DRB1*12:06, HLA-DRB1*12:07, HLA-DRB1*12:08,HLA-DRB1*12:09,HLA-DRB1*12:10,HLA-DRB1*12:11, HLA-DRB1*12:12, HLA-DRB1*12:13,HLA-DRB1*12:14,HLA-DRB1*12:17,HLA-DRB1*12:15, HLA-DRB1*12:18, HLA-DRB1*12:19,HLA-DRB1*12:20,HLA-DRB1*12:21,HLA-DRB1*12:23, HLA-DRB1*13:03, HLA-DRB1*13:04,HLA-DRB1*13:06,HLA-DRB1*13:09,HLA-DRB1*13:10, HLA-DRB1*13:100, HLA-DRB1*13:101,HLA-DRB1*13:11,HLA-DRB1*13:12,HLA-DRB1*13:20, HLA-DRB1*13:21, HLA-DRB1*13:30,HLA-DRB1*13:42, HLA-DRB1*13:43,HLA-DRB1*13:44, HLA-DRB1*13:46, HLA-DRB1*13:49,HLA-DRB1*13:56, HLA-DRB1*13:58,HLA-DRB1*13:60, HLA-DRB1*13:66,  HLA-DRB1*13:75,HLA-DRB1*13:77,HLA-DRB1*13:78,HLA-DRB1*13:81, HLA-DRB1*13:86, HLA-DRB1*13:88,HLA-DRB1*13:89,HLA-DRB1*13:90,HLA-DRB1*13:93, HLA-DRB1*13:94, HLA-DRB1*14:02,HLA-DRB1*14:06,HLA-DRB1*13:95,HLA-DRB1*14:12, HLA-DRB1*14:13, HLA-DRB1*14:17,HLA-DRB1*14:19,HLA-DRB1*14:20,HLA-DRB1*14:21, HLA-DRB1*14:29, HLA-DRB1*14:31,HLA-DRB1*14:32,HLA-DRB1*14:33,HLA-DRB1*14:34, HLA-DRB1*14:41, HLA-DRB1*14:48,HLA-DRB1*14:49,HLA-DRB1*14:52,HLA-DRB1*14:53, HLA-DRB1*14:55,  HLA-DRB1*14:62,HLA-DRB1*14:65,HLA-DRB1*14:69,HLA-DRB1*14:74, HLA-DRB1*14:78, HLA-DRB1*14:80,HLA-DRB1*14:81,HLA-DRB1*14:83,HLA-DRB1*14:84, HLA-DRB1*14:85, HLA-DRB1*14:94,HLA-DRB1*15:01,HLA-DRB1*15:03,HLA-DRB1*15:09, HLA-DRB1*15:12, HLA-DRB1*15:13,HLA-DRB1*15:16,HLA-DRB1*15:18,HLA-DRB1*15:20, HLA-DRB1*15:22, HLA-DRB1*15:23,HLA-DRB1*15:24,HLA-DRB1*15:28,HLA-DRB1*15:32, HLA-DRB1*15:33, HLA-DRB1*15:35,HLA-DRB1*15:36,HLA-DRB1*15:40,HLA-DRB1*15:41, HLA-DRB1*15:42,  HLA-DRB1*15:43,HLA-DRB1*15:45, HLA-DRB1*15:46, HLA-DRB1*15:49 |
| 17 | FLRYSGAIK | 328-336 | 135 | HLA-DRB1*01:02,HLA-DRB1*08:22,HLA-DRB1*08:28,HLA-DRB1*08:31, HLA-DRB1*09:03, HLA-DRB1*11:01,HLA-DRB1*01:04,HLA-DRB1*11:05,HLA-DRB1*01:06, HLA-DRB1*01:20, HLA-DRB1*01:23,HLA-DRB1*01:26,HLA-DRB1*08:05,HLA-DRB1*08:06, HLA-DRB1*08:13, HLA-DRB1*11:06,HLA-DRB1*11:08,HLA-DRB1*11:09,HLA-DRB1*11:10, HLA-DRB1*11:12, HLA-DRB1*11:15,HLA-DRB1*11:19,HLA-DRB1*11:24,HLA-DRB1*11:27, HLA-DRB1*11:28, HLA-DRB1*11:29,HLA-DRB1*11:30,HLA-DRB1*11:31,HLA-DRB1*11:33, HLA-DRB1*11:39, HLA-DRB1*11:47,HLA-DRB1*11:49,HLA-DRB1*11:50,HLA-DRB1*11:51, HLA-DRB1*11:54, HLA-DRB1*11:56,HLA-DRB1*11:57,HLA-DRB1*11:61,HLA-DRB1*11:62, HLA-DRB1*11:64, HLA-DRB1*11:67,HLA-DRB1*11:74,HLA-DRB1*11:75,HLA-DRB1*11:79, HLA-DRB1*11:80, HLA-DRB1*11:81,HLA-DRB1*11:87,HLA-DRB1*11:88,HLA-DRB1*11:90, HLA-DRB1*11:91, HLA-DRB1*11:92,HLA-DRB1*11:93,HLA-DRB1*11:94,HLA-DRB1*11:95, HLA-DRB1*11:96, HLA-DRB1*12:08,HLA-DRB1*12:16,HLA-DRB1*12:19,HLA-DRB1*12:20, HLA-DRB1*13:03, HLA-DRB1*13:05,HLA-DRB1*13:100,HLA-DRB1*13:12,HLA-DRB1*13:14, HLA-DRB1*13:21, HLA-DRB1*13:30,HLA-DRB1*13:46,HLA-DRB1*13:49,HLA-DRB1*13:50, HLA-DRB1*13:56, HLA-DRB1*13:58,HLA-DRB1*13:60,HLA-DRB1*13:62,HLA-DRB1*13:66, HLA-DRB1*13:75, HLA-DRB1*13:81,HLA-DRB1*13:82,HLA-DRB1*13:86,HLA-DRB1*13:88, HLA-DRB1*13:90, HLA-DRB1*13:95,HLA-DRB1*14:02,HLA-DRB1*14:03,HLA-DRB1*14:07, HLA-DRB1*14:09, HLA-DRB1*14:13,HLA-DRB1*14:19,HLA-DRB1*14:22,HLA-DRB1*14:25, HLA-DRB1*14:29,  HLA-DRB1*14:30, HLA-DRB1*14:32,HLA-DRB1*14:34,HLA-DRB1*14:40, HLA-DRB1*14:41, HLA-DRB1*14:46,HLA-DRB1*14:47, HLA-DRB1*14:48, HLA-DRB1*14:49, HLA-DRB1*14:65, HLA-DRB1*14:69, HLA-DRB1*14:73,HLA-DRB1*14:74, HLA-DRB1*14:77, HLA-DRB1*14:78, HLA-DRB1*14:81, HLA-DRB1*14:84, HLA-DRB1*14:85,HLA-DRB1*14:94, HLA-DRB1*15:02, HLA-DRB1*15:03, HLA-DRB1*15:08, HLA-DRB1*15:12,HLA-DRB1*15:26, HLA-DRB1*15:14, HLA-DRB1*15:18, HLA-DRB1*15:19, HLA-DRB1*15:21, HLA-DRB1*15:23,HLA-DRB1*15:27,  HLA-DRB1*15:31, HLA-DRB1*15:34, HLA-DRB1*15:38, HLA-DRB1*15:39,HLA-DRB1*15:44, HLA-DRB1*15:47, HLA-DRB1*15:49, HLA-DRB1*16:02, HLA-DRB1*16:05,HLA-DRB1*16:07, HLA-DRB1*16:10, HLA-DRB1*16:12, HLA-DRB1*16:14, HLA-DRB1*16:15, HLA-DRB1*16:16 |
| 18 | LAPTASAFM | 299-307 | 15 | HLA-DRB1*01:02, HLA-DRB1*13:81, HLA-DRB1*01:04, HLA-DRB1*01:06,HLA-DRB1*01:10, HLA-DRB1*01:16, HLA-DRB1*01:20, HLA-DRB1*01:26, HLA-DRB1*13:88,HLA-DRB1*01:14, HLA-DRB1*01:15, HLA-DRB1*11:13, HLA-DRB1*13:58, HLA-DRB1*13:89, HLA-DRB1*14:74 |
| 19 | WLELLEQNI | 347-355 | 10 | HLA-DRB1*01:02,HLA-DRB1*01:04,HLA-DRB1*01:06,HLA-DRB1*01:14, HLA-DRB1*01:18, HLA-DRB1*01:20,HLA-DRB1*01:23,HLA-DRB1*01:26,HLA-DRB1*13:58, HLA-DRB1*14:65 |
| 20 | TQFAPGTKL | 148-156 | 32 | HLA-DRB1*01:02,HLA-DRB1*08:10,HLA-DRB1*08:12,HLA-DRB1*09:03, HLA-DRB1*09:08, HLA-DRB1*01:06,HLA-DRB1*01:09,HLA-DRB1*01:10,HLA-DRB1*01:13, HLA-DRB1*01:23, HLA-DRB1*01:26,HLA-DRB1*07:06,HLA-DRB1*13:03,HLA-DRB1*13:10, HLA-DRB1*13:66, HLA-DRB1*13:75,HLA-DRB1*13:88,HLA-DRB1*13:90,HLA-DRB1*13:94, HLA-DRB1*13:95, HLA-DRB1*14:13,HLA-DRB1*14:78,HLA-DRB1*08:12,HLA-DRB1*11:92, HLA-DRB1*13:101, HLA-DRB1*13:21,HLA-DRB1*13:30,HLA-DRB1*13:58,HLA-DRB1*13:81, HLA-DRB1*13:89, HLA-DRB1*13:94, HLA-DRB1*14:84 |
| 21 | ITKKDAAAA | 238-246 | 20 | HLA-DRB1*01:02,HLA-DRB1*08:22,HLA-DRB1*08:40,HLA-DRB1*01:06, HLA-DRB1*01:09, HLA-DRB1*01:10,HLA-DRB1*01:15,HLA-DRB1*01:20,HLA-DRB1*01:23, HLA-DRB1*01:26, HLA-DRB1*08:06,HLA-DRB1*08:10,HLA-DRB1*08:12,HLA-DRB1*13:58, HLA-DRB1*13:81, HLA-DRB1*13:88,HLA-DRB1*13:89,HLA-DRB1*13:89,HLA-DRB1*14:21, HLA-DRB1*14:74 |
| 22 | VYFLRYSGA | 326-334 | 57 | HLA-DRB1*01:02,HLA-DRB1*08:31,HLA-DRB1*08:40,HLA-DRB1*11:04, HLA-DRB1*11:05, HLA-DRB1*01:18,HLA-DRB1*01:20,HLA-DRB1*01:23, HLA-DRB1*01:26, HLA-DRB1*08:05, HLA-DRB1*08:06,HLA-DRB1*08:10,HLA-DRB1*08:12,HLA-DRB1*08:18, HLA-DRB1*11:06, HLA-DRB1*11:08,HLA-DRB1*11:13,HLA-DRB1*11:34,HLA-DRB1*11:35, HLA-DRB1*11:38, HLA-DRB1*11:42,HLA-DRB1*11:43,HLA-DRB1*11:44,HLA-DRB1*11:46, HLA-DRB1*11:47, HLA-DRB1*11:57,HLA-DRB1*11:58,HLA-DRB1*11:60,HLA-DRB1*11:77, HLA-DRB1*11:78, HLA-DRB1*11:92,HLA-DRB1*12:02,HLA-DRB1*12:03,HLA-DRB1*12:04, HLA-DRB1*12:09, HLA-DRB1*12:12,HLA-DRB1*12:13,HLA-DRB1*12:18,HLA-DRB1*12:19, HLA-DRB1*12:20, HLA-DRB1*12:15, HLA-DRB1*12:21, HLA-DRB1*12:23,HLA-DRB1*13:11, HLA-DRB1*13:21, HLA-DRB1*13:44, HLA-DRB1*13:49, HLA-DRB1*13:56,HLA-DRB1*13:58, HLA-DRB1*13:81, HLA-DRB1*14:17, HLA-DRB1*14:31, HLA-DRB1*14:52,HLA-DRB1*14:53, HLA-DRB1*14:74, HLA-DRB1*15:23, HLA-DRB1*15:49 |
| 23 | IGAVGGDLL | 206-214 | 8 | HLA-DRB1*01:02,HLA-DRB1*01:06,HLA-DRB1*01:09,HLA-DRB1*01:20, HLA-DRB1*01:23, HLA-DRB1*01:26,HLA-DRB1*13:81,HLA-DRB1*13:89 |
| 24 | LLYLDLLNR | 213-221 | 110 | HLA-DRB1*01:02, HLA-DRB1*08:12, HLA-DRB1*08:18, HLA-DRB1*08:22,HLA-DRB1*08:28, HLA-DRB1*08:31,HLA-DRB1*01:20, HLA-DRB1*08:32, HLA-DRB1*08:37, HLA-DRB1*08:40, HLA-DRB1*10:02, HLA-DRB1*11:04,HLA-DRB1*01:23, HLA-DRB1*01:26, HLA-DRB1*04:10, HLA-DRB1*08:05, HLA-DRB1*08:06, HLA-DRB1*08:10, HLA-DRB1*11:06, HLA-DRB1*11:08, HLA-DRB1*11:13, HLA-DRB1*11:18, HLA-DRB1*11:34, HLA-DRB1*11:35, HLA-DRB1*11:38, HLA-DRB1*11:42, HLA-DRB1*11:43, HLA-DRB1*11:44, HLA-DRB1*11:46, HLA-DRB1*11:47, HLA-DRB1*11:50, HLA-DRB1*11:56, HLA-DRB1*11:57, HLA-DRB1*11:58, HLA-DRB1*11:60, HLA-DRB1*11:67, HLA-DRB1*11:72, HLA-DRB1*11:77, HLA-DRB1*11:78, HLA-DRB1*11:79, HLA-DRB1*11:83, HLA-DRB1*11:84, HLA-DRB1*11:88, HLA-DRB1*11:92, HLA-DRB1*12:01, HLA-DRB1*12:02, HLA-DRB1*12:03, HLA-DRB1*12:04, HLA-DRB1*12:05,HLA-DRB1*12:06, HLA-DRB1*12:07, HLA-DRB1*12:08, HLA-DRB1*12:09, HLA-DRB1*12:10,HLA-DRB1*12:11, HLA-DRB1*12:12, HLA-DRB1*12:13, HLA-DRB1*12:14, HLA-DRB1*12:15,HLA-DRB1*12:17, HLA-DRB1*12:18, HLA-DRB1*12:19, HLA-DRB1*12:20, HLA-DRB1*12:21,HLA-DRB1*12:23, HLA-DRB1*13:06, HLA-DRB1*13:10,HLA-DRB1*13:101,HLA-DRB1*13:11,HLA-DRB1*13:12, HLA-DRB1*13:21, HLA-DRB1*13:42, HLA-DRB1*13:44, HLA-DRB1*13:49,HLA-DRB1*13:56, HLA-DRB1*13:58, HLA-DRB1*13:60, HLA-DRB1*13:66, HLA-DRB1*13:81,HLA-DRB1*13:86, HLA-DRB1*13:89, HLA-DRB1*13:94, HLA-DRB1*14:02, HLA-DRB1*14:06,HLA-DRB1*14:12, HLA-DRB1*14:13, HLA-DRB1*14:13, HLA-DRB1*14:17, HLA-DRB1*14:20,HLA-DRB1*14:21, HLA-DRB1*14:29, HLA-DRB1*14:30, HLA-DRB1*14:31, HLA-DRB1*14:33,HLA-DRB1*14:34, HLA-DRB1*14:41, HLA-DRB1*14:49, HLA-DRB1*14:52, HLA-DRB1*14:53,HLA-DRB1*14:55, HLA-DRB1*14:69, HLA-DRB1*14:74, HLA-DRB1*14:78, HLA-DRB1*14:80,HLA-DRB1*14:81, HLA-DRB1*14:83, HLA-DRB1*14:84, HLA-DRB1*14:85, HLA-DRB1*14:94, HLA-DRB1*15:23 |
| 25 | VQGSITQRT | 388-396 | 11 | HLA-DRB1*01:02, HLA-DRB1*01:06, HLA-DRB1*01:23, HLA-DRB1*01:26,HLA-DRB1*08:10, HLA-DRB1*13:58, HLA-DRB1*13:81, HLA-DRB1*13:89, HLA-DRB1*13:94,HLA-DRB1*14:19, HLA-DRB1*14:52 |
| 26 | INTGNGIKQ | 85-93 | 11 | HLA-DRB1*01:02,HLA-DRB1*01:06, HLA-DRB1*13:58, HLA-DRB1*13:89, HLA-DRB1*13:94, HLA-DRB1*01:26, HLA-DRB1*08:06, HLA-DRB1*11:35, HLA-DRB1*13:81,HLA-DRB1*13:88, HLA-DRB1*14:21 |
| 27 | VKQSQPKVI | 230-238 | 94 | HLA-DRB1*01:02, HLA-DRB1*11:02, HLA-DRB1*11:03, HLA-DRB1*01:04, HLA-DRB1*01:06, HLA-DRB1*01:09,HLA-DRB1*01:15, HLA-DRB1*01:16, HLA-DRB1*07:06, HLA-DRB1*08:10, HLA-DRB1*08:40, HLA-DRB1*11:18,HLA-DRB1*11:16, HLA-DRB1*11:21, HLA-DRB1*11:34, HLA-DRB1*11:36, HLA-DRB1*11:63, HLA-DRB1*11:65, HLA-DRB1*11:70, HLA-DRB1*11:76, HLA-DRB1*13:01, HLA-DRB1*13:03, HLA-DRB1*13:06, HLA-DRB1*13:09,HLA-DRB1*13:10, HLA-DRB1*13:101,HLA-DRB1*13:12, HLA-DRB1*13:15,HLA-DRB1*13:19,HLA-DRB1*13:20, HLA-DRB1*13:22,HLA-DRB1*13:24, HLA-DRB1*13:27, HLA-DRB1*13:32, HLA-DRB1*13:33, HLA-DRB1*13:35, HLA-DRB1*13:41, HLA-DRB1*13:43, HLA-DRB1*13:44,HLA-DRB1*13:48, HLA-DRB1*13:51, HLA-DRB1*13:52, HLA-DRB1*13:53, HLA-DRB1*13:57,HLA-DRB1*13:58, HLA-DRB1*13:59, HLA-DRB1*13:64, HLA-DRB1*13:66, HLA-DRB1*13:68,HLA-DRB1*13:69, HLA-DRB1*13:71, HLA-DRB1*13:75, HLA-DRB1*13:78, HLA-DRB1*13:79,HLA-DRB1*13:80, HLA-DRB1*13:81, HLA-DRB1*13:83, HLA-DRB1*13:87, HLA-DRB1*13:88,HLA-DRB1*13:90, HLA-DRB1*13:91, HLA-DRB1*13:92, HLA-DRB1*13:93, HLA-DRB1*13:94,HLA-DRB1*13:95, HLA-DRB1*13:98, HLA-DRB1*14:13, HLA-DRB1*14:17, HLA-DRB1*14:21,HLA-DRB1*14:29, HLA-DRB1*14:37, HLA-DRB1*14:74, HLA-DRB1*14:78, HLA-DRB1*14:80,HLA-DRB1*01:10, HLA-DRB1*01:26, HLA-DRB1*08:12, HLA-DRB1*11:16, HLA-DRB1*11:42, HLA-DRB1*11:57, HLA-DRB1*11:59, HLA-DRB1*11:92, HLA-DRB1*12:08, HLA-DRB1*12:12, HLA-DRB1*13:01, HLA-DRB1*13:04, HLA-DRB1*13:30, HLA-DRB1*13:77, HLA-DRB1*13:89,HLA-DRB1*14:06, HLA-DRB1*14:20, HLA-DRB1*14:52, HLA-DRB1*14:65, HLA-DRB1*14:83 |
| 28 | MVQAFGLRG | 262-270 | 47 | HLA-DRB1*01:02, HLA-DRB1*01:26, HLA-DRB1*04:10, HLA-DRB1*08:06,HLA-DRB1*08:06, HLA-DRB1*08:10, HLA-DRB1*08:12, HLA-DRB1*08:22, HLA-DRB1*08:31,HLA-DRB1*09:06, HLA-DRB1*11:04, HLA-DRB1*11:34, HLA-DRB1*11:35, HLA-DRB1*11:38, HLA-DRB1*11:42, HLA-DRB1*11:43, HLA-DRB1*11:44, HLA-DRB1*11:46, HLA-DRB1*11:50, HLA-DRB1*11:56, HLA-DRB1*11:58, HLA-DRB1*11:60, HLA-DRB1*11:77, HLA-DRB1*11:78, HLA-DRB1*11:85, HLA-DRB1*11:88, HLA-DRB1*11:92, HLA-DRB1*12:08, HLA-DRB1*13:11, HLA-DRB1*13:21, HLA-DRB1*13:42, HLA-DRB1*13:44, HLA-DRB1*13:58, HLA-DRB1*13:75,HLA-DRB1*13:81, HLA-DRB1*13:88, HLA-DRB1*13:89, HLA-DRB1*14:06, HLA-DRB1*14:13,HLA-DRB1*14:17, HLA-DRB1*14:20, HLA-DRB1*14:21, HLA-DRB1*14:29, HLA-DRB1*14:52,HLA-DRB1*14:80, HLA-DRB1*14:83, HLA-DRB1*14:84 |
| 29 | MRHKRTSTK | 250-258 | 58 | HLA-DRB1*01:02, HLA-DRB1*08:31, HLA-DRB1*08:40, HLA-DRB1*11:04,HLA-DRB1*01:23, HLA-DRB1*01:26,HLA-DRB1*08:04, HLA-DRB1*08:05, HLA-DRB1*08:06, HLA-DRB1*08:10, HLA-DRB1*08:12, HLA-DRB1*08:22,HLA-DRB1*08:28, HLA-DRB1*11:06, HLA-DRB1*11:35, HLA-DRB1*11:38, HLA-DRB1*11:43, HLA-DRB1*11:44,HLA-DRB1*11:46, HLA-DRB1*11:47, HLA-DRB1*11:50, HLA-DRB1*11:56, HLA-DRB1*11:58, HLA-DRB1*11:60,HLA-DRB1*11:67, HLA-DRB1*11:77, HLA-DRB1*11:78, HLA-DRB1*11:83, HLA-DRB1*11:88, HLA-DRB1*11:92,  HLA-DRB1*13:11, HLA-DRB1*13:42, HLA-DRB1*13:49, HLA-DRB1*13:58,HLA-DRB1*13:81, HLA-DRB1*13:85,HLA-DRB1*13:88, HLA-DRB1*13:89, HLA-DRB1*13:94, HLA-DRB1*14:02, HLA-DRB1*14:06, HLA-DRB1*14:12,HLA-DRB1*14:13, HLA-DRB1*14:15, HLA-DRB1*14:19, HLA-DRB1*14:20, HLA-DRB1*14:21, HLA-DRB1*14:29,HLA-DRB1*14:41, HLA-DRB1*14:52, HLA-DRB1*14:73, HLA-DRB1*14:74, HLA-DRB1*14:78, HLA-DRB1*14:80,HLA-DRB1*14:81, HLA-DRB1*14:83, HLA-DRB1*14:84, HLA-DRB1*14:94 |
| 30 | GIKQLAPRW | 90-98 | 2 | HLA-DRB1*01:06, HLA-DRB1*01:10 |
| 31 | YTGTGPEAA | 102-110 | 4 | HLA-DRB1*01:06, HLA-DRB1*01:09, HLA-DRB1*01:10, HLA-DRB1*01:23 |
| 32 | PSVQPGPMI | 400-408 | 1 | HLA-DRB1*01:06 |
| 33 | QIAELAPTA | 295-303 | 7 | HLA-DRB1*01:09, HLA-DRB1*01:10, HLA-DRB1*01:11, HLA-DRB1*01:17,HLA-DRB1*01:18, HLA-DRB1*01:21, HLA-DRB1*01:24 |
| 34 | AAAAKNKMR | 243-251 | 14 | HLA-DRB1*01:23, HLA-DRB1*08:06, HLA-DRB1*08:12, HLA-DRB1*08:22,HLA-DRB1*11:57, HLA-DRB1*11:83,HLA-DRB1*13:58, HLA-DRB1*14:06, HLA-DRB1*14:20, HLA-DRB1*14:29, HLA-DRB1*14:83, HLA-DRB1*13:81, HLA-DRB1*13:89, HLA-DRB1*14:84 |
| 35 | KNKMRHKRT | 247-255 | 39 | HLA-DRB1*01:23, HLA-DRB1*08:06, HLA-DRB1*08:10, HLA-DRB1*08:12,HLA-DRB1*08:31, HLA-DRB1*11:04, HLA-DRB1*11:38, HLA-DRB1*11:50, HLA-DRB1*11:56, HLA-DRB1*11:57, HLA-DRB1*11:77, HLA-DRB1*11:88, HLA-DRB1*11:92, HLA-DRB1*12:20, HLA-DRB1*13:58, HLA-DRB1*13:81, HLA-DRB1*14:06, HLA-DRB1*14:12, HLA-DRB1*14:20,HLA-DRB1*14:29, HLA-DRB1*14:52, HLA-DRB1*14:78, HLA-DRB1*14:83, HLA-DRB1*14:84,HLA-DRB1*14:85, HLA-DRB1*08:22, HLA-DRB1*11:43, HLA-DRB1*11:44, HLA-DRB1*11:46, HLA-DRB1*11:58, HLA-DRB1*11:60, HLA-DRB1*11:78, HLA-DRB1*12:04, HLA-DRB1*12:09, HLA-DRB1*12:21, HLA-DRB1*13:11, HLA-DRB1*13:42, HLA-DRB1*14:13, HLA-DRB1*14:21 |
| 36 | LDLLNRLQA | 216-224 | 78 | HLA-DRB1*03:24, HLA-DRB1*08:31, HLA-DRB1*11:04, HLA-DRB1*08:04,HLA-DRB1*08:05, HLA-DRB1*08:06, HLA-DRB1*08:10, HLA-DRB1*08:12, HLA-DRB1*08:22,HLA-DRB1*08:28, HLA-DRB1*11:06, HLA-DRB1*11:13, HLA-DRB1*11:18, HLA-DRB1*11:25, HLA-DRB1*11:34, HLA-DRB1*11:35, HLA-DRB1*11:38, HLA-DRB1*11:42, HLA-DRB1*11:43, HLA-DRB1*11:44, HLA-DRB1*11:46, HLA-DRB1*11:50, HLA-DRB1*11:56, HLA-DRB1*11:57,HLA-DRB1*11:58, HLA-DRB1*11:60, HLA-DRB1*11:67, HLA-DRB1*11:72, HLA-DRB1*11:77, HLA-DRB1*12:02,  HLA-DRB1*11:78, HLA-DRB1*11:88, HLA-DRB1*11:92, HLA-DRB1*12:01, HLA-DRB1*12:03, HLA-DRB1*12:04, HLA-DRB1*12:05, HLA-DRB1*12:06, HLA-DRB1*12:07,HLA-DRB1*12:08, HLA-DRB1*12:09, HLA-DRB1*12:12, HLA-DRB1*12:10, HLA-DRB1*12:11,HLA-DRB1*12:13, HLA-DRB1*12:14, HLA-DRB1*12:15, HLA-DRB1*12:17, HLA-DRB1*12:18,HLA-DRB1*12:19, HLA-DRB1*12:20, HLA-DRB1*12:23, HLA-DRB1*12:21, HLA-DRB1*13:06,HLA-DRB1*13:09, HLA-DRB1*13:11, HLA-DRB1*13:18, HLA-DRB1*13:42, HLA-DRB1*13:44,HLA-DRB1*13:58,  HLA-DRB1*13:89, HLA-DRB1*14:04, HLA-DRB1*14:06, HLA-DRB1*14:12,HLA-DRB1*14:15, HLA-DRB1*14:17, HLA-DRB1*14:20, HLA-DRB1*14:21, HLA-DRB1*14:29,HLA-DRB1*14:32, HLA-DRB1*14:52, HLA-DRB1*14:55, HLA-DRB1*14:61, HLA-DRB1*14:74,HLA-DRB1*14:78, HLA-DRB1*14:80, HLA-DRB1*14:83, HLA-DRB1*14:84 |
| 37 | FAPGTKLPK | 150-158 | 65 | HLA-DRB1*08:05, HLA-DRB1*08:40, HLA-DRB1*11:04, HLA-DRB1*08:06,HLA-DRB1*08:10, HLA-DRB1*08:12, HLA-DRB1*08:18, HLA-DRB1*08:22, HLA-DRB1*08:31,HLA-DRB1*11:06, HLA-DRB1*11:08, HLA-DRB1*11:18, HLA-DRB1*11:34, HLA-DRB1*11:35, HLA-DRB1*11:38, HLA-DRB1*11:42, HLA-DRB1*11:43, HLA-DRB1*11:44, HLA-DRB1*11:46, HLA-DRB1*11:47, HLA-DRB1*11:57, HLA-DRB1*11:58, HLA-DRB1*11:60, HLA-DRB1*11:77,HLA-DRB1*11:78, HLA-DRB1*11:79, HLA-DRB1*11:92, HLA-DRB1*11:93, HLA-DRB1*13:06, HLA-DRB1*13:10,  HLA-DRB1*13:101, HLA-DRB1*13:11,HLA-DRB1*13:12,HLA-DRB1*13:21,HLA-DRB1*13:42, HLA-DRB1*13:44, HLA-DRB1*13:49, HLA-DRB1*13:56, HLA-DRB1*13:58,HLA-DRB1*13:60, HLA-DRB1*13:66, HLA-DRB1*13:81, HLA-DRB1*13:86, HLA-DRB1*13:88,HLA-DRB1*14:02, HLA-DRB1*14:06, HLA-DRB1*14:09, HLA-DRB1*14:13,HLA-DRB1*14:17, HLA-DRB1*14:19, HLA-DRB1*14:20, HLA-DRB1*14:21, HLA-DRB1*14:29, HLA-DRB1*14:30,HLA-DRB1*14:41, HLA-DRB1*14:47, HLA-DRB1*14:49, HLA-DRB1*14:52, HLA-DRB1*14:74,HLA-DRB1*14:78,  HLA-DRB1*14:80, HLA-DRB1*14:81, HLA-DRB1*14:83, HLA-DRB1*14:84, HLA-DRB1*14:94 |
| 38 | PVYFLRYSG | 325-333 | 43 | HLA-DRB1*08:05, HLA-DRB1*08:37, HLA-DRB1*11:04, HLA-DRB1*11:18, HLA-DRB1*11:34, HLA-DRB1*11:35, HLA-DRB1*11:38, HLA-DRB1*11:43, HLA-DRB1*11:44, HLA-DRB1*11:46, HLA-DRB1*11:57, HLA-DRB1*11:58, HLA-DRB1*11:77, HLA-DRB1*11:78, HLA-DRB1*12:01, HLA-DRB1*12:03, HLA-DRB1*12:05, HLA-DRB1*12:06, HLA-DRB1*12:07,HLA-DRB1*12:10, HLA-DRB1*12:11, HLA-DRB1*12:16, HLA-DRB1*12:17, HLA-DRB1*13:11,HLA-DRB1*13:42, HLA-DRB1*13:60, HLA-DRB1*13:89, HLA-DRB1*14:31, HLA-DRB1*14:80,HLA-DRB1*08:18, HLA-DRB1*08:18, HLA-DRB1*11:18, HLA-DRB1*12:14, HLA-DRB1*13:06,HLA-DRB1*13:44, HLA-DRB1*13:58, HLA-DRB1*14:06, HLA-DRB1*14:13, HLA-DRB1*14:17,HLA-DRB1*14:21, HLA-DRB1*14:65, HLA-DRB1*14:83 |
| 39 | ITQRTRTRP | 392-400 | 21 | HLA-DRB1*08:06, HLA-DRB1*08:10, HLA-DRB1*08:12, HLA-DRB1*08:22,HLA-DRB1*08:31, HLA-DRB1*11:06, HLA-DRB1*11:47, HLA-DRB1*11:50, HLA-DRB1*11:56, HLA-DRB1*11:83, HLA-DRB1*11:88, HLA-DRB1*13:58, HLA-DRB1*13:81, HLA-DRB1*13:89,HLA-DRB1*14:12, HLA-DRB1*14:13, HLA-DRB1*14:21, HLA-DRB1*14:52, HLA-DRB1*14:78,HLA-DRB1*14:84, HLA-DRB1*14:85 |
| 40 | AKNKMRHKR | 246-254 | 2 | HLA-DRB1*08:06, HLA-DRB1*08:12 |
| 41 | MGMSQFKLT | 307-315 | 39 | HLA-DRB1*08:06, HLA-DRB1*08:22, HLA-DRB1*12:01, HLA-DRB1*12:02,HLA-DRB1*12:05, HLA-DRB1*12:06, HLA-DRB1*12:07, HLA-DRB1*12:08, HLA-DRB1*12:10,HLAD-RB1*12:11, HLA-DRB1*12:12, HLA-DRB1*12:13,HLA-DRB1*12:14, HLA-DRB1*12:15, HLA-DRB1*12:17, HLA-DRB1*12:18, HLA-DRB1*12:19, HLA-DRB1*12:20,HLA-DRB1*12:23, HLA-DRB1*13:58, HLA-DRB1*08:12, HLA-DRB1*11:42, HLA-DRB1*11:57, HLA-DRB1*11:60, HLA-DRB1*12:04, HLA-DRB1*12:09, HLA-DRB1*13:58, HLA-DRB1*13:81, HLA-DRB1*13:94,HLA-DRB1*14:06,  HLA-DRB1*14:20, HLA-DRB1*14:21, HLA-DRB1*14:29, HLA-DRB1*14:31,HLA-DRB1*14:52, HLA-DRB1*14:65, HLA-DRB1*14:74, HLA-DRB1*14:78, HLA-DRB1*14:83 |
| 42 | KVITKKDAA | 236-244 | 6 | HLA-DRB1*08:06, HLA-DRB1*08:10, HLA-DRB1*08:12, HLA-DRB1*08:22,HLA-DRB1*13:58, HLA-DRB1*14:78 |
| 43 | WRRQDRKIN | 78-86 | 3 | HLA-DRB1*08:06, HLA-DRB1*08:22, HLA-DRB1*14:52 |
| 44 | KVKQSQPKV | 229-237 | 1 | HLA-DRB1*08:06 |
| 45 | LTQHGKVPL | 47-55 | 20 | HLA-DRB1*08:06, HLA-DRB1*08:10, HLA-DRB1*08:12, HLA-DRB1*11:34, HLA-DRB1*11:42, HLA-DRB1*11:57, HLA-DRB1*12:12, HLA-DRB1*13:44, HLA-DRB1*13:58,HLA-DRB1*13:66, HLA-DRB1*13:75, HLA-DRB1*13:81,HLA-DRB1*13:88, HLA-DRB1*13:89, HLA-DRB1*13:94, HLA-DRB1*14:17, HLA-DRB1*14:21, HLA-DRB1*14:52, HLA-DRB1*14:65, HLA-DRB1*14:74 |
| 46 | GMSQFKLTH | 308-316 | 2 | HLA-DRB1*08:10, HLA-DRB1*08:40 |
| 47 | ITNTNLSRG | 18-26 | 6 | HLA-DRB1*08:10,HLA-DRB1*08:12, HLA-DRB1*13:58, HLA-DRB1*13:81, HLA-DRB1*13:88, HLA-DRB1*13:89 |
| 48 | AAKNKMRHK | 245-253 | 7 | HLA-DRB1*08:10, HLA-DRB1*11:06, HLA-DRB1*11:47, HLA-DRB1*11:50, HLA-DRB1*11:56, HLA-DRB1*11:88, HLA-DRB1*11:83 |
| 49 | VTQFAPGTK | 147-155 | 22 | HLA-DRB1*08:12, HLA-DRB1*08:18, HLA-DRB1*08:31, HLA-DRB1*08:40,HLA-DRB1*11:57, HLA-DRB1*11:92, HLA-DRB1*13:10,HLA-DRB1*13:101,HLA-DRB1*13:12,HLA-DRB1*13:21, HLA-DRB1*13:30, HLA-DRB1*13:49, HLA-DRB1*13:94, HLA-DRB1*14:06,HLA-DRB1*14:13, HLA-DRB1*14:19, HLA-DRB1*14:20, HLA-DRB1*14:52,HLA-DRB1*14:69, HLA-DRB1*14:78, HLA-DRB1*14:80, HLA-DRB1*14:83 |
| 50 | MSQFKLTHQ | 309-317 | 23 | HLA-DRB1*08:18, HLA-DRB1*11:13, HLA-DRB1*11:18, HLA-DRB1*12:01, HLA-DRB1*12:03, HLA-DRB1*12:05, HLA-DRB1*12:06, HLA-DRB1*12:07, HLA-DRB1*12:10,HLA-DRB1*12:12, HLA-DRB1*12:14, HLA-DRB1*12:17, HLA-DRB1*13:06, HLA-DRB1*13:21,HLA-DRB1*13:58, HLA-DRB1*13:77, HLA-DRB1*13:88, HLA-DRB1*13:89,HLA-DRB1*14:13, HLA-DRB1*14:21, HLA-DRB1*14:31, HLA-DRB1*14:74, HLA-DRB1*14:80 |
| 51 | FYYTGTGPE | 100-108 | 1 | DRB1*09:08 |
| 52 | SITQRTRTR | 391-399 | 20 | HLA-DRB1*11:04,HLA-DRB1*11:35,HLA-DRB1*11:38,HLA-DRB1*11:43, HLA-DRB1*11:44, HLA-DRB1*11:46,HLA-DRB1*11:58,HLA-DRB1*11:60,HLA-DRB1*11:77, HLA-DRB1*11:78, HLA-DRB1*12:04,HLA-DRB1*12:09,HLA-DRB1*13:11,HLA-DRB1*13:42, HLA-DRB1*13:58, HLA-DRB1*13:88,HLA-DRB1*14:06,HLA-DRB1*14:20,HLA-DRB1*14:29, HLA-DRB1*14:83 |
| 53 | KMRHKRTST | 249-257 | 3 | HLA-DRB1*11:57, HLA-DRB1*12:04, HLA-DRB1*12:09 |
| 54 | YNKWLELLE | 344-352 | 3 | HLA-DRB1*12:12, HLA-DRB1*14:65, HLA-DRB1*14:74 |
| 55 | LLNRLQALE | 218-226 | 1 | HLA-DRB1*12:21 |
| 56 | LLEQNIDAY | 350-358 | 6 | HLA-DRB1*13:04, HLA-DRB1*13:75, HLA-DRB1*13:81, HLA-DRB1*13:89,HLA-DRB1*13:93, HLA-DRB1*13:94 |
| 57 | KSFNMVQAF | 258-266 | 3 | DRB1*13:04 DRB1*13:93 DRB1*13:94 |
| 58 | AFMGMSQFK | 305-313 | 4 | HLA-DRB1*13:21, HLA-DRB1*13:81, HLA-DRB1*13:88, HLA-DRB1*13:89, |
| 59 | GSITQRTRT | 390-398 | 2 | HLA-DRB1*13:58, HLA-DRB1*14:52 |
| 60 | IDAYKTFPK | 355-363 | 5 | HLA-DRB1*13:58, HLA-DRB1*13:81, HLA-DRB1*13:88, HLA-DRB1*13:89, HLA-DRB1*14:74 |
| 61 | VITKKDAAA | 237-245 | 5 | HLA-DRB1*13:81, HLA-DRB1*13:89, HLA-DRB1*13:94, HLA-DRB1*14:21, HLA-DRB1*14:78 |
| 62 | IKLDPKNPN | 335-343 | 3 | HLA-DRB1*13:81, HLA-DRB1*13:89, HLA-DRB1*13:94 |
| 63 | VSRNSSRSS | 178-186 | 1 | HLA-DRB1*13:81 |
| 64 | LNANSTPAQ | 65-73 | 2 | HLA-DRB1*13:81, HLA-DRB1*13:89 |
| 65 | HKRTSTKSF | 252-260 | 1 | HLA-DRB1*13:88 |
| 66 | VSWYTGLTQ | 41-49 | 1 | HLA-DRB1*13:88 |
| 67 | TKLPKNFHI | 154-162 | 2 | HLA-DRB1*13:88, HLA-DRB1*13:89 |
| 68 | RTSTKSFNM | 254-262 | 1 | HLA-DRB1*13:88 |
| 69 | YWRRQDRKI | 77-85 | 2 | HLA-DRB1*13:88, HLA-DRB1*14:13 |
| 70 | DITNTNLSR | 17-25 | 1 | HLA-DRB1*13:89 |
| 71 | LEQNIDAYK | 351-359 | 1 | HLA-DRB1*13:89 |
