# Supplementary material for: Epitope-Based Vaccine Target Screening against Highly Pathogenic MERS-CoV: An In Silico Approach Applied to Emerging Infectious Diseases
Source: PLoS One. 2015 Dec 7;10(12):e0144475. doi: 10.1371/journal.pone.0144475 (PMC4671582; doi:10.1371/journal.pone.0144475)
Supplement: S3 Table — (DOC) [file pone.0144475.s006.doc]

**Supplementary data**

**Table S3. 9-mer peptide sequences in Nucleocapsid (N) protein of MERS-CoV predicted to be CTL epitope using IEDB MHC-I Binding Prediction Web Server.**

| **Epitope No.** | **Epitope sequence (9 -mer)** | **Amino Acid Position** | **No. of binding HLA-A alleles** | **HLA-A alleles predicted to bind to the epitope** |
| --- | --- | --- | --- | --- |
| 1 | LAPRWYFYY | 94-102 | 34 | HLA-A*01:02, HLA-A*01:19, HLA-A*01:20, HLA-A*01:60 HLA-A*01:06, HLA-A*01:12, HLA-A*01:17, HLA-A*01:21, HLA-A*01:25, HLA-A*01:43, HLA-A*30:09 HLA-A*29:01, HLA-A*29:03, HLA-A*29:04, HLA-A*29:06, HLA-A*29:07, HLA-A*29:09, HLA-A*29:10, HLA-A*29:11, HLA-A*29:12, HLA-A*29:14, HLA-A*29:15, HLA-A*29:16, HLA-A*29:17, HLA-A*29:18, HLA-A*29:20, HLA-A*29:21, HLA-A*29:22, HLA-A*30:04, HLA-A*30:06, HLA-A*30:07, HLA-A*30:25, HLA-A*30:29, HLA-A*33:13 |
| 2 | QLAPRWYFY | 93-101 | 49 | HLA-A*01:06, HLA-A*01:12, HLA-A*01:19, HLA-A*01:21, HLA-A*01:25, HLA-A*11:11, HLA-A*03:41, HLA-A*01:51, HLA-A*11:40, HLA-A*11:45, HLA-A*29:01, HLA-A*29:02, HLA-A*29:03, HLA-A*29:04, HLA-A*29:05, HLA-A*29:06, HLA-A*29:07, HLA-A*29:09, HLA-A*29:10, HLA-A*29:11, HLA-A*29:12, HLA-A*29:13, HLA-A*29:14, HLA-A*29:15, HLA-A*29:16, HLA-A*29:17, HLA-A*29:18, HLA-A*29:19, HLA-A*29:20, HLA-A*29:21, HLA-A*29:22, HLA-A*30:03, HLA-A*30:04, HLA-A*30:06, HLA-A*30:07, HLA-A*30:09, HLA-A*30:10, HLA-A*30:12, HLA-A*30:22, HLA-A*30:28, HLA-A*30:29, HLA-A*30:32, HLA-A*30:33, HLA-A*30:34, HLA-A*33:13, HLA-A*36:02, HLA-A*74:10, HLA-A*80:01, HLA-A*80:02 |
| 3 | LLEQNIDAY | 350-358 | 5 | HLA-A*01:08, HLA-A*01:14, HLA-A*01:23, HLA-A*01:60, HLA-A*01:66 |
| 4 | YTGLTQHGK | 44-52 | 3 | HLA-A*01:13, HLA-A*01:28, HLA-A*03:18 |
| 5 | KLDPKNPNY | 336-344 | 2 | HLA-A*01:14, HLA-A*03:41, HLA-A*36:01, HLA-A*36:02, HLA-A*36:03, HLA-A*36:05 |
| 6 | FMGMSQFKL | 306-314 | 20 | HLA-A*02:02, HLA-A*02:07, HLA-A*02:110, HLA-A*02:112, HLA-A*02:12, HLA-A*02:124, HLA-A*02:130, HLA-A*02:136, HLA-A*02:146, HLA-A*02:16, HLA-A*02:184, HLA-A*02:19, HLA-A*02:219, HLA-A*02:231, HLA-A*02:247, HLA-A*02:255, HLA-A*02:33, HLA-A*02:50, HLA-A*02:81, HLA-A*02:87 |
| 7 | KINTGNGIK | 84-92 | 1 | HLA-A*03:43 |
| 8 | FLRYSGAIK | 328-336 | 1 | HLA-A*03:43 |
| 9 | LTFPPGQGV | 55-63 | 2 | HLA-A*02:78, HLA-A*68:54 |
| 10 | RLQALESGK | 221-229 | 1 | HLA-A*03:01, HLA-A*03:65, HLA-A*03:66 |
| 11 | AQNAGYWRR | 72-80 | 37 | HLA-A*03:32, HLA-A*31:01, HLA-A*31:02, HLA-A*31:03, HLA-A*31:04, HLA-A*31:05,  HLA-A*31:06, HLA-A*31:09, HLA-A*31:11, HLA-A*31:12, HLA-A*31:13, HLA-A*31:15,  HLA-A*31:16, HLA-A*31:17, HLA-A*31:18, HLA-A*31:19, HLA-A*31:20, HLA-A*31:21,  HLA-A*31:22, HLA-A*31:23, HLA-A*31:24, HLA-A*31:25, HLA-A*31:26, HLA-A*31:28,  HLA-A*31:29, HLA-A*31:30, HLA-A*31:31, HLA-A*31:32, HLA-A*31:33, HLA-A*31:34, HLA-A*31:35, HLA-A*31:36, HLA-A*31:37, HLA-A*33:08, HLA-A*33:09, HLA-A*33:21, HLA-A*74:07 |
| 12 | RVQGSITQR | 387-395 | 31 | HLA-A*03:32, HLA-A*31:01, HLA-A*31:06, HLA-A*31:09, HLA-A*31:11, HLA-A*31:12,  HLA-A*31:13, HLA-A*31:15, HLA-A*31:16, HLA-A*31:21, HLA-A*31:23, HLA-A*31:24,  HLA-A*31:25, HLA-A*31:26, HLA-A*31:27, HLA-A*31:28, HLA-A*31:31, HLA-A*31:33,  HLA-A*31:34, HLA-A*31:35, HLA-A*31:36, HLA-A*31:37, HLA-A*33:09, HLA-A*74:01,  HLA-A*74:02, HLA-A*74:03, HLA-A*74:05, HLA-A*74:07, HLA-A*74:08, HLA-A*74:09,  HLA-A*74:11 |
| 13 | AALPFRAVK | 109-117 | 25 | HLA-A*03:72, HLA-A*11:22, HLA-A*11:24, HLA-A*11:31, HLA-A*11:35, HLA-A*11:47, HLA-A*11:59, HLA-A*30:08, HLA-A*30:11, HLA-A*30:15, HLA-A*30:16, HLA-A*30:18,  HLA-A*30:19, HLA-A*30:20, HLA-A*30:23, HLA-A*30:24, HLA-A*30:26, HLA-A*30:30,  HLA-A*30:31, HLA-A*30:35, HLA-A*30:36, HLA-A*30:37, HLA-A*30:39, HLA-A*30:40,  HLA-A*30:41 |
| 14 | AFMGMSQFK | 305-313 | 3 | HLA-A*11:36, HLA-A*11:43, HLA-A*30:26 |
| 15 | LYLDLLNRL | 214-222 | 7 | HLA-A*23:01, HLA-A*24:03, HLA-A*24:04, HLA-A*24:109, HLA-A*24:18, HLA-A*24:28, HLA-A*24:89 |
| 16 | NYNKWLELL | 343-351 | 128 | HLA-A*23:02, HLA-A*23:03, HLA-A*23:04, HLA-A*23:05, HLA-A*23:06, HLA-A*23:10, HLA-A*23:14, HLA-A*23:15, HLA-A*23:16, HLA-A*23:17, HLA-A*23:18, HLA-A*23:20, HLA-A*23:21, HLA-A*23:22, HLA-A*23:23, HLA-A*23:24, HLA-A*23:25, HLA-A*23:26, HLA-A*24:04, HLA-A*24:05, HLA-A*24:06, HLA-A*24:07, HLA-A*24:08, HLA-A*24:10, HLA-A*24:100, HLA-A*24:101, HLA-A*24:102, HLA-A*24:103, HLA-A*24:105, HLA-A*24:107, HLA-A*24:108, HLA-A*24:109, HLA-A*24:110, HLA-A*24:111, HLA-A*24:112, HLA-A*24:113, HLA-A*24:114, HLA-A*24:115, HLA-A*24:116, HLA-A*24:117, HLA-A*24:118, HLA-A*24:119, HLA-A*24:120, HLA-A*24:122, HLA-A*24:123, HLA-A*24:124, HLA-A*24:125, HLA-A*24:126, HLA-A*24:127, HLA-A*24:128, HLA-A*24:129, HLA-A*24:13, HLA-A*24:130, HLA-A*24:131, HLA-A*24:133, HLA-A*24:134, HLA-A*24:135, HLA-A*24:136, HLA-A*24:137, HLA-A*24:138, HLA-A*24:139, HLA-A*24:140, HLA-A*24:141, HLA-A*24:142, HLA-A*24:143, HLA-A*24:144, HLA-A*24:15, HLA-A*24:19, HLA-A*24:20, HLA-A*24:21, HLA-A*24:22, HLA-A*24:23, HLA-A*24:24, HLA-A*24:25, HLA-A*24:27,  HLA-A*24:28, HLA-A*24:29, HLA-A*24:30, HLA-A*24:31, HLA-A*24:32, HLA-A*24:33, HLA-A*24:34, HLA-A*24:35, HLA-A*24:37, HLA-A*24:38, HLA-A*24:39,  HLA-A*24:43, HLA-A*24:44, HLA-A*24:46, HLA-A*24:47, HLA-A*24:49, HLA-A*24:51,  HLA-A*24:52, HLA-A*24:53, HLA-A*24:54, HLA-A*24:55, HLA-A*24:56, HLA-A*24:57,  HLA-A*24:58, HLA-A*24:59, HLA-A*24:61, HLA-A*24:62, HLA-A*24:63, HLA-A*24:64,  HLA-A*24:67, HLA-A*24:68, HLA-A*24:69, HLA-A*24:70, HLA-A*24:71, HLA-A*24:72,  HLA-A*24:73, HLA-A*24:74, HLA-A*24:80, HLA-A*24:81, HLA-A*24:82, HLA-A*24:85,  HLA-A*24:87, HLA-A*24:89, HLA-A*24:91, HLA-A*24:92, HLA-A*24:93, HLA-A*24:94,  HLA-A*24:95, HLA-A*24:96, HLA-A*24:97, HLA-A*24:98, HLA-A*24:99, HLA-A*33:19 |
| 17 | KQLAPRWYF | 92-100 | 138 | HLA-A*23:03, HLA-A*23:04, HLA-A*23:05, HLA-A*23:06, HLA-A*23:10, HLA-A*23:12, HLA-A*23:13, HLA-A*23:14, HLA-A*23:15, HLA-A*23:16, HLA-A*23:17, HLA-A*23:18, HLA-A*23:20, HLA-A*23:21, HLA-A*23:22, HLA-A*23:23, HLA-A*23:24, HLA-A*23:25, HLA-A*23:26, HLA-A*24:05, HLA-A*24:08, HLA-A*24:100, HLA-A*24:101, HLA-A*24:102, HLA-A*24:103, HLA-A*24:105, HLA-A*24:106, HLA-A*24:107, HLA-A*24:110, HLA-A*24:111, HLA-A*24:113, HLA-A*24:114, HLA-A*24:115, HLA-A*24:116, HLA-A*24:117, HLA-A*24:118, HLA-A*24:120, HLA-A*24:121, HLA-A*24:122, HLA-A*24:123, HLA-A*24:124, HLA-A*24:125,HLA-A*24:126, HLA-A*24:127, HLA-A*24:128, HLA-A*24:13, HLA-A*24:130, HLA-A*24:133, HLA-A*24:134, HLA-A*24:135,HLA-A*24:137, HLA-A*24:139, HLA-A*24:14, HLA-A*24:140, HLA-A*24:141, HLA-A*24:142, HLA-A*24:143, HLA-A*24:144, HLA-A*24:15, HLA-A*24:17, HLA-A*24:18, HLA-A*24:20, HLA-A*24:21, HLA-A*24:23, HLA-A*24:25, HLA-A*24:26, HLA-A*24:27, HLA-A*24:29, HLA-A*24:30, HLA-A*24:32, HLA-A*24:33, HLA-A*24:34, HLA-A*24:35, HLA-A*24:37, HLA-A*24:38, HLA-A*24:39, HLA-A*24:41, HLA-A*24:42, HLA-A*24:43, HLA-A*24:44, HLA-A*24:47, HLA-A*24:49, HLA-A*24:51, HLA-A*24:52, HLA-A*24:53, HLA-A*24:54, HLA-A*24:55, HLA-A*24:56, HLA-A*24:57, HLA-A*24:58, HLA-A*24:61,HLA-A*24:62, HLA-A*24:63, HLA-A*24:64, HLA-A*24:66, HLA-A*24:68, HLA-A*24:69, HLA-A*24:70, HLA-A*24:71, HLA-A*24:72, HLA-A*24:73, HLA-A*24:74, HLA-A*24:80, HLA-A*24:81, HLA-A*24:85, HLA-A*24:87, HLA-A*24:91, HLA-A*24:92, HLA-A*24:93, HLA-A*24:94, HLA-A*24:95, HLA-A*24:96, HLA-A*24:97, HLA-A*24:98, HLA-A*24:99, HLA-A*29:13, HLA-A*30:09,  HLA-A*31:07, HLA-A*31:08, HLA-A*31:10, HLA-A*32:02, HLA-A*32:03, HLA-A*32:04,  HLA-A*32:05, HLA-A*32:06, HLA-A*32:08, HLA-A*32:09, HLA-A*32:12, HLA-A*32:13,  HLA-A*32:14, HLA-A*32:17, HLA-A*32:18, HLA-A*32:20, HLA-A*32:21, HLA-A*32:22,  HLA-A*32:23, HLA-A*32:24, HLA-A*32:25 |
| 18 | ELAPTASAF | 298-306 | 53 | HLA-A*25:02, HLA-A*25:03, HLA-A*25:04, HLA-A*25:05, HLA-A*25:06, HLA-A*25:07,  HLA-A*25:08, HLA-A*25:09, HLA-A*25:10, HLA-A*25:11, HLA-A*25:13, HLA-A*26:01,  HLA-A*26:04, HLA-A*26:05, HLA-A*26:08, HLA-A*26:09, HLA-A*26:10, HLA-A*26:12,  HLA-A*26:13, HLA-A*26:14, HLA-A*26:15, HLA-A*26:16, HLA-A*26:17, HLA-A*26:18, HLA-A*26:20, HLA-A*26:22, HLA-A*26:23, HLA-A*26:24, HLA-A*26:26,  HLA-A*26:27, HLA-A*26:28, HLA-A*26:29, HLA-A*26:31, HLA-A*26:33, HLA-A*26:34,  HLA-A*26:35, HLA-A*26:36, HLA-A*26:37, HLA-A*26:38, HLA-A*26:39, HLA-A*26:40,  HLA-A*26:41, HLA-A*26:42, HLA-A*26:43, HLA-A*26:45, HLA-A*26:46, HLA-A*26:47,  HLA-A*26:48, HLA-A*26:49, HLA-A*26:50, HLA-A*43:01, HLA-A*66:03, HLA-A*66:11 |
| 19 | SAFMGMSQF | 304-312 | 6 | HLA-A*25:02, HLA-A*25:04, HLA-A*25:06, HLA-A*25:11, HLA-A*32:10, HLA-A*68:36 |
| 20 | NTVSWYTGL | 39-47 | 45 | HLA-A*25:02, HLA-A*25:03, HLA-A*25:04, HLA-A*25:05, HLA-A*25:07, HLA-A*25:08,  HLA-A*25:09, HLA-A*25:10, HLA-A*25:11, HLA-A*25:13, HLA-A*26:02, HLA-A*26:06,  HLA-A*26:20, HLA-A*26:21, HLA-A*26:30, HLA-A*26:33, HLA-A*33:19, HLA-A*34:01,  HLA-A*34:05, HLA-A*34:06, HLA-A*66:02, HLA-A*66:03, HLA-A*66:05, HLA-A*66:06,  HLA-A*66:07, HLA-A*66:08, HLA-A*66:09, HLA-A*66:10, HLA-A*66:11, HLA-A*66:12, HLA-A*66:13, HLA-A*66:14, HLA-A*66:15, HLA-A*68:15, HLA-A*68:27, HLA-A*68:28,  HLA-A*68:31, HLA-A*68:34, HLA-A*68:40, HLA-A*68:44, HLA-A*68:48, HLA-A*68:51,  HLA-A*68:53, HLA-A*68:54, HLA-A*69:01 |
| 21 | STPAQNAGY | 69-77 | 60 | HLA-A*25:02, HLA-A*25:04, HLA-A*25:07, HLA-A*25:08, HLA-A*25:09, HLA-A*25:10,  HLA-A*25:11, HLA-A*25:13, HLA-A*26:01, HLA-A*26:04, HLA-A*26:05, HLA-A*26:06,  HLA-A*26:07, HLA-A*26:08, HLA-A*26:09, HLA-A*26:10, HLA-A*26:12, HLA-A*26:13,  HLA-A*26:14, HLA-A*26:15, HLA-A*26:16, HLA-A*26:17, HLA-A*26:18, HLA-A*26:19,  HLA-A*26:20, HLA-A*26:21, HLA-A*26:22, HLA-A*26:23, HLA-A*26:24, HLA-A*26:26,  HLA-A*26:27, HLA-A*26:28, HLA-A*26:29, HLA-A*26:30, HLA-A*26:31, HLA-A*26:32,  HLA-A*26:33, HLA-A*26:34, HLA-A*26:35, HLA-A*26:36, HLA-A*26:37, HLA-A*26:38,  HLA-A*26:39, HLA-A*26:40, HLA-A*26:41, HLA-A*26:42, HLA-A*26:43, HLA-A*26:45,  HLA-A*26:46, HLA-A*26:47, HLA-A*26:48, HLA-A*26:49, HLA-A*26:50, HLA-A*30:04,  HLA-A*30:06, HLA-A*36:04, HLA-A*43:01, HLA-A*66:03, HLA-A*66:05, HLA-A*66:11 |
| 22 | PTASAFMGM | 301-309 | 2 | HLA-A*26:02, HLA-A*26:03 |
| 23 | GAVGGDLLY | 207-215 | 1 | HLA-A*29:02 |
| 24 | KSFNMVQAF | 258-266 | 26 | HLA-A*29:13, HLA-A*31:07, HLA-A*31:08, HLA-A*31:10, HLA-A*32:03, HLA-A*32:04,  HLA-A*32:01, HLA-A*32:02, HLA-A*32:05, HLA-A*32:06, HLA-A*32:08, HLA-A*32:09,  HLA-A*32:10, HLA-A*32:12, HLA-A*32:13, HLA-A*32:14, HLA-A*32:16, HLA-A*32:17, HLA-A*32:18, HLA-A*32:20, HLA-A*32:21, HLA-A*32:22, HLA-A*32:23, HLA-A*32:24, HLA-A*32:25, HLA-A*74:10 |
| 25 | HGNPVYFLR | 322-330 | 76 | HLA-A*29:19, HLA-A*30:17, HLA-A*31:01, HLA-A*31:02, HLA-A*31:03, HLA-A*31:04,  HLA-A*31:05, HLA-A*31:06, HLA-A*31:09, HLA-A*31:11, HLA-A*31:12, HLA-A*31:13,  HLA-A*31:15, HLA-A*31:16, HLA-A*31:17, HLA-A*31:18, HLA-A*31:19, HLA-A*31:20,  HLA-A*31:21, HLA-A*31:22, HLA-A*31:23, HLA-A*31:24, HLA-A*31:25, HLA-A*31:26,  HLA-A*31:27, HLA-A*31:28, HLA-A*31:29, HLA-A*31:30, HLA-A*31:31, HLA-A*31:32,  HLA-A*31:33, HLA-A*31:34, HLA-A*31:35, HLA-A*31:37, HLA-A*33:03, HLA-A*33:04,  HLA-A*33:05, HLA-A*33:06, HLA-A*33:07, HLA-A*33:08, HLA-A*33:09, HLA-A*33:10,  HLA-A*33:11, HLA-A*33:12, HLA-A*33:14, HLA-A*33:15, HLA-A*33:16, HLA-A*33:17,  HLA-A*33:18, HLA-A*33:20, HLA-A*33:21, HLA-A*33:22, HLA-A*33:23, HLA-A*33:24,  HLA-A*33:25, HLA-A*33:26, HLA-A*33:27, HLA-A*33:29, HLA-A*33:30, HLA-A*33:31,  HLA-A*68:03, HLA-A*68:04, HLA-A*68:10, HLA-A*68:13, HLA-A*68:14, HLA-A*68:29,  HLA-A*68:45, HLA-A*74:01, HLA-A*74:02, HLA-A*74:03, HLA-A*74:05, HLA-A*74:06,  HLA-A*74:07, HLA-A*74:08, HLA-A*74:09, HLA-A*74:11 |
| 26 | RTRPSVQPG | 397-405 | 20 | HLA-A*30:01, HLA-A*30:16, HLA-A*30:18, HLA-A*30:30, HLA-A*30:38, HLA-A*30:08, HLA-A*30:11, HLA-A*30:15, HLA-A*30:19, HLA-A*30:20, HLA-A*30:23,  HLA-A*30:24, HLA-A*30:31, HLA-A*30:35, HLA-A*30:36, HLA-A*30:37, HLA-A*30:39,  HLA-A*30:40, HLA-A*30:41 |
| 27 | RGRNPKPRA | 27-35 | 15 | HLA-A*30:01, HLA-A*30:11, HLA-A*30:15, HLA-A*30:18, HLA-A*30:19, HLA-A*30:20,  HLA-A*30:23, HLA-A*30:24, HLA-A*30:30, HLA-A*30:35, HLA-A*30:36, HLA-A*30:37,  HLA-A*30:39, HLA-A*30:40, HLA-A*30:41 |
| 28 | KVKQSQPKV | 229-237 | 19 | HLA-A*30:01, HLA-A*30:08, HLA-A*30:11, HLA-A*30:15, HLA-A*30:16, HLA-A*30:18,  HLA-A*30:19, HLA-A*30:20, HLA-A*30:23, HLA-A*30:24, HLA-A*30:30, HLA-A*30:31, HLA-A*30:35, HLA-A*30:36, HLA-A*30:37, HLA-A*30:38, HLA-A*30:39, HLA-A*30:40,  HLA-A*30:41 |
| 29 | AAKNKMRHK | 245-253 | 17 | HLA-A*30:01, HLA-A*30:11, HLA-A*30:15, HLA-A*30:18, HLA-A*30:19, HLA-A*30:20,  HLA-A*30:23, HLA-A*30:24, HLA-A*30:30, HLA-A*30:31, HLA-A*30:35, HLA-A*30:36,  HLA-A*30:37, HLA-A*30:38, HLA-A*30:39, HLA-A*30:40, HLA-A*30:41 |
| 30 | SSRASSVSR | 172-180 | 24 | HLA-A*30:08, HLA-A*30:11, HLA-A*30:13, HLA-A*30:15, HLA-A*30:16,HLA-A*30:17,  HLA-A*30:18, HLA-A*30:19, HLA-A*30:20, HLA-A*30:23, HLA-A*30:24, HLA-A*30:26,  HLA-A*30:30, HLA-A*30:31, HLA-A*30:35, HLA-A*30:36, HLA-A*30:37, HLA-A*30:38,  HLA-A*30:39, HLA-A*30:40, HLA-A*30:41, HLA-A*31:15, HLA-A*31:24, HLA-A*31:30 |
| 31 | SSVSRNSSR | 176-184 | 3 | HLA-A*31:24, HLA-A*33:28, HLA-A*68:42 |
| 32 | NMVQAFGLR | 261-269 | 2 | HLA-A*33:17, HLA-A*33:24 |
| 33 | DAPSTFGTR | 130-138 | 1 | HLA-A*66:04 |
| 34 | EAALPFRAV | 108-116 | 13 | HLA-A*68:02, HLA-A*68:15, HLA-A*68:27, HLA-A*68:28, HLA-A*68:31, HLA-A*68:34,  HLA-A*68:40, HLA-A*68:44, HLA-A*68:48, HLA-A*68:51, HLA-A*68:53, HLA-A*68:54,  HLA-A*69:01 |
